# Core–Shell Upconversion Platform Based on an Amino-Functionalized ZIF‑8 with a Covalently Anchored Ruthenium Nitrosyl Complex for Controlled Release of Nitric Oxide in Biological Media

**DOI:** 10.1021/acsomega.6c08430

**Published:** 2026-10-14

**Authors:** Victor B. de Sousa, Giovanna N. S. A. O. Rocha, Dayane K. D. Do N. Santos, Francisco V. S. Leite, Walysson G. Pereira, Luiz G. F. Lopes, Eduardo H. S. de Sousa, Ricardo L. Longo, Severino A. Júnior

**Affiliations:** † Department of Fundamental Chemistry, Federal University of Pernambuco, Recife, Pernambuco 50740-560, Brazil; ‡ Department of Organic and Inorganic Chemistry, Federal University of Ceará, Fortaleza, Ceará 60440-900, Brazil

## Abstract

The development of nitric oxide (NO) donor agents capable of controlled NO release upon light irradiation in the biological window is relevant for phototherapies. The *cis*-[Ru­(dc-bpy)_2_(SO_3_)­(NO)]^+^ complex (dc-bpy = 2,2′-dicarboxybipyridine) is synthesized and anchored onto a NaYF_4_:Yb,Tm@ZIF-8-NH_2_ nanoplatform. The isolated complex presents high stability under basic conditions (pH 9) and an absorption band in the visible region up to 405 nm. A core–shell platform of upconversion nanoparticles (UCNPs) coated with ZIF-8 is synthesized and functionalized with amino groups (UCNP-ZIF-8-NH_2_). This material exhibits intense blue emission upon excitation by near-infrared (NIR) radiation at 980 nm within a biological window. After covalent anchoring, through amide bond between the amino groups of UCNP-ZIF-8-NH_2_ and the carboxylic acid groups of the RuNO complex, supported by Fourier transform spectrophotometry (FTIR) and X-ray photoelectron spectroscopy (XPS) analyses, the UCNP-RuNO composite releases NO under 980 nm irradiation. NO release is controlled by irradiation time, showing an increase from ca. 0.095 to 1.015 μmol L^–1^ between 30 and 180 min. These UCNP-RuNO systems show in vitro cell viability up to ca. 12 μg mL^–1^. These results support their use as platforms for NO release under NIR light, with optimization achievable by varying concentration, formulation, excitation intensity, and UCNP composition.

## Graphical abstract

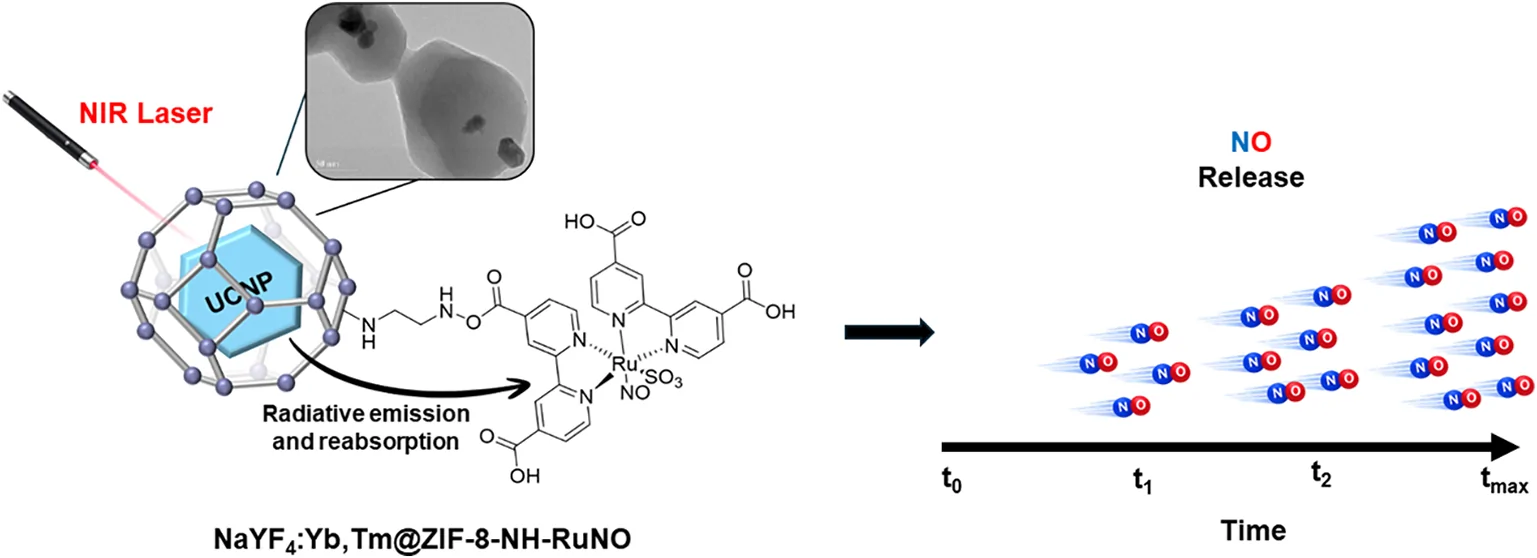

## 1 Introduction

Nitric oxide or nitrogen monoxide (NO) is a simple molecule that plays a fundamental role in the biological system, working mainly in the nervous and cardiovascular systems.^1^ Several studies showed that NO, above a certain amount, can trigger various effects in mammals.^2^ At concentrations smaller than 200 nmol L^–1^, NO induces muscle relaxation, controls neurotransmitters release, among other processes in the human body.^3^ When NO is presented at concentrations larger than 200 nmol L^–1^, it can promote DNA damage, causing protein dysfunction, and cell death by apoptosis.^3,4^ This induction of cell apoptosis provides a promising role in tumor therapy. However, NO can cause both effects either tumor growth or death and inhibition of cancer metastasis, which is strictly dependent on NO concentrations.^4,5^

The use of NO in cancer therapy has expanded with the development of several classes of compounds capable of releasing nitric oxide.^6^ Currently, there are many compounds such as organic nitrites and nitrates,^7^ nitrobenzenes,^8,9^ nitrosothiols,^10^ and transition-metal coordination compounds^11–14^ reported in the literature. However, some issues have emerged, making it harder to consolidate this new therapeutic perspective. One of the main obstacles is the controlled release of NO from either organic or inorganic sources. In fact, appropriate inorganic sources could photorelease NO in a controlled manner, but they are mostly dependent on the use of ultraviolet (UV) radiation.^3,9^

Photodynamic therapy (PDT) has been used in cancer treatment by using light and a photosensitizer to cause cell death.^15,16^ This phototherapeutic approach could be used for the controlled release of NO as well. From this perspective, nitrosyl iron compounds such as sodium nitroprusside, Na_2_[Fe­(NO)­(CN)_5_],^17,18^ and Roussin’s salts initially stood out.^19,20^ However, these compounds showed spontaneous release of NO, and due to the lability of the cyanide groups, there was a significant increase in side effects.^21,22^ To mitigate these problems, chelating ligands^23,24^ and metals that bind strongly to the nitrosyl groups began to be used with, hopefully, less harmful effects after irradiation.^25^

Among the transition metals for photorelease of NO, ruthenium compounds have been shown to be more stable, easier to perform structural modifications, and lower toxicity.^3,25^ Studies have also demonstrated that the photorelease depends on the oxidation state, where only Ru^2+^-nitrosyl complexes can release NO.^22^ A problem associated with the use of these compounds is their dependence on UV radiation for effective release of NO, which is a harmful radiation for biological systems.^26–28^

With the production of ruthenium nitrosyl complexes absorbing in the visible spectrum, it is possible to combine such a compound with upconversion nanoparticles (UCNPs) as potential materials for the treatment of malignant neoplasms. UCNPs can convert near-infrared (NIR) photons in the first (NIR-I: 700–1000 nm) or second (NIR-II: 1000–1700 nm) biological windows, which have a deeper skin penetration,^29^ into high-energy photons in the UV–visible range.^30^ Aiming at different types of excitations, the upconversion sensitized by Yb^3+^ ions has demonstrated high upconversion energy efficiency. This is due to the large absorption cross-section (1.2 × 10^–20^ cm^2^) of the ^2^F_7/2_ → ^2^F_5/2_ transition at 980 nm, so these ions can efficiently sensitize Er^3+^, Tm^3+^, and Ho^3+^ ions.^31–33^

There are many possible combinations to produce upconversion nanoparticles, with NaYF_4_-based UCNPs doped with Yb^3+^ and Tm^3+^ ions, NaYF_4_:Yb,Tm, being applied as excellent drug delivery system.^34^ These NaYF_4_:Yb,Tm nanoparticles can convert NIR into light in the violet and blue region.^35,36^ To improve the features of these nanoparticles, surface coating strategies have begun to be applied as well.^37^

To cover the surface of UCNPs, metal organic structures (MOFs) have been explored.^38^ Indeed, ZIF-8 (zeolitic imidazolate structure 8) is particularly relevant because of its biocompatibility, high surface area (1700 m^2^/g), high thermal stability (400 °C), low density, high porosity (with pores of 3.4 Å), and cavities of 11.6 Å.^39–42^ So, ZIFs are quite suitable as platforms for controlled drug delivery.^43,44^ Another feature of ZIFs is their surface functionalization, enabling them to interact with specific targets.^45,46^ Thus, functionalized ZIF-8 is a strong candidate for covering UCNPs and anchoring ruthenium complexes for controlled release of nitric oxide.

A system capable of performing energy upconversion with surface modifications was developed and conjugated to a ruthenium nitrosyl compound for the controlled release of nitric oxide. The NaYF_4_:Yb,Tm particle was coated with a ZIF-8 to obtain a UCNPs@ZIF-8 composite, and its surface was functionalized with amino groups, yielding the NaYF_4_:Yb,Tm@ZIF-8-NH_2_ system. Furthermore, the *cis*-[Ru­(dc-bpy)_2_(NO)­(SO_3_)]^+^ complex already exhibited a modest absorption in the visible region. Thus, our target system composed of NaYF_4_:Yb,Tm@ZIF-8-NH-[Ru­(dc-bpy)_2_(NO)­(SO_3_)]^+^, denoted as UCNP-RuNO, was synthesized and applied to the controlled NO release upon excitation at the NIR biological window (Figure 1).

**Figure 1:**
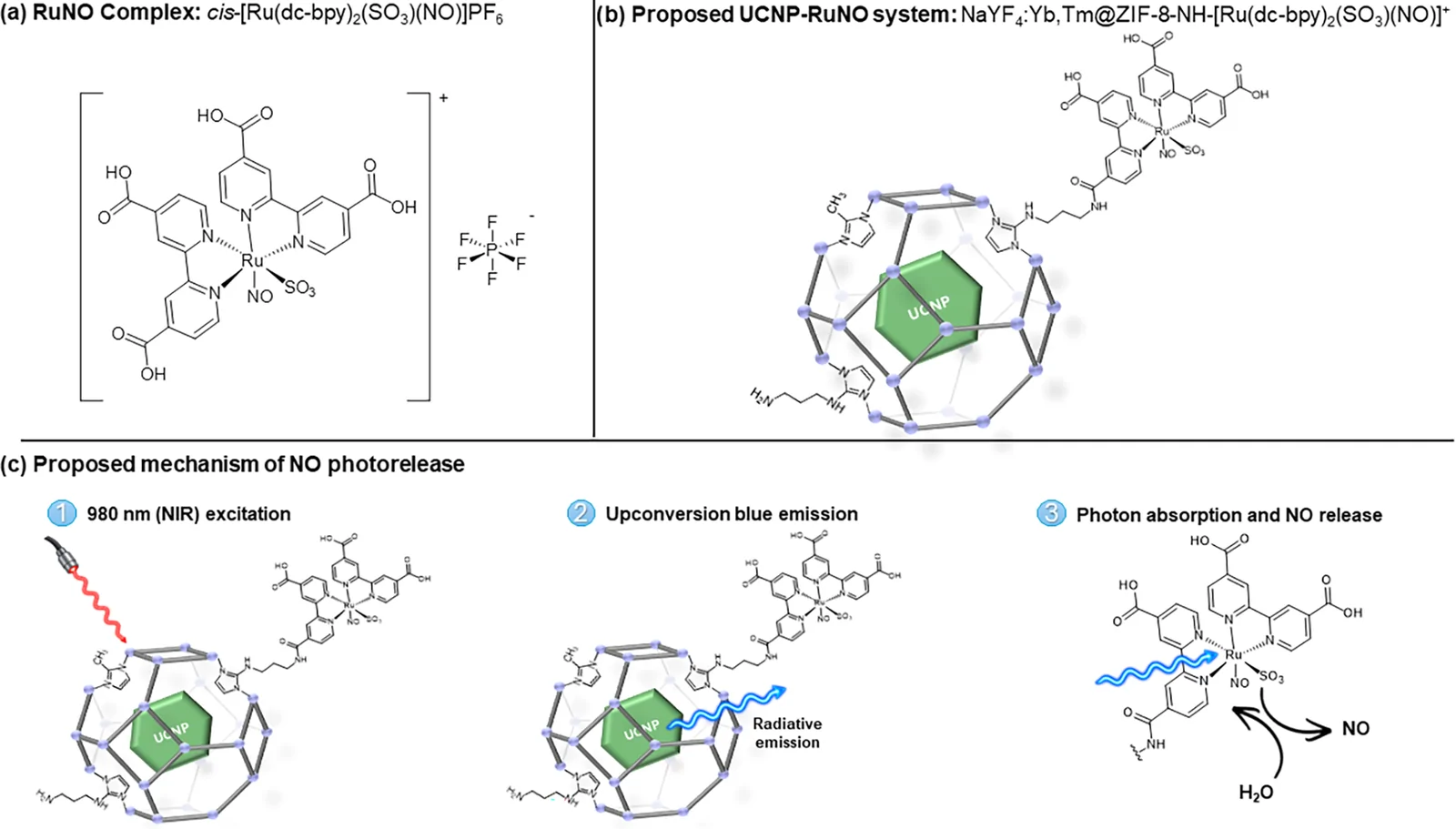
Schematic representation of (a) the RuNO complex, (b) the proposed UCNP-RuNO system, and (c) the proposed mechanism of NO photorelease under 980 nm irradiation. In the proposed mechanism, NIR excitation of the UCNPs generates higher-energy photons in the blue spectral region, which can be absorbed by the anchored RuNO complex, promoting photoactivation and subsequent NO release.

## 2 Experimental Section

### 2.1 Chemicals

Y_2_O_3_, Yb_2_O_3_, Tm_2_O_3_ (purity >99.99%), 1-octadecene (1-ODE, 90%), 2-methylimidazole (M-Im), ethylenediamine (EDA, 99%), 2-(4-carboxyphenyl)-4,4,5,5-tetramethylimidazoline-1-oxyl-3-oxide potassium salt (cPTIO), and polyvinylpyrrolidone (average molecular weight 10,000) were purchased from Sigma-Aldrich. Sulfuric acid (H_2_SO_4_), hydrochloric acid (HCl), and oleic acid (OA, 99%) were acquired from Merck. Zinc nitrate hexahydrate Zn­(NO_3_)_2_·6H_2_O (99.99%) and hexane (95%) were obtained from Dynamics. 4,4′-dimethyl-2,2′-bipyridine (99%), sodium nitrite NaNO_2_ (99%), sodium hydroxide NaOH (99%), ammonium hexafluorophosphate NH_4_PF_6_ (99%), lithium chloride LiCl (98%), sodium sulfite Na_2_SO_3_ (99%), and sodium dichromate Na_2_Cr_2_O_7_ (99%) were purchased from Vetec. Methanol, *N*,*N*-dimethylformamide (DMF), and ethyl ether were procured from Synth. Ethanol was from Biograde, ruthenium trichloride hydrate RuCl_3_·*x*H_2_O (≥99%) was purchased from Precious, and trifluoroacetic acid (≥99%) was obtained from Tedia. All aqueous solutions were prepared using ultrapure water (resistivity ≥ 18.25 MΩ·cm, Milli-Q system, Millipore).

### 2.2 Experimental Section

Infrared spectra were obtained using a Fourier transform spectrophotometer (FT-IR) ABB Bommen, model FTLA2000-102. Electronic spectra were obtained in aqueous solution with a Hewlett-Packard UV/vis spectrophotometer (PerkinElmer, model Lambda650) and Varian spectrophotometer UV/vis-NIR (model Cary 5000). The upconversion measurements were performed with a diode laser at 980 nm and 1200 mW power using an Ocean Optics optical fiber type UV/SR with a diameter of 600 μm and the spectra were obtained using the Horiba Jobin Yvon Fluorolog-3 ISA spectrofluorimeter. Powder X-ray diffraction (PXRD) patterns were determined using a Rigaku X-ray diffractometer, model SmartLab with a D/Tex Ultra 250 detector with Cu Kα radiation (1.537 Å), and the 2θ range used was from 05 to 80° in steps of 0.02°, with a counting time of 1.2 s. The morphology of the samples was analyzed using a scanning electron microscope (TESCAN MIRA 3), with an acceleration of 10 kV and a transmission electron microscope (JEM-2100, Jeol, Tokyo, Japan), equipped with EDS, Thermo scientific, and an acceleration of 200 kV. The voltammogram was obtained using an electrochemical system model EC Epsilon (BASi) in an aqueous solution of 0.1 mol L^–1^ of trifluoroacetic acid (HTFA). X-ray photoelectron spectroscopy (XPS) measurements were performed using a Scienta-Omicron ESCA+ spectrometer equipped with a monochromated Al Kα X-ray source (1486.6 eV), a high-performance hemispherical analyzer (EAC-2000), and a low-energy electron source for charge compensation. The technique was employed to investigate the surface chemical composition and bonding environment of the UCNP-RuNO material. Survey and high-resolution spectra were acquired. High-resolution C 1s, N 1s, and O 1s spectra were analyzed using CasaXPS with a Shirley background and GL(30) Gaussian–Lorentzian line shapes (70% Gaussian and 30% Lorentzian contributions). Peak areas were left unconstrained during fitting.

### 2.3 Synthesis of *cis*-[Ru­(dc-bpy)_2_(NO)­(SO_3_)]­PF_6_

The syntheses of 4,4′-dicarboxy-2,2′-bipyridine (dc-bpy) ligand and of the precursor *cis*-[Ru­(dc-bpy)_2_(Cl)_2_] complex are available in the Supporting Information. These compounds were used to prepare the complex of interest following the methodology of Silva et al. with some modifications.^47^ In a round-bottom flask, it was mixed 1 mmol (0.69 mg) of *cis*-dichlorobis­(dicarboxypyridine)­ruthenium­(II), *cis*-[Ru­(Cl)_2_(dc-bpy)_2_], 1 mmol (0.12 mg) of sodium sulfite, Na_2_SO_3_, and 10 mL of ethanol:water (1:1) solution. This solution was placed under reflux for 2 h. After this period, 1 mmol (0.069 mg) of sodium nitrite, NaNO_2_, was added into this solution and kept for another 2 h under reflux. Afterward, this solution was cooled down to 26 °C and 5 mL of trifluoroacetic acid was added, while this mixture was kept under argon flow and stirring for 2 h. A saturated solution of ammonium hexafluorophosphate, NH_4_PF_6_, was added into this mixture. This solution was rotary evaporated to dryness, resolubilized in 5 mL of methanol and precipitated by adding 100 mL of ethyl ether. A solid was collected by centrifugation at 5100 rpm for 5 min, which was washed several times with 20 mL ethanol:ether (1:5) solution.

### 2.4 Synthesis of NaYF_4_:Yb,Tm@ZIF-8-NH-[Ru­(dc-bpy)_2_(NO)­(SO_3_)]­PF_6_ (UCNP-RuNO)

The syntheses of NaYF_4_:Yb,Tm, NaYF_4_:Yb,Tm capped PVP, NaYF_4_:Yb,Tm@ZIF-8, and NaYF_4_:Yb,Tm@ZIF-8-NH_2_ are available in the Supporting Information. The procedure for obtaining the conjugated (or anchored) Ru^2+^ complex and the core–shell UCNP-ZIF-8 is based on the formation of amides between amine groups on the modified surface of ZIF-8 and carboxylic groups of the dc-bpy ligand. In this procedure, 30 mg of the NaYF_4_:Yb,Tm@ZIF-8-NH_2_ was added to 50 mL of methanol in an Erlenmeyer flask, and an ultrasound probe was placed for 30 min. After these particles were dispersed, this system was placed under magnetic stirring. Then, a solution with 100 mg of *cis*-[Ru­(dc-bpy)_2_(NO)­(SO_3_)]­PF_6_ in 50 mL of methanol was prepared, and the previous system in suspension was added. This reaction was kept for 24 h, a solid was recovered by centrifugation at 5100 rpm for 15 min, washed several times with methanol, and dried at room temperature.

### 2.5 Equilibrium Constant for the NO^+^/NO_2_^–^ Conversion

The interconversion of nitrosyl to nitro ligand for the *cis*-[Ru­(dc-bpy)_2_(NO)­(SO_3_)]­PF_6_ compound was studied by determining the changes of the UV–vis absorption spectrum with pH and quantified by monitoring the absorbance at 449 nm with the pH. An aqueous solution of the complex *cis*-[Ru­(dc-bpy)_2_(NO)­(SO_3_)]^+^ (1.1 × 10^–5^ mol L^–1^) in HTFA (0.1 mol L^–1^), maintaining a constant ionic strength, was prepared and titrated with a NaOH solution (0.05 mol L^–1^), with the pH determined with a pH-meter (pH-meter QUIMIS, model Q400AS). The titration curve was analyzed quantitatively by fitting a model describing the equilibrium shown in eq 1

1 $${{[\mathrm{Ru}\left(\mathrm{dc}‐\mathrm{bpy}\right)}_{2}\left({\mathrm{SO}}_{3}\right)\left(\mathrm{NO}\right)]}^{+}\left(\mathrm{aq}\right)+2{\mathrm{OH}}^{-}\left(\mathrm{aq}\right)={[\mathrm{Ru}\left(\mathrm{dc}‐\mathrm{bpy}{)}_{2}\left({\mathrm{SO}}_{3}\right)\left({\mathrm{NO}}_{2}\right)\right]}^{-}\left(\mathrm{aq}\right)+H_{2}O\left(l\right)$$

### 2.6 Nitric Oxide Photorelease from the *cis*-[Ru­(dc-bpy)_2_(NO)­(SO_3_)]^+^ Complex

The release of NO by cis-[Ru­(dc-bpy)_2_(NO)­(SO_3_)]^+^ under blue-light irradiation was determined by monitoring the UV–vis spectrum of a solution containing a known amount of cPTIO. Stock solutions of cPTIO (20.0 mM) and cis-[Ru­(dc-bpy)_2_(SO_3_)­(NO)]­PF_6_ (6.0 mM) were used. For the experiment, 100 μL of the complex stock solution and 30 μL of the cPTIO stock solution were added to phosphate buffer solution (0.1 mol L^–1^, pH 7.4 at 25 °C) to a final volume of 3.0 mL, resulting in final concentrations of 2.0 × 10^–4^ mol L^–1^ for both species. The solution was irradiated for up to 3 h using a commercial SMD blue LED floodlight (nominal electrical power: 200 W; nominal luminous flux: 18,000 lm), positioned 30 cm above the sample at normal incidence (∼90°). The absorbance at 560 nm was monitored as a function of irradiation time.

### 2.7 Nitric Oxide Photorelease from the NaYF_4_:Yb,Tm@ZIF-8-NH-[Ru­(dc-bpy)_2_(NO)­(SO_3_)]­PF_6_ (UCNP-RuNO)

The photorelease of the nitrosyl group from UCNP-RuNO was carried out using near-infrared (NIR) radiation. UCNP-RuNO was evaluated both in the solid state and as an aqueous suspension in phosphate buffer (0.1 mol L^–1^, pH 7.4) at 298 K. For the dispersion experiments, a UCNP-RuNO stock suspension was prepared at 200 μg mL^–1^. An aliquot of 250 μL was diluted with 2.75 mL of phosphate buffer, resulting in a UCNP-RuNO concentration of ca. 16.7 μg mL^–1^ before addition of cPTIO. For NO-release measurements, 60 μL of a cPTIO stock solution (ca. 100 μg mL^–1^, 3.17 × 10^–4^ mol L^–1^) was added, yielding a final cPTIO concentration of 1.96 μg mL^–1^ (6.21 × 10^–6^ mol L^–1^). The samples were irradiated with a 980 nm diode laser operating at 1200 mW for up to 6 h, and UV–vis spectra were recorded at regular intervals. NIR radiation was delivered through an Ocean Optics P600-0.25-SR optical fiber (600 μm core diameter, NA = 0.22), with the fiber tip positioned ca. 3 cm above the sample and the irradiation directed vertically downward at normal incidence (∼90° relative to the sample surface). Based on the fiber numerical aperture and the source-to-sample distance, the illuminated area was geometrically estimated as 1.44 cm^2^, corresponding to a power density of ca. 0.83 W cm^–2^. For the solid-state experiment, the material was irradiated in the solid state and subsequently dispersed in aqueous medium for acquisition of its electronic absorption spectrum.

### 2.8 Quantification of NO in Solution

The quantification of the nitrosyl group photoreleased by *cis*-[Ru­(dc-bpy)_2_(NO)­(SO_3_)]^+^ or UCNP-RuNO was performed by monitoring the decreased of the absorbance at 560 nm related to the amount of cPTIO remaining from the reaction of NO with this specific scavenger. A calibration curve of the absorbance at 560 nm and the concentration of cPTIO (in μg mL^–1^): 200, 180, 160, 140, 120, 100, 80, 60, 40, 20, 10, 6, and 2 was prepared to quantify cPTIO from measured absorbance.

### 2.9 Cell Viability Assay

The cytotoxicity of the samples was ascertained by using healthy epithelial cells (VERO). These cells were cultured in RPMI-1640 medium supplemented with 10% fetal bovine serum (FBS) and 1% penicillin/streptomycin, maintained in an incubator at 37 °C with 5% CO_2_. For the assays, cell suspensions were seeded into 96-well plates (1 × 10^4^ cells/well) and treated with samples containing different concentrations (ranging from 100 to 1.5 μg mL^–1^) of the NaYF_4_:Yb,Tm coated with PVP (UCNP), ZIF-8, *cis*-[Ru­(dc-bpy)_2_(SO_3_)­(NO)]^+^ (RuNO), NaYF_4_:Yb,Tm@ZIF-8-NH_2_ (UCNP@ZIF-8-NH_2_), and NaYF_4_:Yb,Tm@ZIF-8-NH-[Ru­(dc-bpy)_2_(SO_3_)­(NO)]^+^ (UCNP@ZIF-8/NH-RuNO) for 24 h. Afterward, 10 μL of MTT (3-(4,5-dimethylthiazol-2-yl)-2,5-diphenyltetrazolium bromide) diluted in PBS (5 mg mL^–1^) was added to each well and incubated for 3 h in an incubator at 37 °C with 5% CO_2_. Then, 100 μL of solvent was added to dissolve the formed formazan crystals. The absorbance at 570 nm of each well was recorded using a Thermo Scientific microplate reader and recorded. Cell viability was expressed as a percentage relative to the concentrations of the samples, with the negative control (cells treated only with the sample solubilizing solvent, namely, DMSO 10%) considered as 100% viability. All assays were performed in triplicate, and the obtained data were analyzed using the ANOVA statistical test for comparison between groups, with differences evaluated by the one-way post-test (*p* < 0.05).

### 2.10 Computational Studies

The structures of the compound were calculated in the singlet spin multiplicity without any constraints, and subsequent vibrational analyses were performed at the B3LYP/def2-TZVP level^48–50^ and the r^2^SCAN-3c^51^ composite method. The electronic spectrum was obtained using time-dependent DFT (TDDFT) calculations^52^ with CAM-B3LYP,^53^ wB97X-D3,^54^ and LC-PBE^55^ functionals and def2-TZVP^49,50^ basis set. The solvent effects in water were simulated by the conductor-like polarized continuous solvation model (C-PCM).^56,57^ The electronic transitions were analyzed based on oscillator strength, orbital composition, and natural transition orbitals.^58^ All calculations were performed using the Gaussian09 ^59^ and Orca 5.1 programs.^60^

## 3 Results and Discussion

### 3.1 Synthesis and Characterization of *cis*-[Ru­(dc-bpy)_2_(NO)­(SO_3_)]­PF_6_

The *cis*-[Ru­(dc-bpy)_2_(NO)­(SO_3_)]­PF_6_ compound was successfully synthesized in good yield (95% with respect to *cis*-[Ru­(dc-bpy)_2_(Cl)_2_] precursor) and characterized by FTIR, electronic spectroscopy, and cyclic voltammetry. The FTIR technique was employed to identify the peak corresponding to the nitrosyl group coordinated to the metal center. This ligand was characterized by the presence of a band at 1940 cm^–1^, assigned to the stretching of NO^+^, which agrees with the literature.^61^ It has been reported that nitrosyl can exhibit a stretching frequency in the range of 1923–1970 cm^–1^ for *cis*-[Ru­(bpy)_2_(NO)­L] complexes, with L = N_3_^–^, Cl^–^, NO_2_^–^, NH_3_, py, CH_3_CN.^61^ However, Silva and co-workers reported an analogous metal complex, namely, *cis*-[Ru­(bpy)_2_(NO)­(SO_3_)]^+^ for which they proposed that a peak at 1911 cm^–1^ was associated with the NO^+^ stretching mode.^47^ This large shift in the stretching mode of NO^+^ from 1911 to 1940 cm^–1^ in these complexes can be justified by the decrease of the π backbonding interaction from the metal to NO, caused by the trans dc-bpy ligand to NO^+^ compared to the bpy one.^47^

A computational modeling of *cis*-[Ru­(dc-bpy)_2_(NO)­(SO_3_)]^+^ complex was carried out at B3LYP/def2-TZVP and r^2^SCAN-3c levels. These methods were selected from an assessment of DFT functionals and basis sets to reproduce the vibrational frequencies (Tables S5 and S7) and structural features (Tables S6 and S8).^62,63^ Both methods provided very similar structures for the *cis*-[Ru­(dc-bpy)_2_(NO)­(SO_3_)]^+^ complex, which can be described as slightly distorted octahedral coordination geometry (Figure S4). The first method yielded the following distances and angles involving the nitrosyl ligand: Ru–NO = 1.738 Å, N–O = 1.140 Å, Ru–N–O = 176.5°, while with the second method: Ru–NO = 1.736 Å, N–O = 1.145 Å, Ru–N–O = 175.5°, which agree with the experimental data for the similar complexes (Table S8). Both methods provided a very good description of the vibrational spectrum of the *cis*-[Ru­(dc-bpy)_2_(NO)­(SO_3_)]^+^ complex (Figure S5) and were used to perform the assignments of the main features of the experimental FTIR spectrum (Table S7 and Figure S5). The stretching mode of ν­(NO^+^) was calculated at 1937 and 1933 cm^–1^ with B3LYP and r^2^SCAN-3c (Table S7), which agree with the observed value (1940 cm^–1^). It should be noted that this modeling considered the dc-bpy ligands neutral to be consistent with the degree of deprotonation in the solid state. However, in solution the carboxylic groups (R–COOH) could be deprotonated depending on the pH. For instance, the calculated stretching frequency of the NO ligand decreases 10 cm^–1^ when the carboxylic acid in the *cis*-dc-bpy is deprotonated. However, this frequency significantly increases by 65 cm^–1^ upon additional deprotonation of this *cis*-dc-bpy ligand. These results warrant additional computational and experimental investigations of this complex regarding its ability to release NO under different pH.

The absorption UV–vis spectra of *cis*-[Ru­(dc-bpy)_2_(SO_3_)­(NO)]^+^ and *cis*-[Ru­(dc-bpy)_2_(Cl)_2_] complexes were measured in water (Figures 2a and S3 and Tables S3 and S4). In both spectra, strong absorption bands were observed at shorter wavelengths (<315 nm); however, the intensity in the *cis*-[Ru­(dc-bpy)_2_(SO_3_)­(NO)]^+^ spectrum is much higher than in *cis*-[Ru­(dc-bpy)_2_(Cl)_2_], suggesting that additional transitions are present in the former complex. The band with maximum at 301 nm (ε: 1.72 × 10^4^ L mol^–1^ cm^–1^) is slightly asymmetric with a shoulder at 340 nm in the spectrum of *cis*-[Ru­(dc-bpy)_2_(SO_3_)­(NO)]^+^. This band (<315 nm) was also present in the spectrum of *cis*-[Ru­(dc-bpy)_2_(Cl)_2_], so it is assigned to intraligand π* ← π­(dc-bpy) transitions, typically observed in metal complexes containing a bipyridine ligand.^48^ The shoulder observed in the spectrum of *cis*-[Ru­(dc-bpy)_2_(SO_3_)­(NO)]^+^ can be attributed to the loss of symmetry of the dc-bpy ligands compared to *cis*-[Ru­(dc-bpy)_2_(Cl)_2_]. For *cis*-[Ru­(dc-bpy)_2_(SO_3_)­(NO)]^+^, two bands were observed at 429 nm (ε: 2.87 × 10^3^ L mol^–1^ cm^–1^) and 512 nm (ε: 1.09 × 10^3^ L mol^–1^ cm^–1^), whereas two bands at 385 and 531 nm were observed for *cis*-[Ru­(dc-bpy)_2_(Cl)_2_]. This suggests that the band at 429 nm could be assigned to a π*­(dc-bpy) ← dπ­(Ru^2+^) metal-to-ligand charge transfer (MLCT) transition, where the wavelength shift with respect to *cis*-[Ru­(dc-bpy)_2_(Cl)_2_] could be due to the backbonding effects of the NO ligand. Notice that for *cis*-[Ru­(bpy)_2_(SO_3_)­(NO)]^+^, a band at 340 nm (ε: 8.27 × 10^3^ L mol^–1^ cm^–1^) was attributed to this π*­(bpy) ← dπ­(Ru^2+^) MLCT transition,^47^ which emphasizes the strong effects of the carboxylic acid groups on the electronic structure of the dc-bpy ligands. The band at 530 nm was not observed in the spectrum of *cis*-[Ru­(bpy)_2_(SO_3_)­(NO)]^+^, which suggests that the band might be assigned to a π*­(dc-bpy) ← dπ­(Ru^2+^) MLCT transition associated with other dc-bpy ligand trans relative to NO. Alternatively, it could be attributed to (NO^+^)­π* ← dπ­(Ru^2+^) MLCT transition, which is known to occur in the 420–480 nm range for [Ru­(NH_3_)_4_(NO)­X]^n+^ complexes (X = OH^–^, Cl^–^, Br^–^, and I^–^), but they are very weak (ε < 50 L mol^–1^ cm^–1^),^61^ most likely due to the symmetry of these complexes. Given these possibilities, the absorption spectrum of the target *cis*-[Ru­(dc-bpy)_2_(SO_3_)­(NO)]^+^ complex was modeled by TDDFT quantum chemical calculations employing three long-range-corrected functionals that are known to describe charge transfer transitions adequately.^64,65^

**Figure 2:**
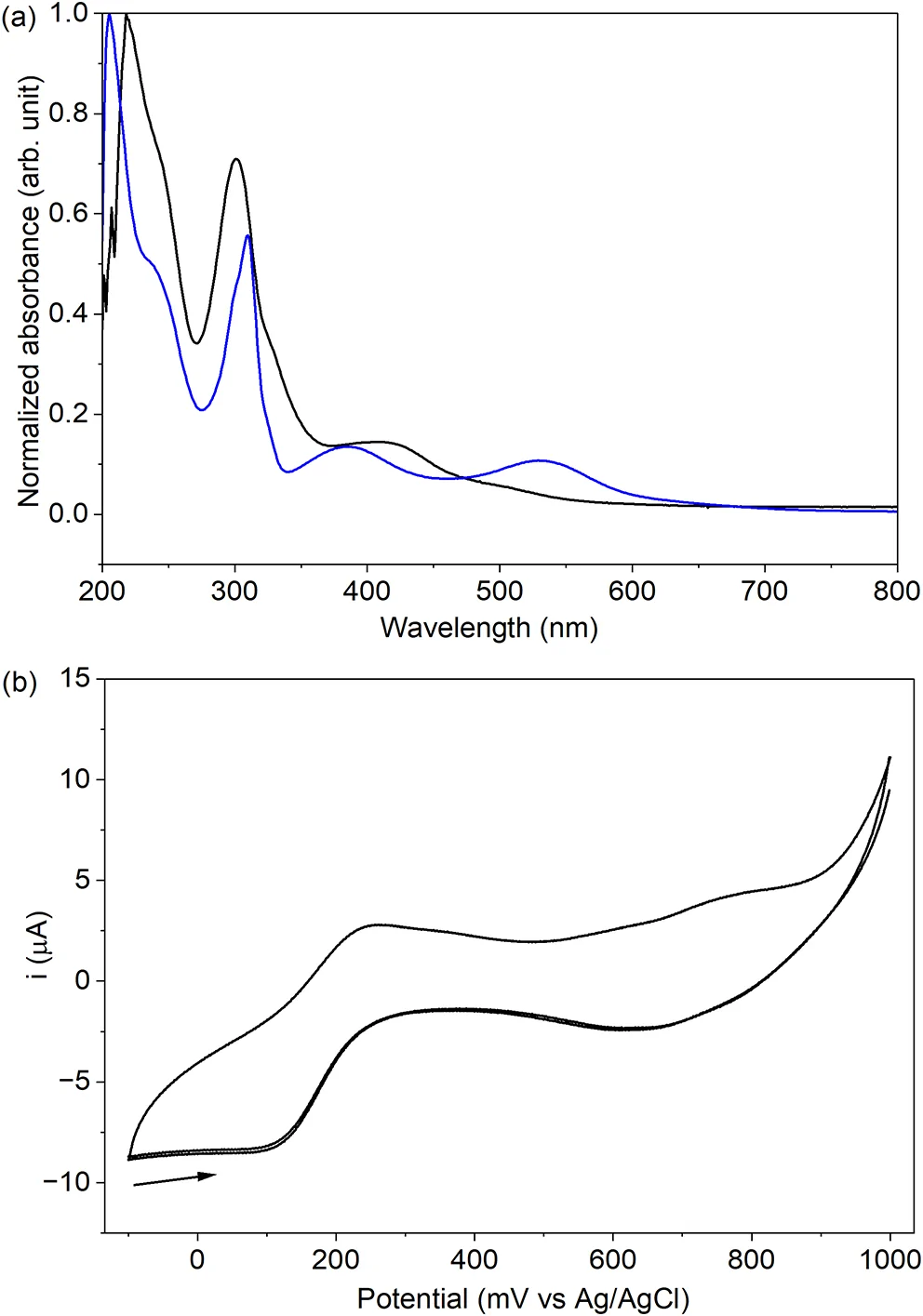
(a) UV–vis absorption spectrum in aqueous solution of *cis*-[Ru­(dc-bpy)_2_(Cl)_2_] (2.2 × 10^–5^ mol L^–1^, black line) and of *cis*-[Ru­(dc-bpy)_2_(SO_3_)­(NO)]^+^ (1.1 × 10^–5^ mol L^–1^, blue line), and (b) cyclic voltammogram of *cis*-[Ru­(dc-bpy)_2_(SO_3_)­(NO)]­PF_6_, in 0.1 mol L^–1^ HTFA solution with scan rate of 100 mV s^–1^.

Indeed, the electronic transitions and oscillator strengths were calculated by TDDFT method with different functionals for the isolated (gas phase) *cis*-[Ru­(dc-bpy)_2_(SO_3_)­(NO)]^+^ complex and in water (C-PCM solvation model) (Tables S9 and S10). The best agreement with the experimental absorption spectrum was obtained with the CAM-B3LYP functional and def2-SVP basis set (Figure S6 and Table S10), while increasing the basis set to def2-TZVP caused a systematic decrease of the calculated wavelengths and worsened the agreement (Table S9). Whereas, the other functionals overestimated the transition energies, but they presented the same trends in the peak positions and relative intensities. The inclusion of the solvent effects, by the C-PCM approach, caused a systematic decrease of the wavelengths, being largest for the MLCT transitions, e.g., 80 and 65 nm for π*­(NO) ← dπ­(Ru^2+^), and 53 nm for π*­(dc-bpy) ← dπ­(Ru^2+^), while the intraligand transitions were shifted by 6–11 nm (Table S10). Using the contributions of group molecular orbitals in each transition as well as the natural transition orbitals (NTOs) analysis (Figures S7–S9 and Table S10) the bands in the UV–vis spectrum were assigned. For instance, the band at 301 nm can be attributed to intraligand π* ← π­(dc-bpy) transitions, which corroborate the assignment proposed above and in the literature.^47^ The very intense bands at shorter wavelengths (<301 nm) were assigned mainly to interligand π*­(NO) ← π­(dc-bpy) transitions with smaller contributions from intraligand transitions, which is consistent with their much lower intensities in the spectrum of *cis*-[Ru­(dc-bpy)_2_(Cl)_2_] complex without NO ligand. For *cis*-[Ru­(dc-bpy)_2_(SO_3_)­(NO)]^+^, band at 429 nm was assigned to a MLCT π*­(dc-bpy) ← dπ­(Ru^2+^) transition, while at 512 nm could be attributed to a MLCT π*­(NO) ← dπ­(Ru^2+^) (Table S10). These assignments are consistent with the calculated large shifts due to the solvent effects as discussed above. An additional band corresponding to a MLCT π*­(NO) ← dπ­(Ru^2+^) transition was calculated (Table S10) in an energy range that is likely to be overlapped by the intense band at 340 nm.

Redox (oxidation–reduction) processes of the *cis*-[Ru­(dc-bpy)_2_(SO_3_)­(NO)]^+^ complex were investigated by cyclic voltammetry. Two electrochemical processes were observed by scanning the potential from +100 to 1000 mV (Figure 2b). The first process, described by eq 2 is related to the reduction of the nitrosyl group (NO^+/0^), which occurred at a potential of +105 mV vs Ag|AgCl, while a second process was observed at +630 mV assigned to the Ru^3+/2+^ redox process. This second process is associated with a chemical reaction of dissociation of NO^0^ as described by eqs 3 and 4, which is relevant to the NO release process and are described by the following sequence of chemical and electrochemical reactions (with the corresponding potential in mV)

2 $${\left[{\mathrm{Ru}}^{2+}{\left(\mathrm{dc}‐\mathrm{bpy}\right)}_{2}\left({\mathrm{SO}}_{3}\right)\left({\mathrm{NO}}^{+}\right)\right]}^{+}+e^{-}=\left[{\mathrm{Ru}}^{2+}{\left(\mathrm{dc}‐\mathrm{bpy}\right)}_{2}\left({\mathrm{SO}}_{3}\right)\left({\mathrm{NO}}^{0}\right)\right]+105$$

3 $$\left[{\mathrm{Ru}}^{2+}{\left(\mathrm{dc}‐\mathrm{bpy}\right)}_{2}\left({\mathrm{SO}}_{3}\right)\left({\mathrm{NO}}^{0}\right)\right]+H_{2}O=\left[{\mathrm{Ru}}^{2+}{\left(\mathrm{dc}‐\mathrm{bpy}\right)}_{2}\left({\mathrm{SO}}_{3}\right)\left(H_{2}O\right)\right]+\mathrm{NO}K_{\mathrm{NO}}$$

4 $$\left[{\mathrm{Ru}}^{2+}{\left(\mathrm{dc}‐\mathrm{bpy}\right)}_{2}\left({\mathrm{SO}}_{3}\right)\left(H_{2}O\right)\right]={\left[{\mathrm{Ru}}^{3+}{\left(\mathrm{dc}‐\mathrm{bpy}\right)}_{2}\left({\mathrm{SO}}_{3}\right)\left(H_{2}O\right)\right]}^{+}+e^{-}+630$$

Approximate linear relationships between the stretching wavenumber of the NO ligand, ν­(NO^+^), and the nitrosyl redox potential, *E*_1/2_(NO^+/0^), were established.^47,61^ For ten *trans*-[Ru­(NO)­(NH_3_)_4_(L)]^n+^ complexes, the slope of *E*_1/2_(NO^+/0^) vs ν­(NO^+^) was 10 ± 2 mV cm^–1^,^48^ while for three *cis*-[Ru­(bpy)_2_(NO)­(L)]^n+^ complexes and one *trans*-[Ru­(bpy)_2_(NO)­(SO_3_)]^+^ complex,^47^ an almost identical slope was obtained, namely, *E*_1/2_(NO^+/0^)/mV = −(18,676 ± 2483) + (9.7 ± 1.3) ν­(NO^+^) cm^–1^ (adjusted *R*-square = 0.95). Using ν­(NO^+^) = 1940 cm^–1^ for *cis*-[Ru­(dc-bpy)_2_(NO)­(SO_3_)]^+^, *E*_1/2_(NO^+/0^) can be predicted as 140 mV, which is in good agreement with the measured value, thus validating this relationship and the observed electrochemical data. In addition, these results quantify the effects of the carboxylic groups in the dc-bpy ligands by comparing to the bpy ones. For *cis*-[Ru­(bpy)_2_(NO)­(SO_3_)]^+^, ν­(NO^+^) = 1911 cm^–1^ and *E*_1/2_(NO^+/0^) = −140 mV,^47^ whereas for *cis*-[Ru­(dc-bpy)_2_(NO)­(SO_3_)]^+^, ν­(NO^+^) = 1940 cm^–1^ and *E*_1/2_(NO^+/0^) = +105 mV. Hence, the electron withdrawing effects of the carboxylic groups cause a decrease in the backdonation to π*­(NO^+^), which increases ν­(NO^+^) and *E*_1/2_(NO^+/0^) relative to bpy, leading to a less reactive NO^+^ group toward OH^–^.^66,67^

### 3.2 Reactivity of *cis*-[Ru­(dc-bpy)_2_(NO)­(SO_3_)]­PF_6_

The interconversion of nitrosyl to nitro ligand, Ru–NO^+^ ⇌ Ru–NO_2_^–^, is an important feature of NO metallodonors, which must be studied considering their potential biological application. In the literature, it is mentioned that when a change in pH occurs, new bands in the electronic spectrum are observed in the range of 400–450 nm. This behavior was reported for the metal complexes studied by Silva et al.^47^ and Borges et al.,^68^ among others.^69,70^ Figure S10 shows a pH-dependent profile of the electronic spectra of the nitrosyl complex, where an increase in pH causes an increase of the intensity of the band at 305 nm and the appearance of bands at 356 and 449 nm, while the bands at 329 and 414 nm decrease their intensities. Given the strong dependence of the band at 449 nm with the pH, its absorbance was selected to obtain the titration curve that was fitted to a sigmoidal model (Figure S11). The inflection point, i.e., the pH at which the main species for nitrosyl-nitro ligand interconversion are equimolar and the p*K*_NO_ can be extracted. Interestingly, this pH was observed at 9.07 (Figure S11), which is significantly high. However, it is still lower than what was reported for the analogue system [Ru­(bpy)_2_(SO_3_)­(NO)]^+^, but in agreement with all data collected. More importantly, this value demonstrates that at physiological pH, the nitrosyl character is dominant for the *cis*-[Ru­(dc-bpy)_2_(NO)­(SO_3_)]^+^ complex, which is relevant for NO release.

The release of NO upon blue light irradiation was qualitatively determined by using cPTIO (2-(4-carboxyphenyl)-4,4,5,5-tetramethylimidazoline-1-oxyl-3-oxide potassium salt). A solution with the nitrosyl complex and cPTIO was irradiated with blue light, and the UV–vis spectrum was recorded every 30 min, where a decrease of the absorbance at 560 nm was observed. No further change in the spectrum was noted after 2.5 h of irradiation (Figure S12). The cPTIO probe is described as reacting quickly and stoichiometrically with NO-originating cPTI, which has an absorption band at 440 nm and no absorption at 560 nm.^71^ Our results support a photorelease of NO as seen for its analogue system,^72^ and corroborate the work of Carvalho et al., where cPTIO was used for monitoring the photorelease of NO using *trans*-[Fe­(cyclam)­(NO)­Cl]^2+^ complex.^73^

### 3.3 Characterization of the NaYF_4_:Yb,Tm@ZIF-8-NH_2_ System

The NaYF_4_:Yb,Tm UCNPs were successfully synthesized and characterized by XDR, which showed the presence of two phases: hexagonal (β-NaYF_4_:Yb,Tm) and cubic (α-NaYF_4_:Yb,Tm), with a predominance of the hexagonal phase (Figure S13). Notice that the presence of two phases did not hinder the development of the next steps. These UCNPs were coated by ZIF-8, which was then functionalized to present amine (R-NH_2_) groups on its surface. Indeed, these steps were corroborated by PXRD and FTIR analyses. The crystalline pattern of ZIF-8 was maintained in the PXRD and new peaks were observed at 2θ: 17.08, 29.82, 30.71, and 43.35°, which are assigned to UCNP in the system NaYF_4_:Yb,Tm@ZIF-8-NH_2_ (Figure S14 and Table S11). These results agree with data reported in the literature.^74,75^

Aiming at investigating the functionalization of these UCNP@ZIF-8’s, FTIR and UV–vis spectra were obtained. Vibrational analysis demonstrated that after functionalization, a sharp peak appeared at 2975 cm^–1^, which can be attributed to C–H stretches of ethylenediamine (EDA), and a broad peak 3600–3750 cm^–1^ characteristic of N–H stretching of EDA (Figure S15 and Table S12). The electronic absorption spectrum of this sample showed an intense absorption within 300–500 nm range (Figure S16) due to the functionalization. All of these data agree with the literature, thus supporting the preparation of this material.^76^

The upconversion emission spectra of NaYF_4_:Yb,Tm, NaYF_4_:Yb,Tm@ZIF-8, and NaYF_4_:Yb,Tm@ZIF-8-NH_2_ NPs were recorded upon excitation at 980 nm with a diode laser. All of these materials obtained presented four emission bands (Figure S17) due to transitions in the thulium ion (Tm^3+^), namely, the ^1^I_6_ → ^3^F_4_ and ^1^D_2_ → ^3^H_6_ transitions refer to the emission maximum at 345 and 361 nm, respectively, in the near-UV region, while the emissions at 451 and 475 nm, in the blue region, were assigned to the ^1^D_2_ → ^3^F_4_ and ^1^G_4_ → ^3^H_6_ transitions, respectively. The mechanisms and pathways for these upconversion emissions involving the Yb^3+^ and Tm^3+^ pair have been investigated and discussed thoroughly in the literature and studies carried out by Karami et al.^74^ for ZnO-coated β-NaYF_4_:Yb,Tm found similar results, which corroborate the preparation of these materials.

Morphological analysis and average size of the nanocrystals were carried out using SEM and TEM. The SEM images (Figure S18) of ZIF-8 and NaYF_4_:Yb,Tm@ZIF-8 showed these materials as having an orthorhombic dodecahedron morphology,^77^ while the functionalized NaYF_4_:Yb,Tm@ZIF-8-NH_2_ composite showed a different morphology, probably due to the solvothermal synthesis process.^75^ TEM images in conjunction with EDX (Figure S19) provide evidence of the coating of the UCNPs by ZIF-8, which is consistent with the increase in the size of the particles observed in the SEM (Figure S18). Namely, pristine ZIF-8 had an average size of 68.30 ± 6.47 nm, which became 103 ± 5.65 nm after coating the UCNPs and 192.30 ± 11.37 nm after functionalization of the coated composite (Figure S8). By preparing a core–shell system with the core being NaYF_4_:Yb,Tm and the shell being ZIF-8 or ZIF-8-NH_2_, it is expected to increase in size.

### 3.4 Characterization of NaYF_4_:Yb,Tm@ZIF-8-NH-[Ru­(dc-bpy)_2_(SO_3_)­(NO)]­PF_6_ (UCNP-RuNO)

The FTIR technique was employed to investigate the spectral changes associated with the incorporation of the Ru^2+^ coordination compound into the nanocomposite. In the infrared spectrum of the NaYF_4_:Yb,Tm@ZIF-8-NH-[Ru­(dc-bpy)_2_(SO_3_)­(NO)]­PF_6_ system, denoted as UCNP-RuNO, the ν­(NO^+^) band shifted to a lower frequency, from approximately 1940 cm^–1^ in the free complex to 1909 cm^–1^ after incorporation into the material (Figure 3a). This shift indicates a modification of the electronic environment of the RuNO complex and may be related to changes in the electronic properties of the ligand trans to the nitrosyl group, as discussed above and previously reported by Silva et al.^47^ In addition, the disappearance of the band associated with the carboxylic acid ν­(C=O) vibration, together with the disappearance of the ν­(N–H) bands attributed to the amino groups of the functionalized material, indicates a modification of these functionalities after incorporation of the complex. In the UCNP-RuNO spectrum, bands at 1618 and 1546 cm^–1^ are observed in regions consistent with amide I, mainly associated with ν­(C=O), and amide II, mainly associated with δ­(N–H) and ν­(C–N), respectively. These bands may also contain contributions from the dc-bpy and imidazolate ring vibrations; therefore, they are interpreted as being consistent with, rather than exclusively diagnostic of, amide bond formation. These spectral changes (see Table S13 for detailed peak assignments) support the proposed covalent anchoring of the Ru complex to the amino-functionalized ZIF-8 framework via amide bond formation.

**Figure 3:**
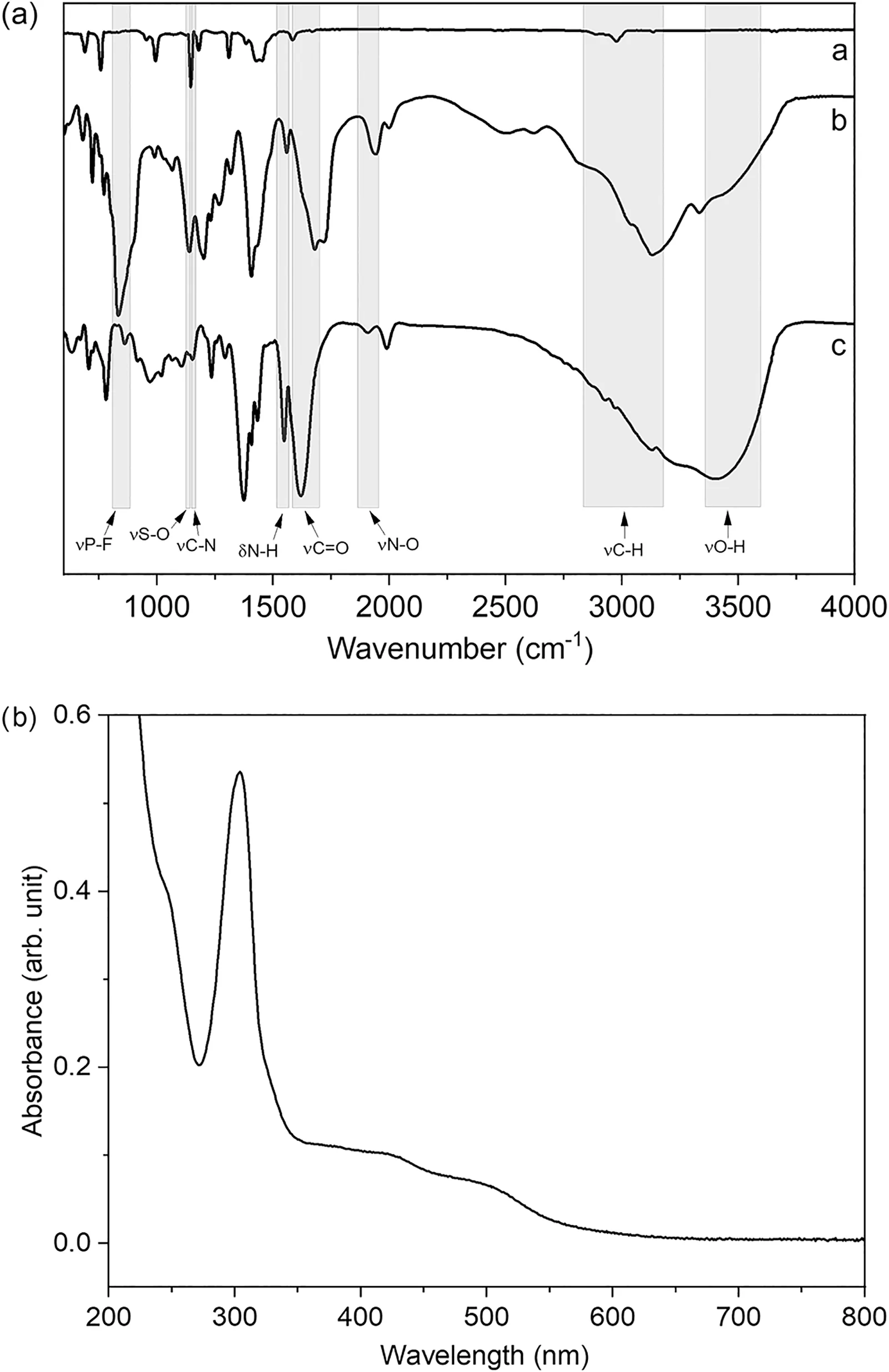
(a) FTIR spectra of NaYF_4_:Yb,Tm@ZIF-8-NH_2_ (denoted as a), *cis*-[Ru­(dc-bpy)_2_(SO_3_)­(NO)]­PF_6_ (denoted as b, RuNO), and NaYF_4_:Yb,Tm@ZIF-8-NH-[Ru­(dc-bpy)_2_(SO_3_)­(NO)]­PF_6_ (denoted as c, UCNP-RuNO), recorded using KBr pellets and showing the main vibrational bands of the functional groups; and (b) UV–vis absorption spectrum of UCNP-RuNO in an aqueous suspension (50 ppm). Water was used as the reference for baseline correction (blank). No additional mathematical corrections were applied. The suspension remained well dispersed and visually stable during the measurement, with no visible precipitation.

The electronic behavior of this system was evaluated by UV–vis absorption spectroscopy using an aqueous dispersion (50 ppm), which was prepared by ultrasonication at room temperature prior to measurement. The spectrum exhibited four main absorption bands with maxima at 303, 335, 423, and 505 nm (Figure 3b). The first two contributions are in the UV region, whereas the bands centered at approximately 423 and 505 nm occur in the violet–blue and blue–green regions, respectively. These spectral features are consistent with the absorption profile of the RuNO complex, indicating that the electronic transitions of the complex are preserved after incorporation into the nanocomposite.^77^ Minor shifts and changes in the relative intensities of these bands may be attributed to the amide bond formation of the carboxylic acid the dc-bpy ligand.

The surface chemical composition of the UCNP-RuNO composite was investigated by XPS. The survey spectrum (Figure 4a) confirmed the presence of the main elements associated with the UCNPs, the functionalized ZIF-8, and the ruthenium complex, namely, Na, F, Y, Yb, Tm, Zn, N, C, O, S, and Ru.

**Figure 4:**
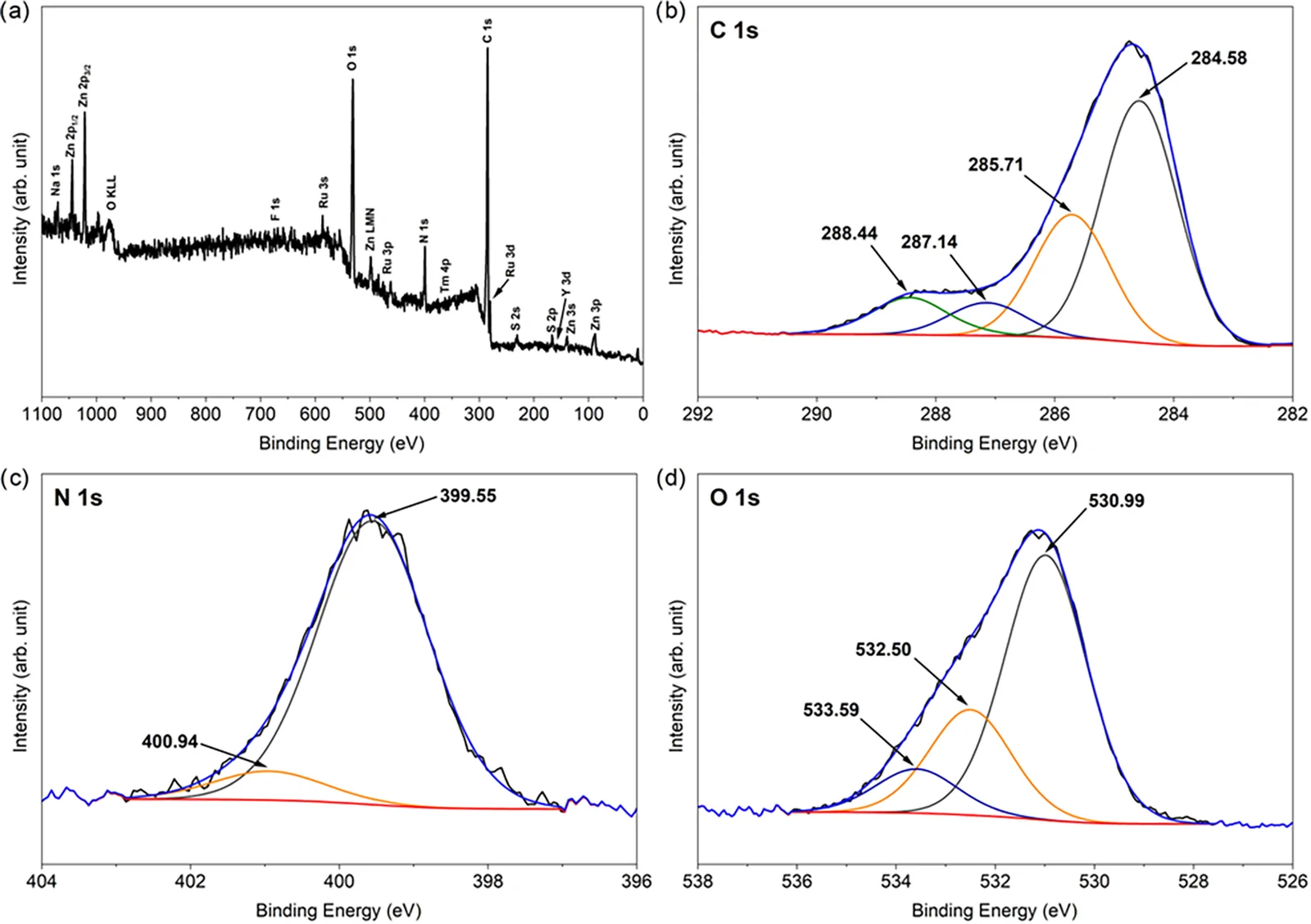
XPS spectra of the UCNP-RuNO material: (a) survey spectrum; high-resolution spectrum with deconvoluted components of (b) C 1s; (c) N 1s; and (d) O 1s. In panels (b–d), the experimental spectrum is shown in black, the fitted envelope in blue, and the baseline in red, together with the individual deconvoluted components.

The high-resolution C 1s (Figure 4b) region was deconvoluted into four components at 284.58, 285.71, 287.14, and 288.44 eV. It should also be noted that the Ru 3d_3/2_ contribution partially overlaps with the C 1s region around 284–285 eV and may contribute to the intensity observed in this spectral range. The component at 284.58 eV is mainly assigned to C–C, C–H, and C=C chemical environments, including aromatic carbons. The contribution at 285.71 eV is consistent with carbons bonded to nitrogen or oxygen, i.e., C–N and C–O, present in the imidazolate and bipyridyl groups. The component at 287.14 eV is assigned to C=O and C=N environments, whereas the higher-binding-energy contribution at 288.44 eV is consistent with O–C=O and O=C–N environments and may contain contributions from both remaining carboxylic groups and amide carbonyl carbon. The high-resolution N 1s (Figure 4c) region exhibited two components at 399.55 and 400.94 eV, with the former being assigned to N–C, N=C, C–NH*_x_* environments, including contributions from the imidazolate groups of ZIF-8, the bipyridyl rings, and residual amino groups. The component at 400.94 eV is consistent with nitrogen in N–C=O moieties attributed to amide groups. The high-resolution O 1s (Figure 4d) region was deconvoluted into three components at 530.99, 532.50, and 533.59 eV, with the former being assigned to oxygen atoms in the sulfite group ligand. The component at 532.50 eV is consistent with carbonyl oxygen environments, including contributions from O=C and O=C–N, and may contain contributions from amide and carboxylic groups present in the dc-bpy ligand. The contribution at 533.59 eV is assigned to O–C and O–H environments and adsorbed water on the surface.

These results, particularly the components at 288.44, 400.94, and 532.50 eV, are consistent and corroborate the FTIR assignments (bands at 1618 and 1546 cm^–1^) regarding the amide bond formed between the amino group of functionalized ZIF-8 and carboxylic group of dc-bpy ligand in the RuNO complex.

The upconversion behavior of the UCNPs (NaYF_4_:Yb,Tm) and of the core–shell systems (UCNP@ZIF-8 and UCNP@ZIF-8-NH_2_) were investigated and their emission spectra have the same bands and relative intensities. It is noteworthy that the UC emission intensities of all bands in the UCNP-ZIF-8 and UCNP-ZIF-8-NH_2_ systems decreased compared to UCNPs (Figure S17a,b). This systematic decrease can be ascribed to the decrease of the Yb/Tm concentrations due to an increase in particle size of the core–shell systems (Figure S18) and other effects such as inner-filter and quenching by more nonradiative deactivation and surface defects in the core–shell formation. However, the emission intensities of the conjugated system UCNP-RuNO (Figure S17c) are several orders of magnitude smaller than the core–shell systems without the anchored RuNO complex, particularly in the blue-region of the spectrum. A plausible explanation for these observations by considering that the RuNO complexes partially absorb the photons emitted in the conversion process, namely, a radiative mechanism. Alternatively, a nonradiative energy migration from the UCNP to the RuNO complex could be invoked. Indeed, nonradiative interfacial and interparticle energy migration in core–shell nanostructures have been observed^78–81^ when (quasi) resonant states are available to mediate a succession of short-ranger energy transfers.^82,83^ However, because the distances between sensitizer (UCNP) and the activator (RuNO) are very large (>10 nm, Figure S19) and there are no mediating quasi-resonant states (ZIF-8), this nonradiative activation of the optical centers of the RuNO complexes can be disregarded. Indeed, a more detailed assessment of the NIR photoactivation of the RuNO complex would require additional investigations employing sophisticated spectroscopic techniques (e.g., time-resolved luminescence) and the synthesis of new composites with inert optical centers (e.g., Zn^II^NO complex), which is beyond the scope of the present work.

### 3.5 Photorelease of NO by NaYF_4_:Yb,Tm@ZIF-8-NH-[Ru­(dc-bpy)_2_(SO_3_)­(NO)]­PF_6_ (UCNP-RuNO)

As shown above, the release of nitric oxide from the nitrosyl Ru^2+^ complex can occur upon blue light irradiation, and the visible radiation emitted by UCNP-RuNO, upon NIR irradiation, is strongly absorbed. These are relevant for the potential of UCNP-RuNO for the controlled release of NO by irradiation.

The photorelease of the nitrosyl group by UCNP-RuNO was carried out in solid state and in aqueous suspension (25 mg mL^–1^, phosphate buffer, 0.1 mol L^–1^, pH 7.4) by irradiation with a diode laser at 980 nm (near-infrared) and controlled temperature 298 K. The photorelease was investigated qualitatively by monitoring the changes in the UV–vis spectrum during the irradiation. In the solid state, the system was irradiated up to 6 h, then the irradiated solid was suspended and analyzed. For the aqueous suspension, a solution containing cPTIO was added to quantify the amount of NO released. In addition, the temperature profile of the suspension (and of pure solvent) during irradiation was monitored by a FLIR Ex series thermographic camera.

The release of nitric oxide by UCNP-RuNO via upconversion was investigated by irradiating a solid and aqueous suspension of UCNP-RuNO with a near-infrared laser. Indeed, the UV–vis spectrum upon NIR irradiation showed significant changes in the absorption bands. Noteworthy is the disappearance of the band at 512 nm, and the appearance of two new bands at 362 and 473 nm (Figure 5a). This is evidence for the release of nitric oxide by UCNP-RuNO because the band at 512 nm was assigned to MLCT π*­(NO) ← dπ­(Ru^2+^) transition, which would disappear upon the release of the NO ligand. The appearance of those new bands is probably due to the formation of an aquo-ruthenium complex after the release of NO.

**Figure 5:**
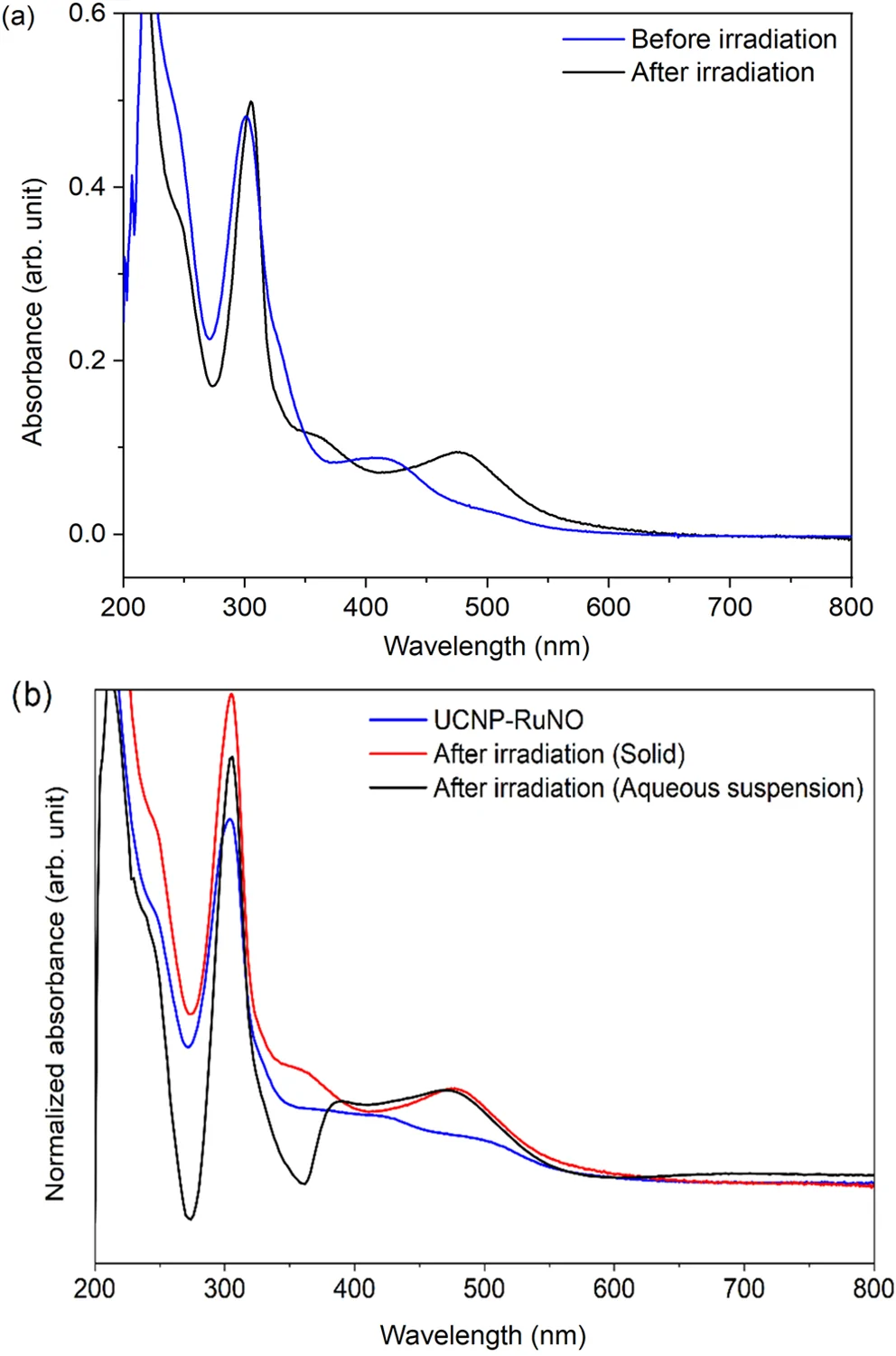
(a) UV–vis absorption spectra of NaYF_4_:Yb/Tm@ZIF-8-NH-[Ru­(dc-bpy)_2_(SO_3_)­(NO)]­PF_6_ (UCNP-RuNO) before (blue line) and after (black line) irradiation with a laser at 980 nm during 6 h of a phosphate buffer suspension (0.1 mol L^–1^, pH 7.4, 25 °C). (b) Normalized UV–vis absorption spectra of UCNP-RuNO as prepared (blue line), and after irradiation in solid state (red line) and in aqueous suspension (black line) with a NIR diode laser during 6 h.

The amount of NO released by NIR irradiation of UCNP-RuNO in dispersion was quantified by its reaction with cPTIO. A decrease of the absorbance at 560 nm, related to the band of cPTIO, was observed after irradiation of UCNP-RuNO, indicating the consumption cPTIO, because the NO released in the medium reacted with this scavenger. Thus, UCNP-RuNO has the capacity to release nitric oxide when irradiated with the 980 nm laser. A dispersion without cPTIO was used as reference (Figure S20) and the UV–vis spectrum at 0, 30, 60, 120, and 180 min of irradiation was recorded and the band at 560 nm was monitored. From a calibration curve, for the concentrations of cPTIO within 2–200 μg mL^–1^ (Figure S21), and the reading of the absorbance at 560 nm the concentration of cPTIO in solution was determined. The initial concentration of cPTIO was 1.98 μg mL^–1^, which during the first 30 min of irradiation decreased to 1.95 μg mL^–1^, and in the next periods of 60, 120, and 180 min of irradiation its concentration was 1.82, 1.78, and 1.66 μg mL^–1^, respectively. Considering a 1:1 stoichiometric reaction between cPTIO and NO, the decrease in cPTIO concentration was converted to a molar basis and attributed to the amount of NO released. Accordingly, the NO concentration increased to 0.095, 0.507, 0.634, and 1.015 μmol L^–1^ after 30, 60, 120, and 180 min of NIR irradiation, respectively. These values correspond to ca. 5.8, 31.0, 38.8, and 62.1 nmol of NO per mg of UCNP-RuNO, demonstrating a progressive increase in NO release with irradiation time.

After the NO-release experiment in the presence of cPTIO, the resulting solution was analyzed by UV–vis spectroscopy to evaluate the spectral changes associated with transformations of the RuNO complex upon NO release. For this analysis, a solution containing only cPTIO was used as reference (or blank), allowing for a control of the photorelease experiment. This spectrum was then compared with those recorded for the material before and after irradiation in the solid state (Figure 5b normalized and Figure S24 non-normalized.). A band at 473 nm was observed, whereas the contribution previously detected at 362 nm for the material irradiated in the solid state was not clearly observed. These results highlight two relevant aspects. First, the presence of the 473 nm band reproduces the main spectral feature observed after irradiation of the material in the solid state, supporting the formation of a modified Ru species following NO release. Second, the apparent absence of the contribution at 362 nm may be related to spectral overlap with an intense cPTIO absorption band in the same region and its consequent subtraction during acquisition when the scavenger was used as blank. Thus, comparison of these experiments indicates that similar spectral changes are produced following NO photorelease. The species formed after loss of the NO ligand may involve [Ru^II^–solvent] or [Ru^III^–solvent] species, as proposed by Rose and Mascharak^22^ and demonstrated for a structurally related complex by Cândido et al.^72^

In addition, the temperature profile was monitored to verify whether the dispersion was heated by the photophysical processes during NIR irradiation. The images obtained by with a thermal camera of the buffer aqueous with UCNP-RuNO (dispersion) and without UCNP-RuNO are depicted in Figure 6a–j, respectively, after 0, 30, 60, 120, and 180 min of irradiation with a diode laser at 980 nm. It was observed that, upon irradiation, the temperature increased from ca. 27 to 41–45 °C for both samples (with and without UCNP-RuNO) and almost identical profiles, suggesting that the local increase in temperature is not caused by processes involving the photorelease by UCNP-RuNO, but rather by the absorption of near-infrared energy by the aqueous solution. This is consistent with spectral behavior of water that indicates a strong absorption in NIR region, as discussed by Jensen et al.^84^ The highest local temperature achieved by the excitation source employed is 45 °C, which may potentially cause hyperthermic effects or tissue damage in organisms with a physiological temperature of approximately 37 °C; therefore, the exposure time may be a crucial factor in the treatment.^85^ In fact, this localized temperature increase could be combined synergically with the photorelease of NO as a strategy to improve the biological response desired.

**Figure 6:**
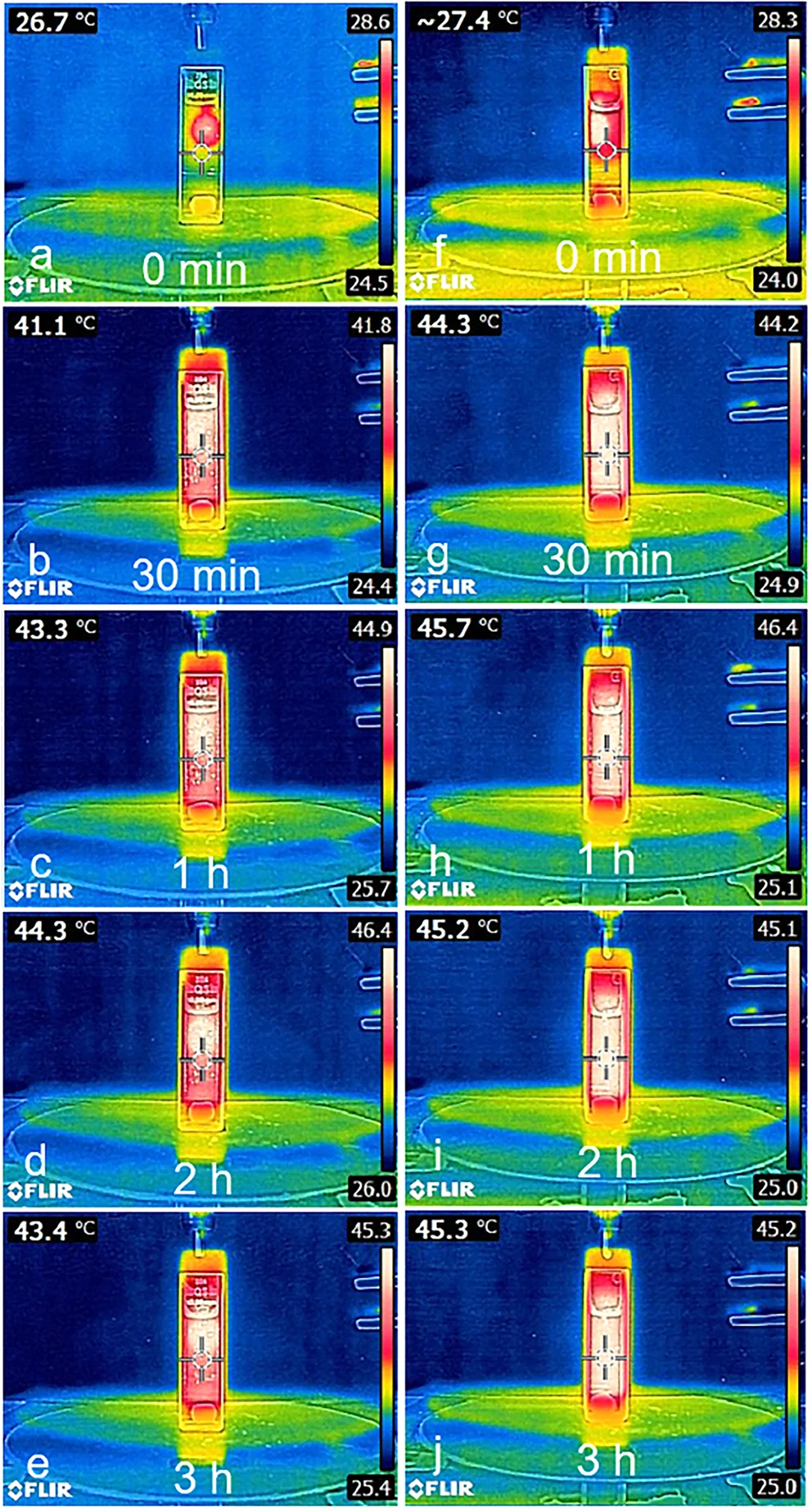
Thermographic images of phosphate buffer aqueous solution (0.1 mol L^–1^, pH 7.4, 25 °C) with NaYF_4_:Yb,Tm@ZIF-8-NH-[Ru­(dc-bpy)_2_(SO_3_)­(NO)]^+^ (UCNP-RuNO) dispersed (images on the left: a–e) and without UCNP-RuNO (images on the right: f–j) after 0, 30, 60, 120, and 180 min (top to bottom) of irradiation with a diode laser at 980 nm.

To evaluate the influence of temperature on the experiments previously described, control studies were performed (Figure S22), using the corresponding complex solution or material dispersion without cPTIO as reference (or blank) for spectral acquisition in the presence of the scavenger. Initially, the scavenger used to monitor NO release was exposed to the 980 nm laser under the same experimental conditions previously described (Figure S22a). No significant changes were observed in the absorption spectra between 5 and 60 min of irradiation, indicating that cPTIO remains stable under these conditions. When the RuNO complex was exposed either to 980 nm irradiation (Figure S22b) or to a thermal bath at 45 °C (Figure S22c) in the presence of cPTIO to evaluate its consumption, spectral changes were observed under both conditions, with the appearance of bands at approximately 306 and 473 nm. These changes support the formation of a new complex species following NO release, as previously demonstrated and discussed. However, the spectral changes observed for the free complex under 980 nm irradiation were more pronounced than those observed upon heating at 45 °C. This behavior corroborates the synergic photo and thermal release of NO because the significant absorption of aqueous solution irradiated at 980 nm causes its heating. This is consistent with previous reports on ruthenium nitrosyl complexes, including structurally related systems, in which NO release may occur spontaneously or be promoted by heating, as demonstrated by Silva et al.^47^ These same control experiments were performed for the NO release from the material, which considered the presence of the scavenger under NIR irradiation and in the dark (light-protected conditions), illustrated in Figure S22d, as well as upon heating at 45 °C (Figure S22e). In the dark, no significant changes were observed in the cPTIO spectrum between 5 and 60 min, indicating that no detectable spontaneous NO release occurs under these conditions. In a thermal bath at 45 °C, the material exhibited insignificant spectral changes, in contrast to those observed for the free complex, demonstrating a significant increase in the thermal stability for release of NO from the complex in the UCNP-RuNO system.

Additional control experiments were carried out with the RuNO complex and the UCNP-RuNO system in the absence of cPTIO, under 980 nm laser irradiation and heating at 45 °C, while maintaining the same experimental conditions (Figure S23). In the spectra of the RuNO complex obtained under NIR irradiation (Figure S23a) and upon heating at 45 °C (Figure S23b), spectral changes were observed that corroborate the results obtained in the presence of cPTIO. Notice that in the experiments performed with cPTIO, the corresponding complex solution in the absence of the scavenger was used as reference, thereby reducing the spectral contribution of the initial complex and making the bands associated with the species formed after NO release more evident. In contrast, in the spectra recorded directly for the complex in the absence of cPTIO, these changes are less pronounced because of the overlap between the bands of the initial RuNO complex and those of the new species formed after NO release. For the UCNP-RuNO system under 980 nm irradiation (Figure S23c), the spectral changes become more pronounced, with the appearance of contributions at 362 and 473 nm. In contrast, in the spectrum of UCNP-RuNO subjected only to heating at 45 °C (Figure S23d), the changes are considerably less pronounced than those observed for the irradiated material. These results corroborate the experiments performed in the presence of cPTIO and further support the increase of thermal stability for the release of NO from the complex anchored on ZIF-8, i.e., in the UCNP-RuNO system.

In combination with the results presented above, these experiments are consistent with an upconversion-mediated photoactivation mechanism. In this process, the UCNPs absorb the incident 980 nm radiation and convert it to higher-energy photons in the blue spectral region, which are absorbed by the RuNO complex, promoting its photoactivation and the subsequent release of NO. Indeed, these results further support a radiative emission-absorption mechanism discussed previously.

### 3.6 Cell Viability Study

The cytotoxic activity of the materials and complexes obtained was studied to assess their potential application in biological systems. The upconversion nanoparticles coated with PVP (NaYF_4_:Yb,Tm, Figure 7a) showed viability at all tested concentrations, with concentrations with 1.5–50 μg mL^–1^ stimulating cell proliferation with percentages of 122–108%, respectively. However, at a concentration of 100 μg mL^–1^, viability decreased to 80%. These results are consistent with those presented by Bastos et al.,^86^ which demonstrated that the surface coating of UCNPs can influence their viability. The tests conducted with ZIF-8 (Figure 7b) showed viability and stimulation of cell proliferation at all concentrations, which is expected given that ZIF-8 has low cytotoxic activity due to its chemical stability, as demonstrated by de Moura Ferraz et al.^87^

**Figure 7:**
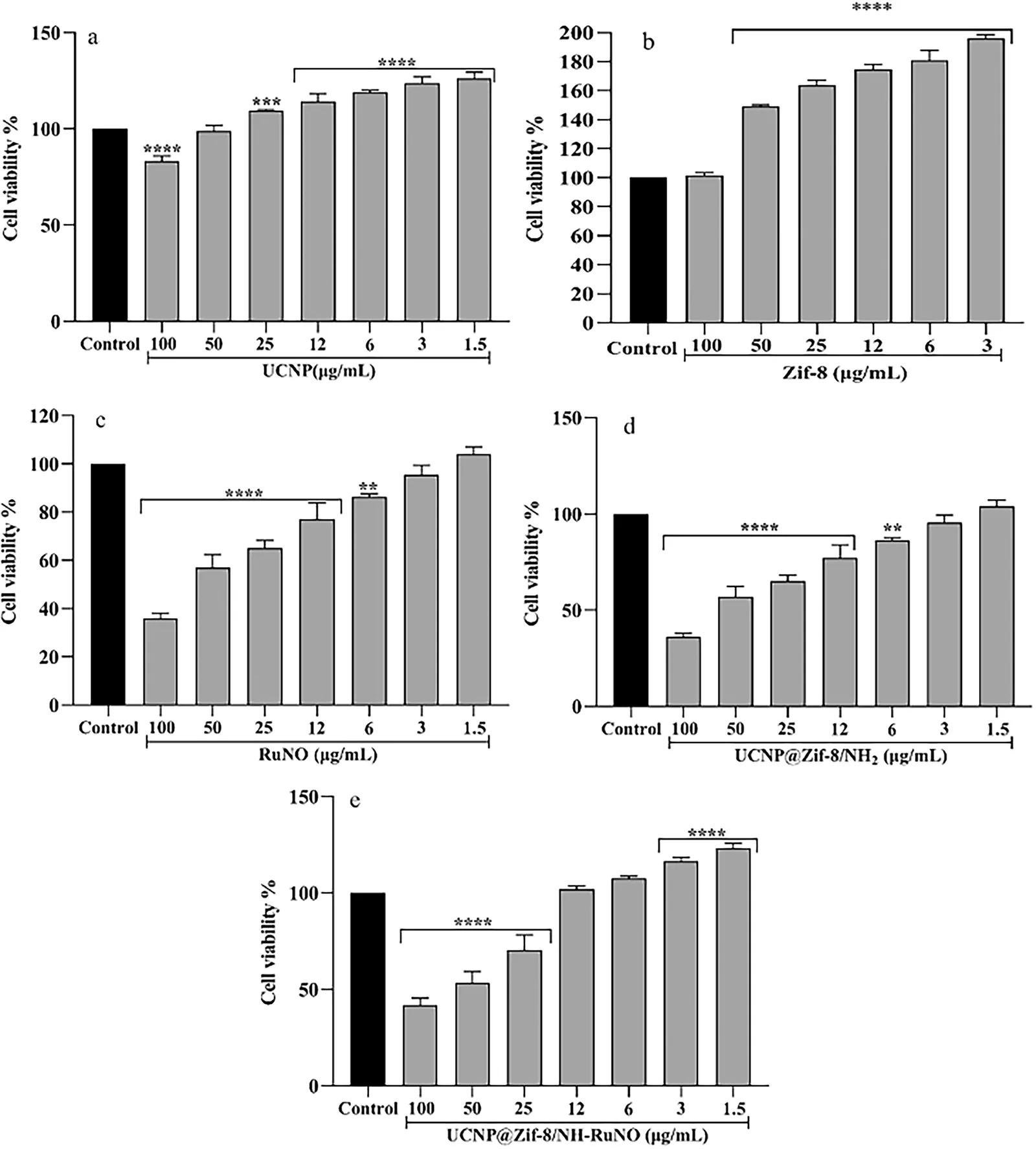
Viability assay in healthy epithelial cells (VERO) of the samples containing NaYF_4_:Yb,Tm coated with PVP (a), ZIF-8 (b), *cis*-[Ru­(dc-bpy)_2_(SO_3_)­(NO)]^+^ (c), NaYF_4_:Yb,Tm@ZIF-8-NH_2_ (d), and NaYF_4_:Yb,Tm@ZIF-8-NH-[Ru­(dc-bpy)_2_(SO_3_)­(NO)]^+^ (e) at different concentrations over a 24-h exposure period. Conducted using MTT assays followed by statistical treatment (*p* < 0.05).

The complex *cis*-[Ru­(dc-bpy)_2_(SO_3_)­(NO)]^+^ (Figure 7c) showed viability for concentrations within 1.5–6 μg mL^–1^ with percentages of 102–93%, respectively. At concentrations between 12 and 100 μg mL^–1^, there was a significant decrease in viability, indicating that these concentrations are not suitable for application in biological systems. Similar data were presented by Gaspari et al.^88^ for the complex [Ru^2+^(NO)­(pyLysO)­(bpy)_2_]­(PF_6_)_2_, which demonstrated cytotoxicity against healthy MCF-10A mammary cells. The NaYF_4_:Yb,Tm@ZIF-8-NH_2_ composite (Figure 7d) exhibited cell viability for concentrations of 1.5–6 μg mL^–1^, ranging from 100 to 86%, and showed a significant decrease in viability at higher concentrations. This result is intriguing given that both the UCNP and ZIF-8 did not exhibit high cytotoxicity. This synergistic effect may be explained by the modifications made to the ZIF-8 surface with the introduction of amino groups from ethylenediamine. These groups introduced for anchoring the metal complex could act as linkers to biomolecules, as demonstrated by Awasthi et al.,^89^ causing cell damage and, consequently, the death of healthy cells.

The performance of the NaYF_4_:Yb,Tm@ZIF-8-NH-[Ru­(dc-bpy)_2_(SO_3_)­(NO)]^+^ (UCNP-RuNO) system (Figure 7e) in healthy cells showed that after the metal complex was anchored on the surface of the NaYF_4_:Yb,Tm@ZIF-8-NH_2_ composite, the amino groups were passivated, leading to improved cell viability. For concentrations within 1.5–12 μg mL^–1^, cell viability percentages were observed to be 125–103%, indicating stimulation of cell proliferation. However, at concentrations of 25–100 μg mL^–1^, the system showed low viability with percentages of 77–41%.

Thus, the UCNP-RuNO system produced in this work for controlled nitric oxide release via upconversion can be used in biological systems at a concentration up to 12 μg mL^–1^, being safe due to its low cytotoxic effect at the tested times. Further studies are needed to verify the efficacy of UCNP-RuNO against radiation and its effects on healthy and cancerous cells, as well as in vivo testing.

## 4 Conclusions

The strategy of combining UCNPs (e.g., NaYF_4_:Yb,Tm) with Ru^II^-nitrosyl complexes (e.g., *cis*-[Ru­(dc-bpy)_2_(SO_3_)­(NO)]^+^) to produce a UCNP-RuNO composite (NaYF_4_:Yb,Tm@ZIF-8-NH-[Ru­(dc-bpy)_2_(SO_3_)­(NO)]^+^) capable of releasing NO upon near-infrared irradiation (980 nm) was successfully demonstrated. The upconversion nanoparticles were coated with ZIF-8, yielding a core–shell system, and subsequently functionalized with ethylenediamine to enable the covalent anchoring of the RuNO complex via amide bond between amino groups of functionalized ZIF-8-NH_2_ and carboxylic acid groups of the dc-bpy ligands. The combined FTIR and XPS results provided evidence for this structural assignment. In the absence of the Ru complex, the systems (UCNPs, UCNP-ZIF-8, and UCNP-ZIF-8-NH_2_) displayed efficient upconversion emission in the blue region (450 and 475 nm). After incorporation of the RuNO complex, a pronounced decrease in the UC emission intensity was observed. These and other results are consistent with a radiative energy transfer mechanism, where the photons emitted during the UC process are absorbed by the RuNO complexes. Consequently, controlled NO photorelease upon excitation at 980 nm was achieved, in agreement with the direct photorelease observed for *cis*-[Ru­(dc-bpy)_2_(SO_3_)­(NO)]^+^ under blue-light irradiation. The amount of NO released progressively increased from 0.095 μmol L^–1^ after 30 min to 1.015 μmol L^–1^ after 180 min of irradiation, corresponding to ca. 5.8 and 62.1 nmol of NO per mg of UCNP-RuNO, respectively. Additional photochemical and thermal control experiments further supported the light-mediated process and showed an increased thermal stability for release of NO from the RuNO complex after incorporation into the nanoplatform. Aiming at biological applications, the UCNP-RuNO system presented in vitro cell viability up to approximately 12 μg mL^–1^. Overall, these results demonstrate a multifunctional core–shell platform capable of controlled NO release under 980 nm excitation within the NIR biological window, whose performance may be further optimized through variations in formulation, concentration, excitation conditions, and UCNP composition.

## Supplementary Material



## Notes

Experimental procedures for the synthesis of the ligand, precursor ruthenium complex, lanthanide chlorides, UCNPs, PVP-capped UCNPs, UCNP@ZIF-8, and UCNP@ZIF-8-NH_2_; additional procedure for nitric oxide photorelease using the ruthenium complex alone; supplementary FTIR, UV–vis, PXRD, photoluminescence, FESEM, TEM, and EDS figures and tables for the complex and the synthesized materials; computational results for the ruthenium complex, including molecular orbital analysis and visualization; nitric oxide photorelease experiments performed with the ruthenium complex together with the cPTIO calibration curve; and supplementary figures and tables related to the structural, spectroscopic, morphological, photophysical, and nitric oxide photorelease characterization of the nanoplatform, as well as control experiments performed under 980 nm irradiation, thermal conditions, and in the dark (PDF)
