# Supplementary material for: Core–Shell Upconversion Platform Based on an Amino-Functionalized ZIF‑8 with a Covalently Anchored Ruthenium Nitrosyl Complex for Controlled Release of Nitric Oxide in Biological Media
Source: ACS Omega. 2026 Oct 14;11(42):65521–35. doi: 10.1021/acsomega.6c08430 (PMC13717750; doi:10.1021/acsomega.6c08430)
Supplement: Supplementary file 1 [file ao6c08430_si_001.pdf]

Core-Shell Upconversion Platform Based on an Amino-Functionalized ZIF-8 with a Covalently Anchored Ruthenium Nitrosyl Complex for Controlled Release of Nitric Oxide in Biological Media

Victor B. de Sousa[a], Giovanna N. S. A. O. Rocha[a], Dayane K. D. Do N. Santos[a], Francisco V. S. Leite[b], Walysson G. Pereira[b], Luiz G. F. Lopes[b], Eduardo H. S. de Sousa[b], Ricardo L. Longo[a], Severino A. Júnior[a]**

[a]Department of Fundamental Chemistry, Federal University of Pernambuco, Recife, Pernambuco, 50740-560, Brazil.

[b]Department of Organic and Inorganic Chemistry, Federal University of Ceará, Fortaleza, Ceará, 60440-900, Brazil.

*Corresponding author: severino.alvesjr@ufpe.br; lopeslu@dqoi.ufc.br

Supporting Information

Table of Contents

A. Synthetic procedures S3
B. Procedure for NO release testing S5
C. Supplementary figures and tables S6

A. Synthetic procedures

Synthesis of 4,4'-dicarboxy-2,2'-bipyridine (dc-bpy)

The compound was synthesized according to the methodology of Sprintschnik *et al.* (1977) with modifications. In an Erlenmeyer flask, 6.5 mL of concentrated sulfuric acid (H_2SO_4) and 7 mmol of sodium dichromate ($Na_2Cr_7O_7$) were added under magnetic stirring for a period of 1h. Then, 2 mmol of 4,4'-dimethyl-2,2'-bipyridine ($C_{12}H_{12}N_2$) was added to the solution and stirred for 1.5 hours, followed by the addition of 100 mL of ice-cold water, and the precipitation of a solid was observed. The solid was recovered by centrifugation at 5100 rpm for 5 min and washed several times with ice-cold water. The solid obtained was solubilized in 250 mL of an aqueous 10% sodium hydroxide (NaOH) solution and left under magnetic stirring for 1 hour, followed by the addition of concentrated hydrochloric acid (HCl) until pH 2.5 (measured with a pH paper strip) was reached. At this pH, precipitation of a solid was noted, and then the solid was recovered by centrifugation at 5100 rpm for 5 min, washed several times with distilled water, and dried by freeze-drying.

Synthesis of *cis*-[Ru(dc-bpy)$_2$(Cl)$_2$]

The synthesis was carried out according to the procedure described by Durham *et al.* (1980) with some modifications. In a one-neck flask, 1 mmol of ruthenium chloride ($RuCl_3 \cdot xH_2O$) and 4 mmol of lithium chloride (LiCl) were solubilized in 10 mL of dimethylformamide (DMF). Then, 2 mmol of dicarboxypyridine (4,4'-dicarboxy-2,2'-bipyridine/dc-bpy) was added to the solution. The system remained under mechanical agitation and then heated under reflux at 125 ºC for 8 hours. The solution was cooled to room temperature and 100 mL of ice-cold acetone was added to precipitate the solid. The solid was recovered by centrifugation at 5100 rpm for 5 min and washed several times with DMF:acetone (1:5) solution and dried at room temperature.

Synthesis of yttrium, ytterbium, and thulium chloride

The rare earth salts were obtained following the procedure used by our research group. 1 mmol of yttrium oxide (Y_2O_3), 10 mL of concentrated hydrochloric acid, and 20 mL of water were added to a beaker, followed by magnetic stirring and heating (~120 ºC) until it reached almost the point of dryness. Then 5 mL of distilled water was added and the pH was measured using a pH paper strip. This process (water addition and heating until near dryness) continued until pH within 5-6 (measured with a pH paper strip) was reached. After the complete evaporation of the solvent, a solid was recovered. This same procedure was followed to obtain ytterbium and thulium chloride salts.

Synthesis of $NaYF_4$: Yb,Tm nanoparticles (UCNPs)

Upconversion nanoparticles (UCNPs) capped with oleic acid were obtained based on the methodology of Hao *et al*. (2019). In a two-neck flask, the following quantities of reagents were added: 0.78 mmol of yttrium chloride hexahydrate ($YCl_3 \cdot 6H_2O$), 0.20 mmol of ytterbium chloride pentahydrate ($YbCl_3 \cdot 5H_2O$), 0.02 mmol of thulium chloride heptahydrate ($TmCl_3 \cdot 7H_2O$), 6 mL of oleic acid (AO), and 15 mL of 1-octadecene (1-ODE). The solution was placed under magnetic stirring and heated to 160 °C to solubilize the rare earth salts. Then, the solution was cooled to room temperature, and a second solution of 10 mL of methanol containing 2.5 mmol of sodium hydroxide (NaOH), and 4 mmol of ammonium fluoride (NH_4F) was added, which was kept under stirring and heating (120 ºC) for 30 min to evaporate the methanol. Then, the solution was placed under a flow of argon, reflux, and heated to 300 ºC, and the reaction continued for 1.5 hours, after which the solution became cloudy. The solid was recovered by centrifugation at 6000 rpm for 10 min washed several times with hexane:ethanol (1:1) solution and dried at room temperature.

Preparation of $NaYF_4$:Yb,Tm-PVP capped nanoparticles

To cover the nanoparticle by ZIF-8, the surface of the particle was capped with PVP according to the methodology of Hao *et al*. (2019) with some alterations. In a screw-top vial, 30 mg of yttrium fluoride nanoparticles ($NaYF_4$:Yb,Tm) and 10 mL of chloroform were added and placed in an ultrasound bath for 10 minutes to disperse the nanoparticles. Then, the system was placed under magnetic stirring and a solution of 2 mg of PVP (Mw = 10,000) in chloroform was added. The vial was capped, and the reaction continued for 24 hours at room temperature (~25 ºC). After this time, the solid was recovered by centrifugation at 14.000 rpm for 5 minutes, washed several times with ethanol:water (1:1) solution, and dried in a vacuum oven for 24 hours at 40 ºC.

Synthesis of the $NaYF_4$:Yb,Tm@ZIF-8 system

The system was obtained based on the procedure of Silva *et al*. In an Erlenmeyer flask, 30 mg of yttrium fluoride nanoparticles capped with PVP, 2.469 mmol of zinc nitrate hexahydrate ($Zn(NO_3)_2 \cdot 6H_2O$), 50 mL of methanol were added and placed in an ultrasound bath to disperse the nanoparticles for 10 min, followed by 30 min of magnetic stirring. Then, 50 mL of methanolic solution of 9.974 mmol of 2-methylimidazole (2-MeIm) was added under magnetic stirring. Within 15 minutes, the solution became turbid. The stirring was turned off, and the solution was allowed to rest for 24 hours at room temperature (~25 ºC). The solid was recovered by centrifugation at 6000 rpm for 30 min and washed several times with methanol and water.

Functionalization of the $NaYF_4$:Yb,Tm@ZIF-8 system with amino groups

The functionalized platform to anchor the complex was obtained by the modification of ZIF-8 with amine groups using the solvothermal method was performed according to Hu *et al*. (2019). 0.10 g of previously synthesized $NaYF_4$:Yb,Tm@ZIF-8 system and 1.67 mL of ethylenediamine ($C_2H_8N_2$) were added to a reactor. Then, the reactor was sealed and placed in the oven preheated. The reaction was performed at 145 ºC for 1 h and then at 110 ºC for 6 h. The solid was recovered by centrifugation at 6000 rpm for 30 min, washed several times with methanol and dried at room temperature (~25 ºC).

B. Procedure for NO release testing

Tests for NO/HNO detection

NO photorelease was monitored using a selective scavenger. A solution was prepared in phosphate buffer (0.1 mol·L^{-1}, pH 7.4) with 0.20 mmol·L^{-1} of *cis*-$[Ru(dc\text{-}bpy)_2(SO3)(NO)]^+$ and 0.20 mmol·L^{-1} of cPTIO. Then, this solution was irradiated at 440 nm with 200 W lamp and was monitored by UV-Vis spectroscopy. The spectra were measured at 25 ºC at times of 0 min, 30 min, 1 h, and 3 h, using the changes in the absorbance at 560 nm.

C. Supplementary figures and tables

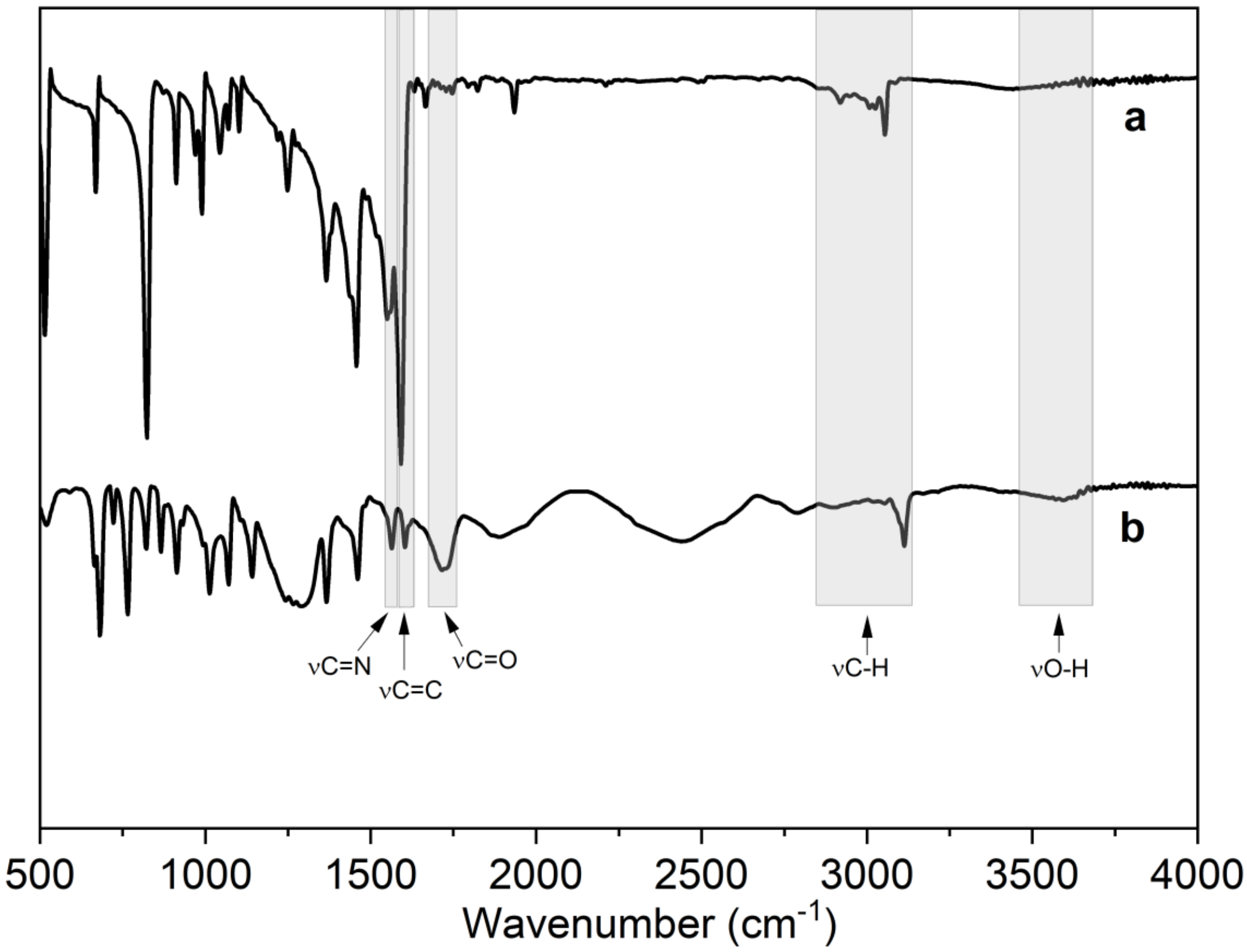

Figure S1. FTIR spectrum of 4',4-dimethyl-2',2-bipyridine (a) and 4',4-dicarboxy-2',2-bipyridine (b) in KBr pellet, showing the characteristic peaks of dicarboxy bipyridine (dc-bpy).

Table S1. Selected wavenumbers observed in the FTIR spectrum of 4',4-dimethyl-2',2-bipyridine and 4',4-dicarboxy-2',2-bipyridine.

Compound	Wavenumber (cm^{-1})	Assignment
**4',4-dimethyl-2',2-bipyridine**	3053, 2916	νC-H
	1591	νC=C
	1550	νC=N
**4',4-dicarboxy-2',2-bipyridine**	3589	νO-H
	3111, 2902	νC-H
	1720	νC=O
	1602	νC=C
	1562	νC=N

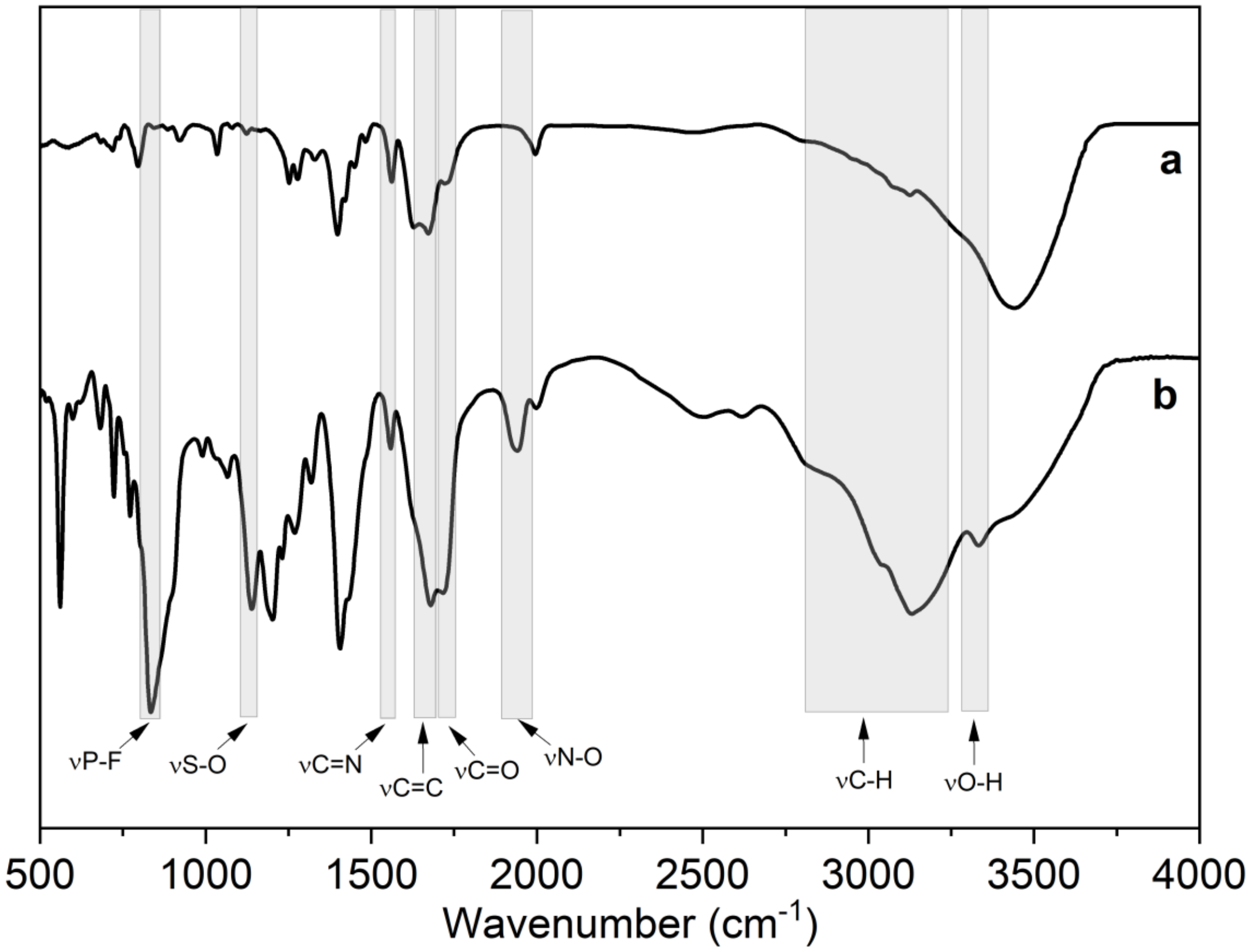

Figure S2. FTIR spectrum of *cis*-[Ru(dc-bpy)$_2$(Cl)$_2$] (a) and *cis*-[Ru(dc-bpy)$_2$(SO$_3$)(NO)]PF$_6$ (b) in KBr pellet, showing the characteristic peaks of *cis*-[Ru(dc-bpy)$_2$(SO$_3$)(NO)]PF$_6$.

Table S2. Selected wavenumbers observed in the FTIR spectrum of *cis*-[Ru(dc-bpy)$_2$(Cl)$_2$] and *cis*-[Ru(dc-bpy)$_2$(SO$_3$)(NO)]PF$_6$.

Compound	Wavenumber (cm−1)	Assignment
**cis-[Ru(dc-bpy)$_2$(Cl)$_2$]**	3437	νO-H
	3107, 2933	νC-H
	1720	νC=O
	1668	νC=C
	1558	νC=N
***cis*-[Ru(dc-bpy)$_2$(SO3)(NO)]PF$_6$**	3448	νO-H
	3116, 2914	νC-H
	1940	νN-O
	1718	νC=O
	1676	νC=C
	1556	νC=N
	1138	νS-O
	833	νP-F

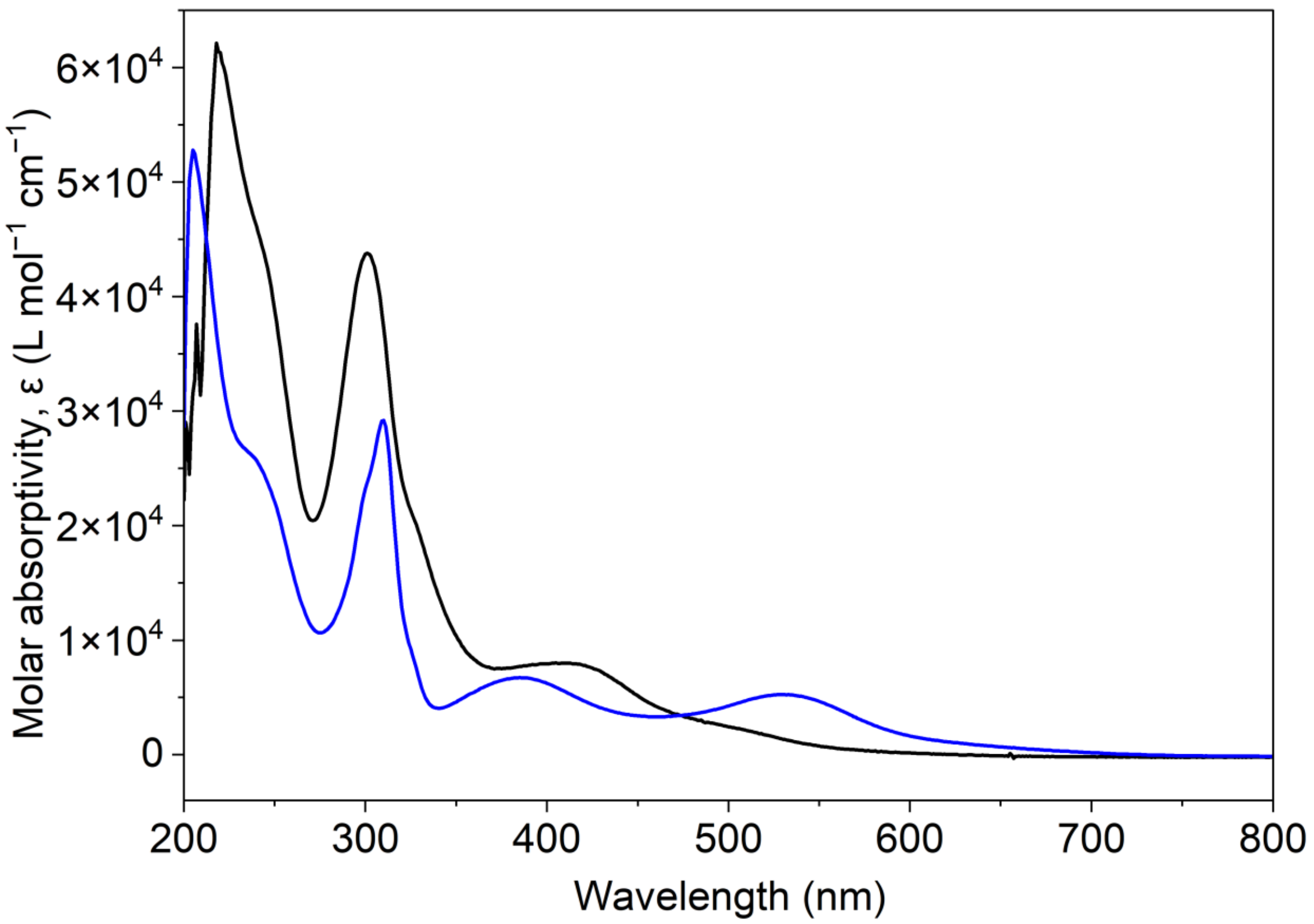

Figure S3. UV/Vis absorption spectrum of *cis*-[Ru(dc-bpy)$_2$(Cl)$_2$] in aqueous solution (2.2×10^{-5} mol·L^{-1}, black line) and of *cis*-[Ru(dc-bpy)$_2$(SO$_3$)(NO)]$^+$ in aqueous solution (1.1×10^{-5} mol·L^{-1}, blue line).

Table S3. Selected UV/Vis data of *cis*-[Ru(dc-bpy)$_2$(Cl)$_2$] in aqueous solution.

l$_{max}$ (nm)	Assignment
310	IL$\pi \rightarrow \pi^*$ (dc-bpy)
385	MLCT dπRu$^{II} \rightarrow \pi^*$ (dc-bpy)
531	MLCT dπRu$^{II} \rightarrow \pi^*$ (dc-bpy)

Table S4. Selected UV/Vis data of *cis*-[Ru(dc-bpy)$_2$(SO$_3$)(NO)]$^+$ in aqueous solution.

Molar absorptivity, ε (L mol^{-1} cm^{-1})	l$_{max}$ (nm)
1.72×10^4	301
6.00×10^3	340
2.87×10^3	429
1.09×10^3	512

Table S5. Assessment of functional and basis sets for reproducing the relevant vibration frequencies (in cm^{-1}) of the *cis*-$[Ru(dc\text{-}bpy)_2(SO_3)(NO)]^+$ complex.

Method	νO-H	νC-H	νN-O	νC=O	νC=C	νC=N	νSO$_3$
B3LYP/def2-SVP	3741	3230, 3204	1985	1863	1660	1621	1207
B3PW91/def2-SVP	3772	3239, 3199	2022	1881	1674	1637	993
BP86/def2-SVP	3616	3145, 3096	1907	1789	1617	1564	927
PBE0/def2-SVP	3801	3251, 3208	2055	1904	1704	1655	1010
M062X/def2-SVP	3810	3266, 3238	2092	1941	1722	1677	1037
WB97XD/def2-SVP	3832	3257, 3217	2048	1917	1715	1677	1027
B3LYP/def2-TZVP	3719	3220, 3198	1937	1814	1662	1602	1239
r2SCAN-3c	3724	3208, 3188	1933	1809	1653	1583	1238
B3LYP/LANL2DZ,6-31(G)d	3681	3257, 3246	1946	1846	1688	1617	969
B3PW91/LANL2DZ,6-31(G)d	3710	3255, 3242	1983	1864	1632	1623	990
BP86/LANL2DZ,6-31(G)d	3554	3161, 3134	1866	1773	1611	1559	924
PBE0/LANL2DZ,6-31(G)d	3734	3271, 3251	2017	1888	1698	1651	1007
M062X/LANL2DZ,6-31(G)d	3745	3265, 3255	2054	1920	1718	1672	1034
WB97XD/LANL2DZ,6-31(G)d	3760	3280, 3268	2024	1900	1711	1663	1022
**Experimental**	**3437**	**3132, 3034**	**1940**	**1720**	**1678**	**1558**	**1138**

Table S6. Assessment of functional and basis sets for reproducing the Ru-NO and N-O distances of the *cis*-$[Ru(dc\text{-}bpy)_2(SO_3)(NO)]^+$ complex and available crystallographic data.

Method	Ru−NO (Å)	N−O (Å)
B3LYP/def2-SVP	1.741	1.147
B3PW91/def2-SVP	1.732	1.143
BP86/def2-SVP	1.753	1.163
PBE0/def2-SVP	1.727	1.138
M062X/def2-SVP	1.708	1.131
WB97XD/def2-SVP	1.722	1.138
B3LYP/def2-TZVP	1.738	1.140
r2SCAN-3c	1.736	1.145
B3LYP/LANL2DZ,6-31G(d)	1.774	1.154
B3PW91/LANL2DZ,6-31G(d)	1.765	1.150
BP86/LANL2DZ,6-31G(d)	1.784	1.171
PBE0/LANL2DZ,6-31G(d)	1.761	1.145
M062X/LANL2DZ,6-31G(d)	1.752	1.138
WB97XD/LANL2DZ,6-31G(d)	1.756	1.145
B3LYP/LANL08,6-31G(d)	1.769	1.155
PBE0/LANL08,6-31G(d)	1.777	1.169
B3LYP/LANL08,6-311G(d,p)	1.772	1.143
PBE0/LANL08,6-311G(d,p)	1.779	1.158
**Experimental**	**1.737, 1.740, 1.745, 1.748, 1.751[a)]** **1.768[b)]**	**1.150, 1.135, 1.145, 1.133, 1.136[a)]** **1.136[b)]**

[a)] Wu *et al*. (2022). [b)] Codesido *et al*. (2014).

Table S7. Selected vibrational wavenumbers (cm^{-1}) for the *cis*-[Ru(dc-bpy)$_2$(SO_3)(NO)]$^+$ complex calculated at the B3LYP/def2-TZVP and r2SCAN-3c levels, experimental values in the infrared spectrum, and the proposed assignments.

B3LYP	r2SCAN-3c	Exp.	Assignment
3719	3724	3448	νO-H
3220-3198	3208-3188	3116-2914	νC-H
1937	1933	1940	νN-O
1814	1809	1718	νC=O
1662	1653	1676	νC=C
1602	1583	1556	νC=N
1239	1238	1138	νS-O

Table S8. Selected structural data for the *cis*-[Ru(dc-bpy)$_2$(SO_3)(NO)]$^+$ complex calculated at the B3LYP/def2-TZVP (G09) and r2SCAN-3c (Orca 5.1) levels, and available experimental values.

Atoms	B3LYP	r2SCAN-3c	Exp.
Ru19 - N40 (NO)	1.738	1.736	1.736 – 1.751
N40 - O41 (NO)	1.140	1.145	1.133 – 1.149
Ru19 - S42 (SO_3)	2.522	2.503	2.376
Ru19 - N15 (dc-bpy)	2.269	2.213	2.140
Ru19 - N16 (dc-bpy)	2.135	2.100	2.091
Ru19 - N28 (dc-bpy)	2.091	2.059	2.068
Ru19 - N37 (dc-bpy)	2.147	2.121	2.091
Ru19 - N40 - O41 (NO)	176.5	175.5	173.7
(NO) N40 - Ru19 - S42 (SO_3)	86.4	86.9	86.2
Ru19 - S42 - O43	103.8	104.7	106.6
Ru19 - N15 - C3 (dc-bpy)	126.3	125.9	125.4
(NO) N40 - Ru19 - N37 (dc-bpy)	164.4	163.3	171.0
(NO) N40 - Ru19 - 28N (dc-bpy)	95.2	95.1	96.9

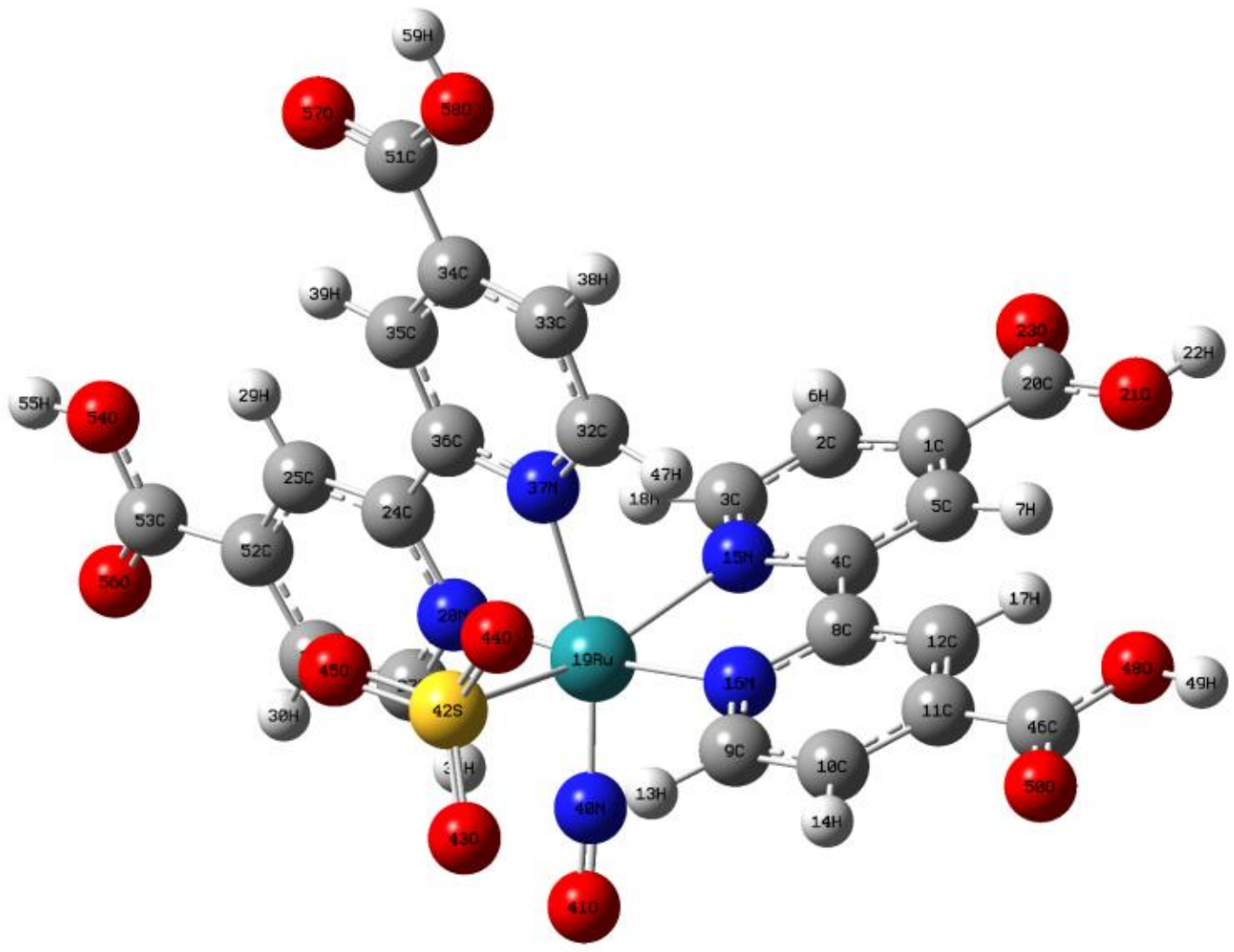

Figure S4. Optimized structure of the ion *cis*-[Ru(dc-bpy)$_2$(SO$_3$)(NO)]$^+$ complex at the B3LYP/def2-TZVP level with the atom identity and numbering. The cartesian coordinates are available upon request to the authors.

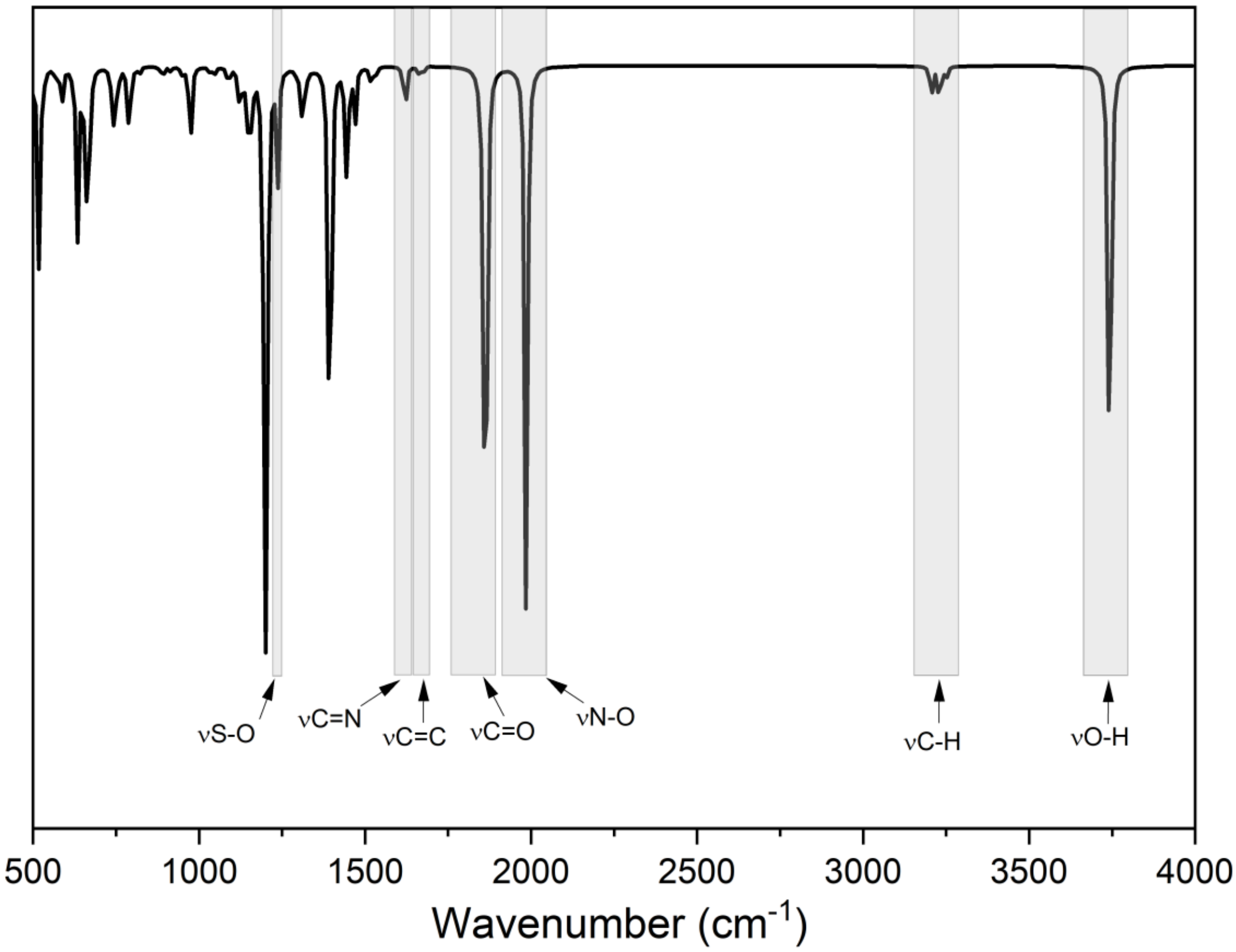

Figure S5. Calculated FTIR spectrum of the *cis*-$[Ru(dc\text{-}bpy)_2(SO_3)(NO)]^+$ complex at the B3LYP/def2-TZVP level and the assignments of the main peaks. The simulated spectrum was obtained using a gaussian lineshape with width at half-height of 5 cm^{-1}.

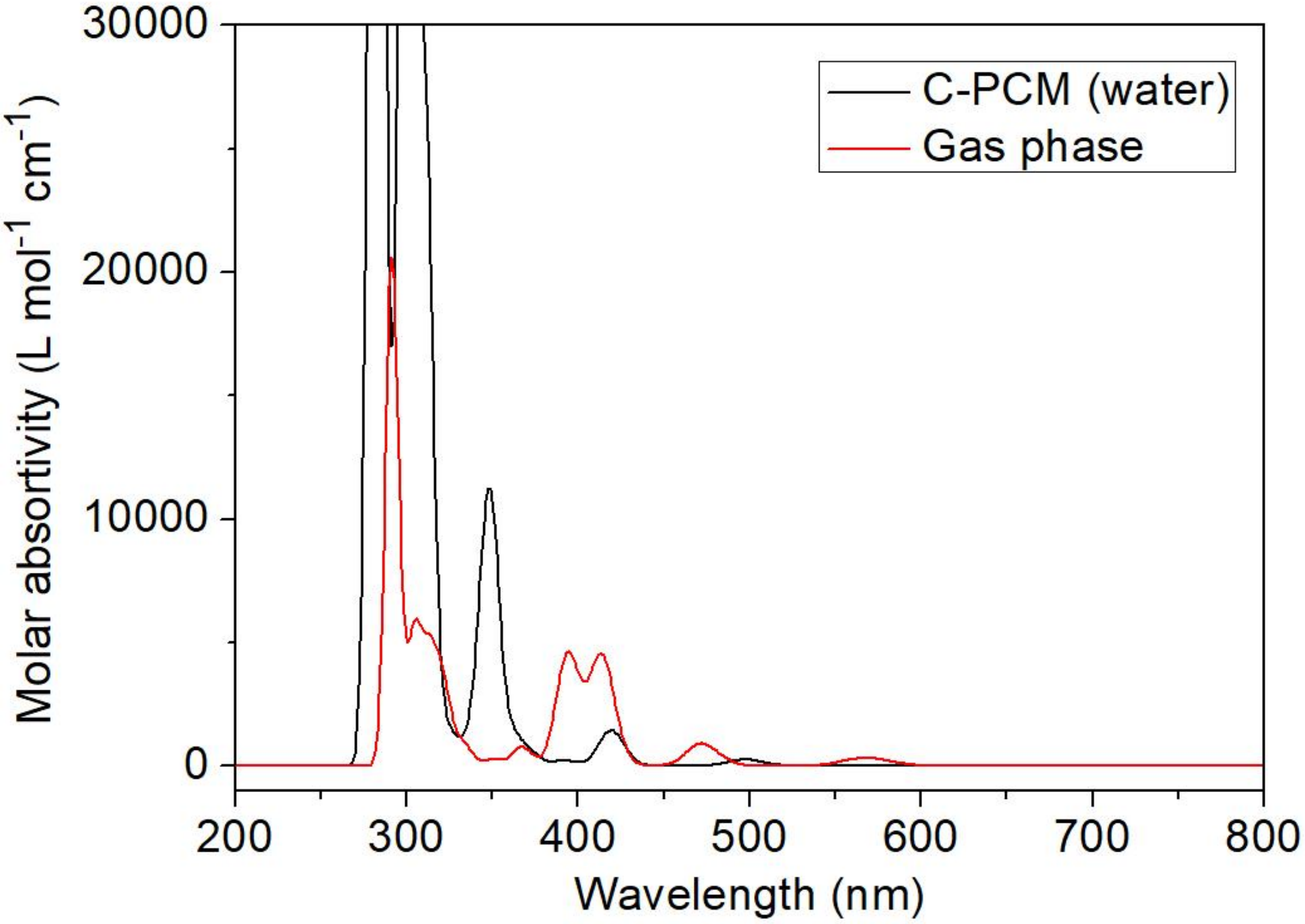

Figure S6. Calculated UV/Vis spectrum of *cis*-[Ru(dc-bpy)$_2$(SO$_3$)(NO)]$^+$ complex in water (C-PCM solvation model) and at the TD-DFT/CAM-B3LYP/def2-SVP level. The simulated spectrum was obtained using a Gaussian lineshape with width at half-height of 500 cm^{-1}.

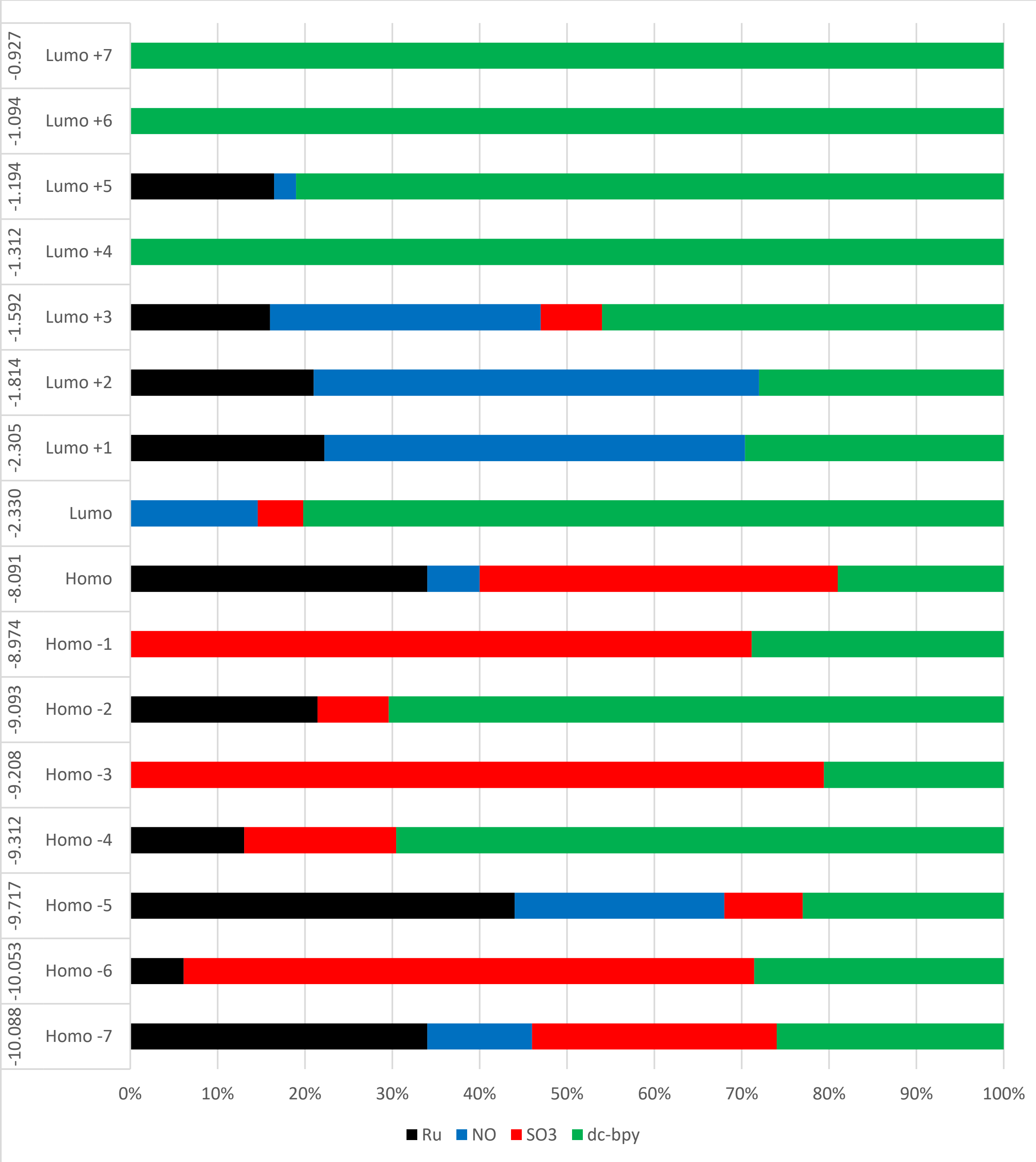

Figure S7. Molecular orbital energy (in eV) and composition of fragment orbitals in the *cis*-[Ru(dc-bpy)$_2$(SO$_3$)(NO)]$^+$ complex. Fragment notation: Ru (black), NO$^+$ (blue), SO$_3$ (red) and dc-bpy (green).

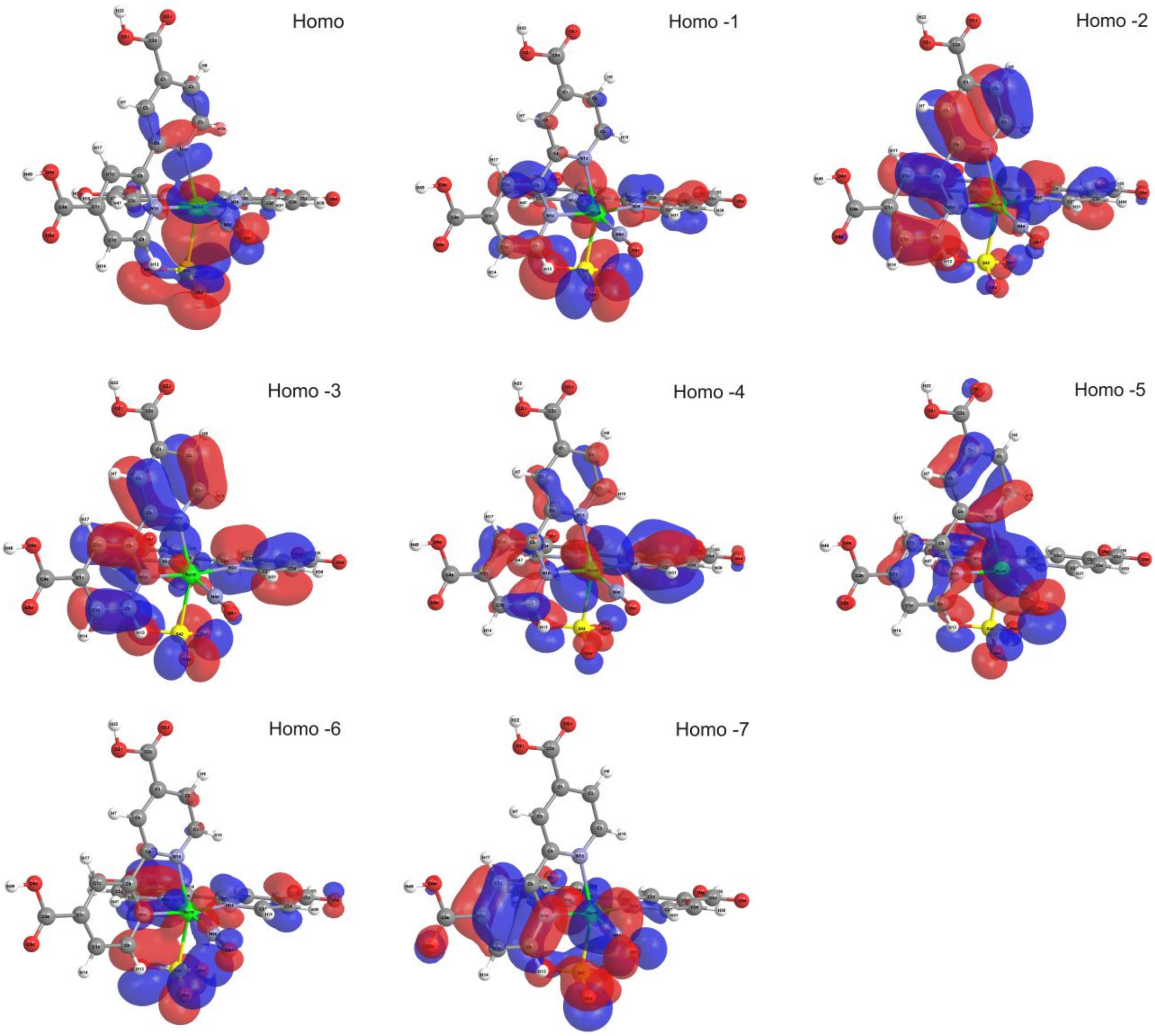

Figure S8. Illustrations of highest occupied molecular orbitals (HOMOs) of *cis*-[Ru(dc-bpy)$_2$(SO$_3$)(NO)]$^+$ at the B3LYP/def2-TZVP level. The surfaces correspond to an isovalue of 0.1.

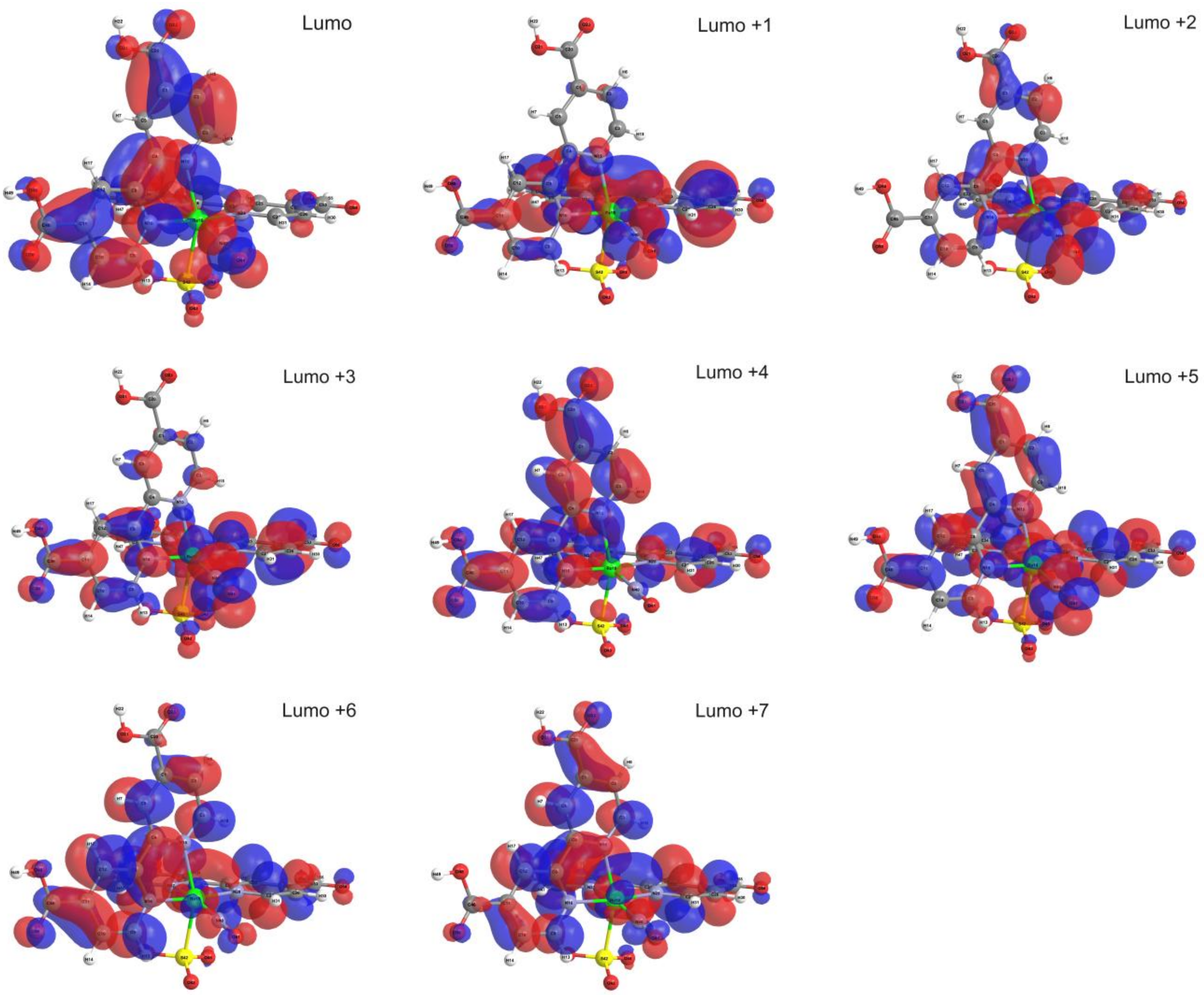

Figure S9. Illustrations of lowest unoccupied molecular orbitals (LUMOs) of *cis*-[Ru(dc-bpy)$_2$(SO$_3$)(NO)]$^+$ at the B3LYP/def2-TZVP level. The surfaces correspond to an isovalue of 0.1.

Table S9. Calculated absorption wavelength (λ in nm) and oscillator strength (f) with three functionals and def2-TZVP basis set at the r2SCAN-3c optimized geometry for the isolated (gas) *cis*-[Ru(dc-bpy)$_2$(SO_3)(NO)]$^+$ complex and in water (modeled with C-PCM).

CAM-B3LYP				LC-PBE				wB97X-D3			
Gas		C-PCM (water)		Gas		C-PCM (water)		Gas		C-PCM (water)	
λ	f	λ	f	λ	f	λ	f	λ	f	λ	f
520	0.0022	442	0.0016	473	0.0006	420	0.0002	502	0.0017	430	0.0012
444	0.0080	381	0.0131	389	0.0044	347	0.0063	423	0.0066	367	0.0111
406	0.0217	372	0.0004	354	0.0013	334	0.0012	374	0.0013	362	0.0004
384	0.0021	355	0.0022	337	0.0065	332	0.0021	371	0.0255	347	0.0022
379	0.0191	344	0.0048	336	0.0004	318	0.0053	359	0.0208	336	0.0038
363	0.0025	328	0.0595	317	0.0193	312	0.0029	354	0.0042	312	0.0459
351	0.0043	321	0.0046	314	0.0166	308	0.0095	344	0.0032	306	0.0283
341	0.0031	315	0.0055	310	0.0130	298	0.0064	314	0.0059	300	0.0092
327	0.0038	307	0.0104	306	0.0175	274	0.2677	310	0.0003	298	0.0116
317	0.0014	301	0.0082	288	0.0004	273	0.4375	302	0.0104	293	0.0043
314	0.0344	295	0.2920	279	0.0111	268	0.0962	299	0.0105	286	0.3255
309	0.0078	293	0.2737	270	0.1377	265	0.0761	299	0.0100	284	0.3150
305	0.0089	279	0.0256	264	0.1279	256	0.0358	297	0.0220	268	0.0867
301	0.0050	277	0.1302	264	0.0936	246	0.0546	287	0.0223	268	0.0670
298	0.0020	274	0.0884	259	0.1401	237	0.0532	279	0.0481	261	0.0938
295	0.0041	268	0.0274	247	0.1213	236	0.0120	276	0.0645	259	0.0052
293	0.0047	263	0.0041	246	0.0246	235	0.0002	273	0.0466	249	0.0125
291	0.0089	258	0.0395	245	0.0013	235	0.0015	271	0.1049	248	0.0106
285	0.0553	258	0.0077	245	0.0062	234	0.0008	270	0.2234	248	0.0033
283	0.0598	255	0.0255	244	0.0013	234	0.0014	268	0.0080	247	0.0018
281	0.0650	253	0.0254	241	0.0283	230	0.2022	267	0.0260	247	0.0003
278	0.1937	252	0.0026	239	0.0141	227	0.0607	262	0.0001	246	0.0199
276	0.1187	252	0.0026	237	0.0238	225	0.0721	261	0.0004	245	0.0168
268	0.0005	251	0.0152	234	0.0896	223	0.1477	260	0.0038	242	0.0386
267	0.0957	251	0.0013	232	0.0751	223	0.0717	259	0.0047	240	0.0800
267	0.0049	249	0.0103	230	0.0490	221	0.3554	259	0.0072	235	0.1119
265	0.0030	249	0.0943	227	0.0684	215	0.0774	256	0.0636	233	0.3797
265	0.0039	248	0.0288	227	0.0098	214	0.1324	254	0.0862	231	0.1358
257	0.0102	245	0.0111	225	0.0507	212	0.4596	249	0.0033	230	0.0401
255	0.0123	243	0.0108	223	0.0159	209	0.0820	244	0.0341	228	0.0566
255	0.0142	241	0.1541	221	0.2860	207	0.1230	241	0.0350	227	0.0075
253	0.0177	239	0.0452	218	0.0045	206	0.2451	239	0.0328	227	0.2387
252	0.0572	238	0.2219	217	0.2057	203	0.0624	238	0.0789	225	0.0800
252	0.0071	236	0.0091	216	0.1484	202	0.0282	238	0.0147	223	0.0506
250	0.0199	235	0.0709	213	0.0052	200	0.1716	237	0.0133	221	0.2011
249	0.0149	234	0.0189	213	0.0249	198	0.2283	235	0.0953	221	0.0691

Table S10. Calculated electronic transitions of *cis*-[Ru(dc-bpy)$_2$(SO$_3$)(NO)]$^+$ complex in water (C-PCM solvation model) at the TD-DFT/CAM-B3LYP/def2-SVP level. Wavelength λ (in nm), oscillator strength f, dominant molecular orbitals (coefficients larger than 0.1) in the transition, and assignment. Values in parenthesis refer to the isolated (gas phase) complex. Notation: H = HOMO, L = LUMO, MLCT = metal-to-ligand charge transfer, IL = intra-ligands, and ItL = inter-ligands.

λ (nm)	f	MOs	Coefficient	Assignment
497.3 (567.4)	0.0012 (0.0015)	H−5 → L+2	0.18404	MLCT π∗(NO) ← dπRu^{II}
		H → L	−0.16894	
		H → L+1	0.33281	
		H → L+2	0.52413	
419.2 (472.1)	0.0067 (0.0042)	H → L	0.37735	MLCT π∗(dc-bpy) ← dπRu^{II}
		H → L+1	0.27394	
		H → L+3	0.37972	
		H → L+5	0.26045	
		H → L+7	0.10089	
		H → L+8	−0.16390	
348.9 (413.9)	0.0470 (0.0203)	H → L+1	0.49862	MLCT π∗(NO) ← dπRu^{II}
		H → L+2	−0.30209	
		H → L+3	−0.29265	
		H → L+5	−0.17787	
302.5 (313.1)	0.1721 (0.0180)	H−4 → L	−0.13681	IL π∗ ← π(dc-bpy)
		H−3 → L	−0.28754	
		H−3 → L+1	0.15977	
		H−2 → L	0.44719	
		H−1 → L+1	−0.23360	
		H → L+9	0.12239	
299.0 (305.3)	0.1396 (0.0202)	H−4 → L+1	−0.12418	IL π∗ ← π(dc-bpy)
		H−3 → L	0.29253	
		H−3 → L+1	0.21117	
		H−2 → L+1	0.28898	
		H−1 → L	0.11217	
		H−1 → L+1	−0.15930	
		H−1 → L+3	0.17596	
		H−1 → L+5	0.10768	
		H−1 → L+8	0.12650	
		H → L+9	0.26806	

Table S10 (continue).

λ (nm)	f	MOs	Coefficient	Assignment
284.8 (293.4)	0.2144 (0.0144)	H−4 → L+1	0.47906	ItL π∗(NO) ← π(dc-bpy)
		H−4 → L+2	−0.19515	
		H−4 → L+3	−0.14205	
		H−4 → L+9	−0.12294	
		H−2 → L+1	0.10014	
		H−2 → L+2	−0.10332	
		H → L+4	0.16284	
		H → L+5	0.16355	
278.6 (290.4)	0.1105 (0.0599)	H−4 → L	0.12856	ItL π∗(NO) ← π(dc-bpy)
		H−4 → L+3	−0.10048	
		H−3 → L+2	−0.11817	
		H−3 → L+3	0.10647	
		H−1 → L	−0.16987	
		H−1 → L+2	0.19098	
		H−1 → L+5	0.13336	
		H → L+3	−0.10468	
		H → L+4	−0.39735	
		H → L+6	−0.11868	
		H → L+8	−0.19396	

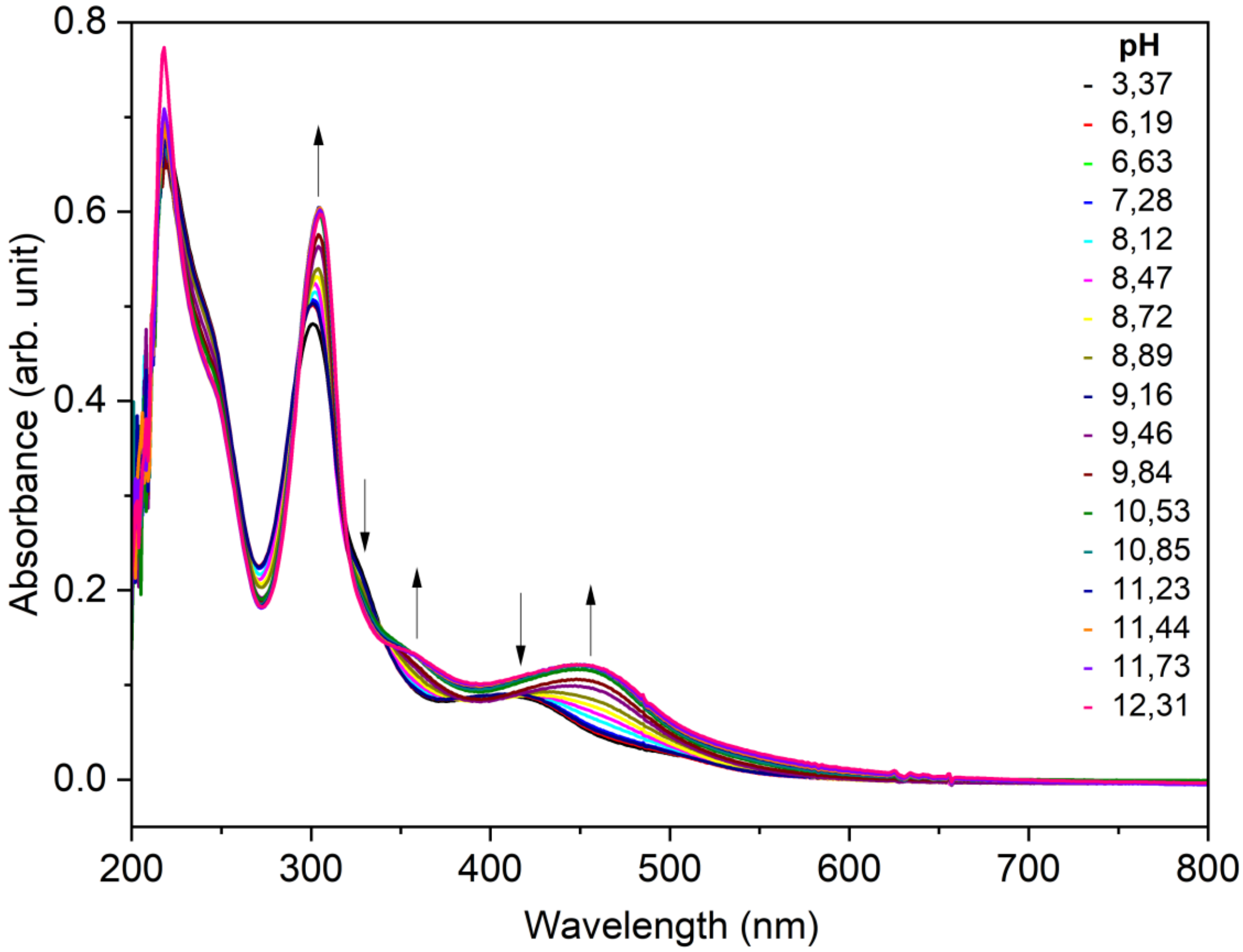

Figure S10. Absorption spectra of *cis*-[Ru(dc-bpy)$_2$(SO$_3$)(NO)]$^+$ complex in 1.1×10^{-5} mol·L^{-1} aqueous HTFA solution (0.1 mol·L^{-1}) at pH ranging from 3.37 to 12.31 (measured with a pH-meter).

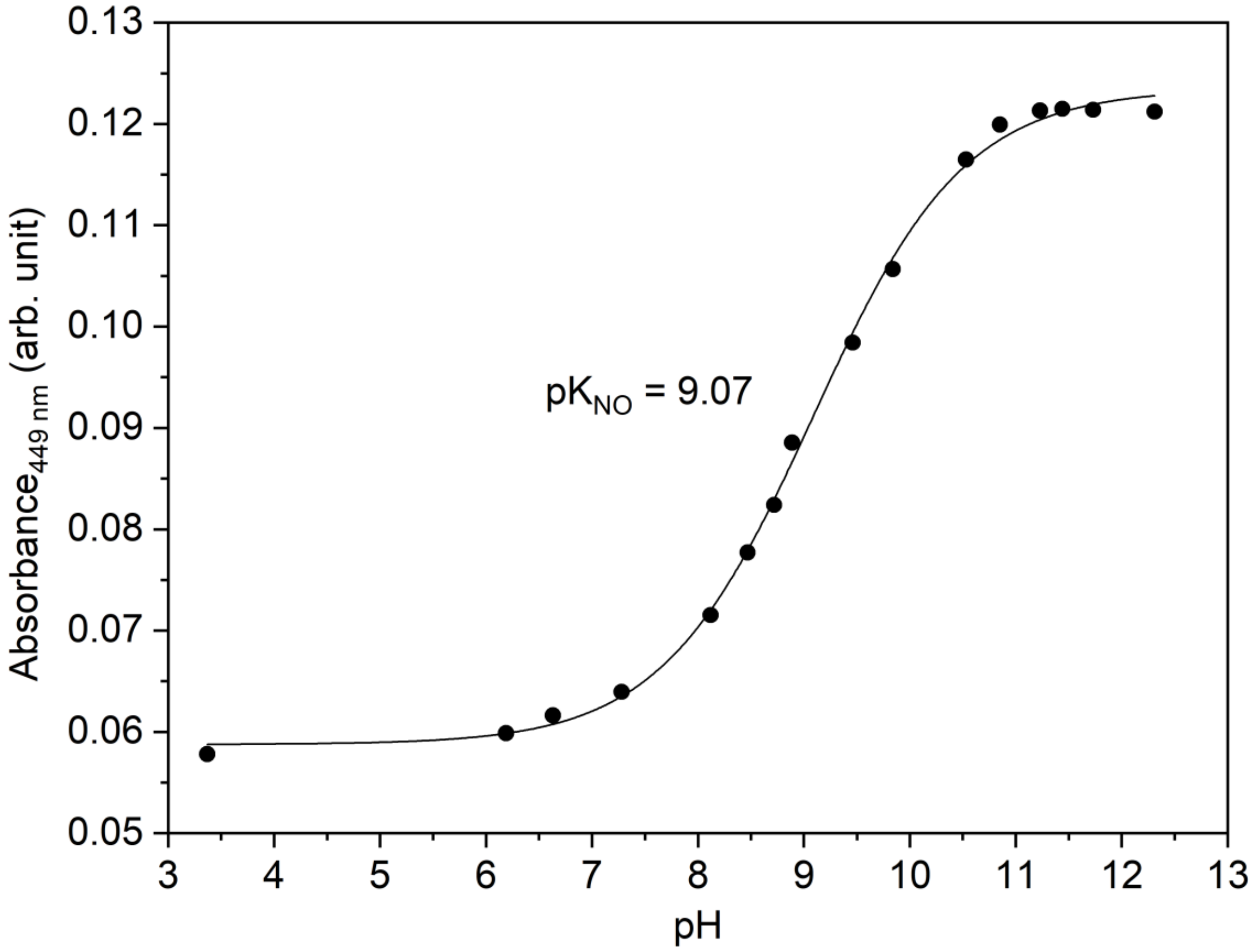

Figure S11. Titration curve of *cis*-$[Ru(dc\text{-}bpy)_2(SO_3)(NO)]^+$ complex in 1.1×10^{-5} mol·L^{-1} aqueous HTFA solution (0.1 mol·L^{-1}) monitoring the absorbance at 449 nm.

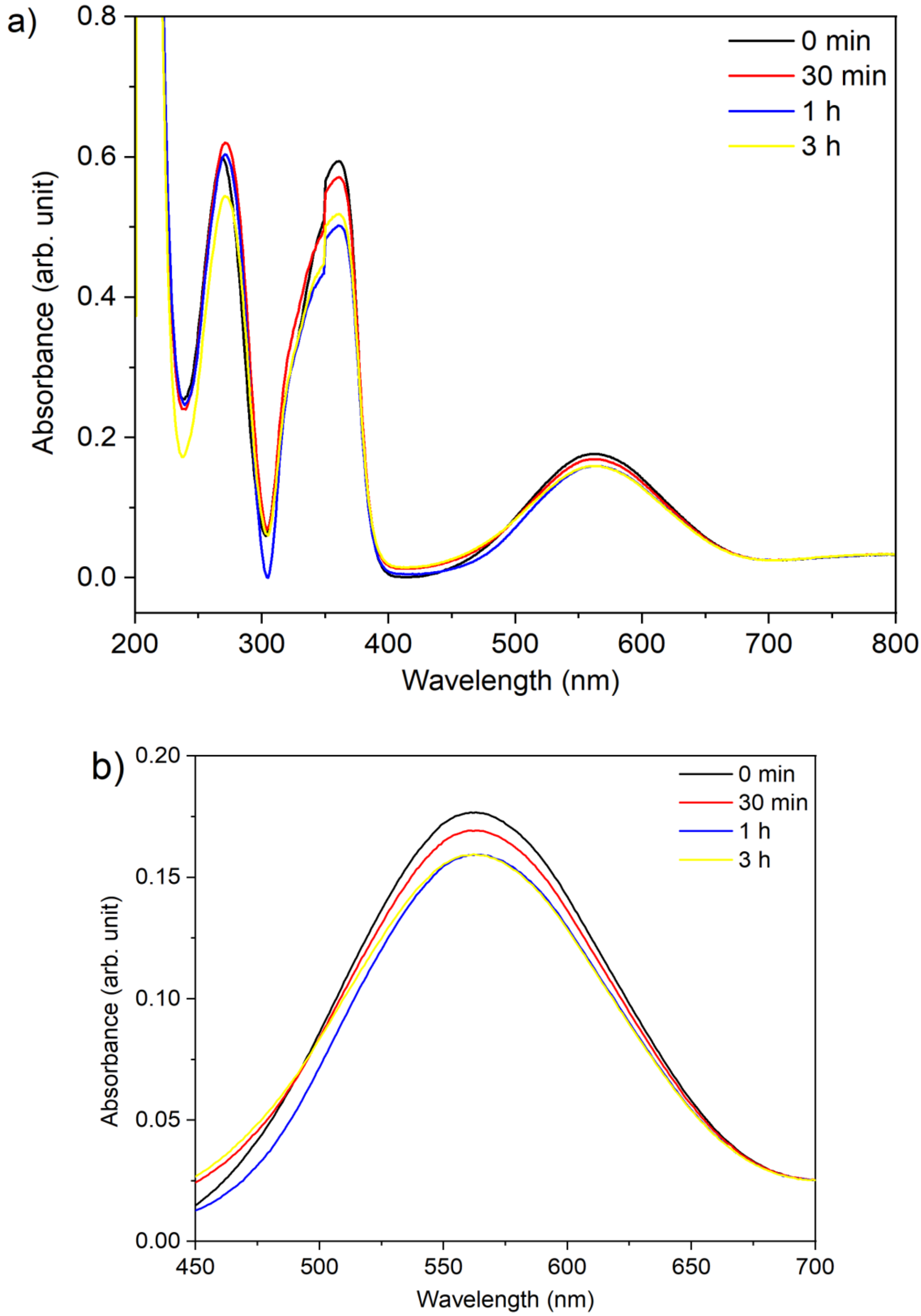

Figure S12. Absorption spectra of cPTIO during the photolysis of *cis*-$[Ru(dc\text{-}bpy)_2(SO_3)(NO)]^+$ complex in phosphate buffer solution 0.1 mol·L^{-1}, pH 7.4 at 25 ºC) irradiated during 0 to 3 hours (a), and the band at 560 nm (b).

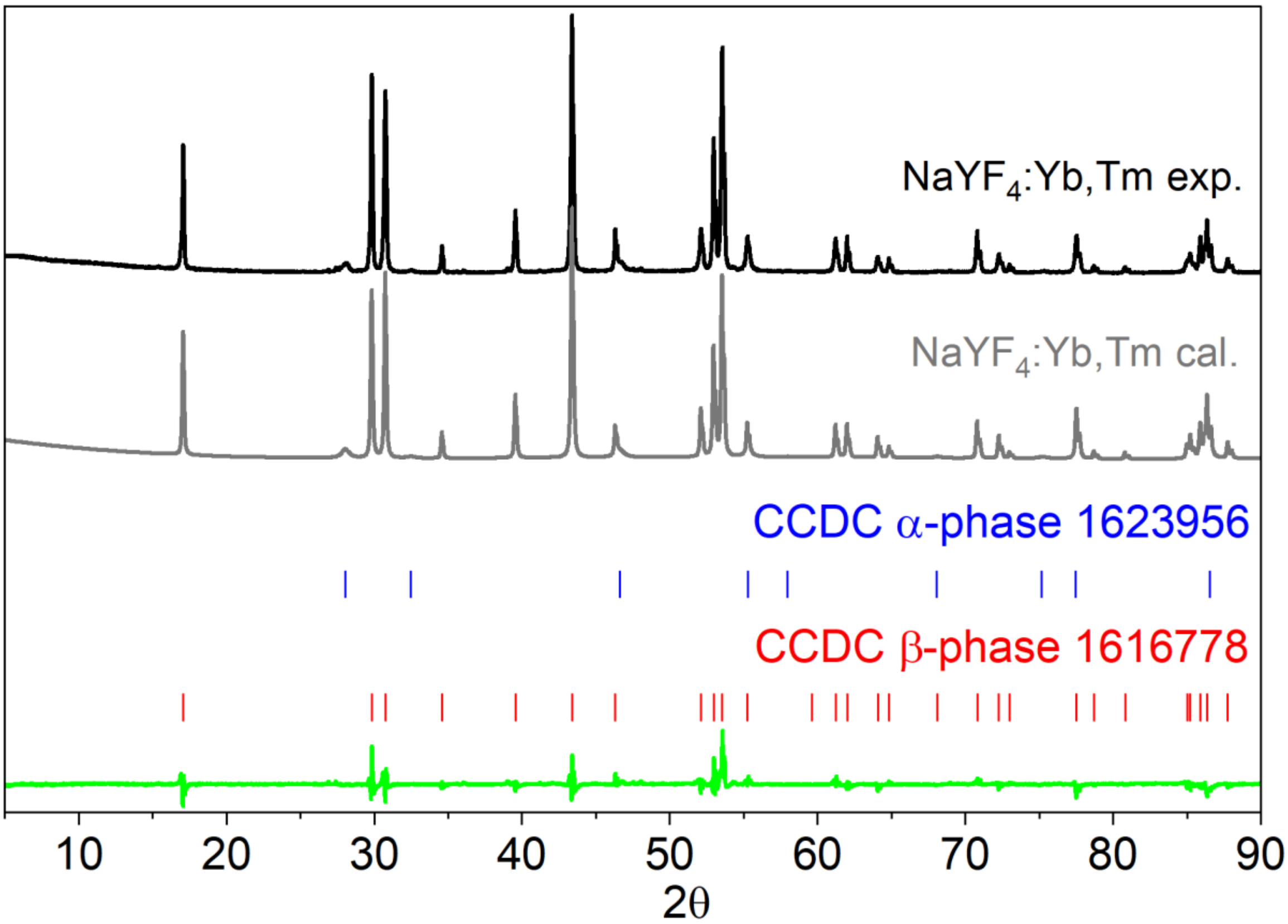

Figure S13. The PXRD pattern of as-synthesized $NaYF_4$:Yb,Tm (black) compared to the PXRD pattern calculated for the reference phase (gray), with the reflection positions (a-phase, blue and b-phase, red), and the difference plot (green). R_w = 8.68%.

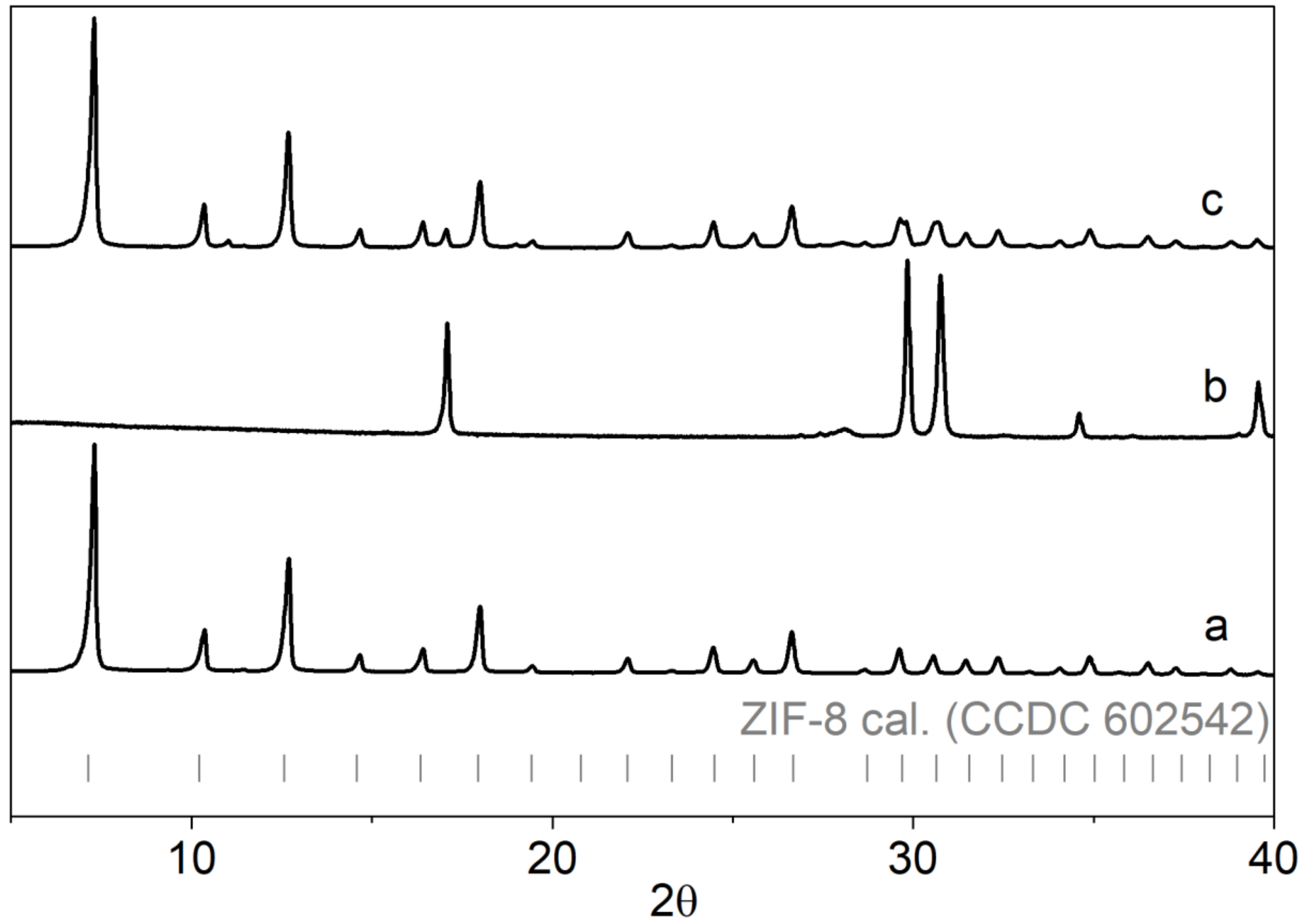

Figure S14. The PXRD pattern of as-synthesized ZIF-8 (a) compared to the reflection positions for the reference phase (VELVOY, gray), and with the PXRD pattern of as-synthesized $NaYF_4$:Yb,Tm (b), and $NaYF_4$:Yb,Tm@ZIF-8-NH_2 (c).

Table S11. Crystallographic data of ZIF-8 used in the simulated XRD diffractogram. Reference CCDC number 602542.

**Empirical formula**	$C_{24}H_{30}N_{12}O_{10}Zn_3$
**Crystal system**	Cubic
**Space group**	I - 43*m*
**Z**	4
**Unit cell dimensions**	a = b = c = 16.991 Å (a = b = γ = 90°)
**Unit cell volume**	40905.2 Å^3
**Surface area**	1947 m^2 g^{-1}
**Pore diameter**	11.6 Å
**Pore volume**	0.663 cm^3 g^{-1}
**Density (calculated)**	1141 kg m^3
**Crystal size**	0.16 × 0.10 × 0.10 mm^3

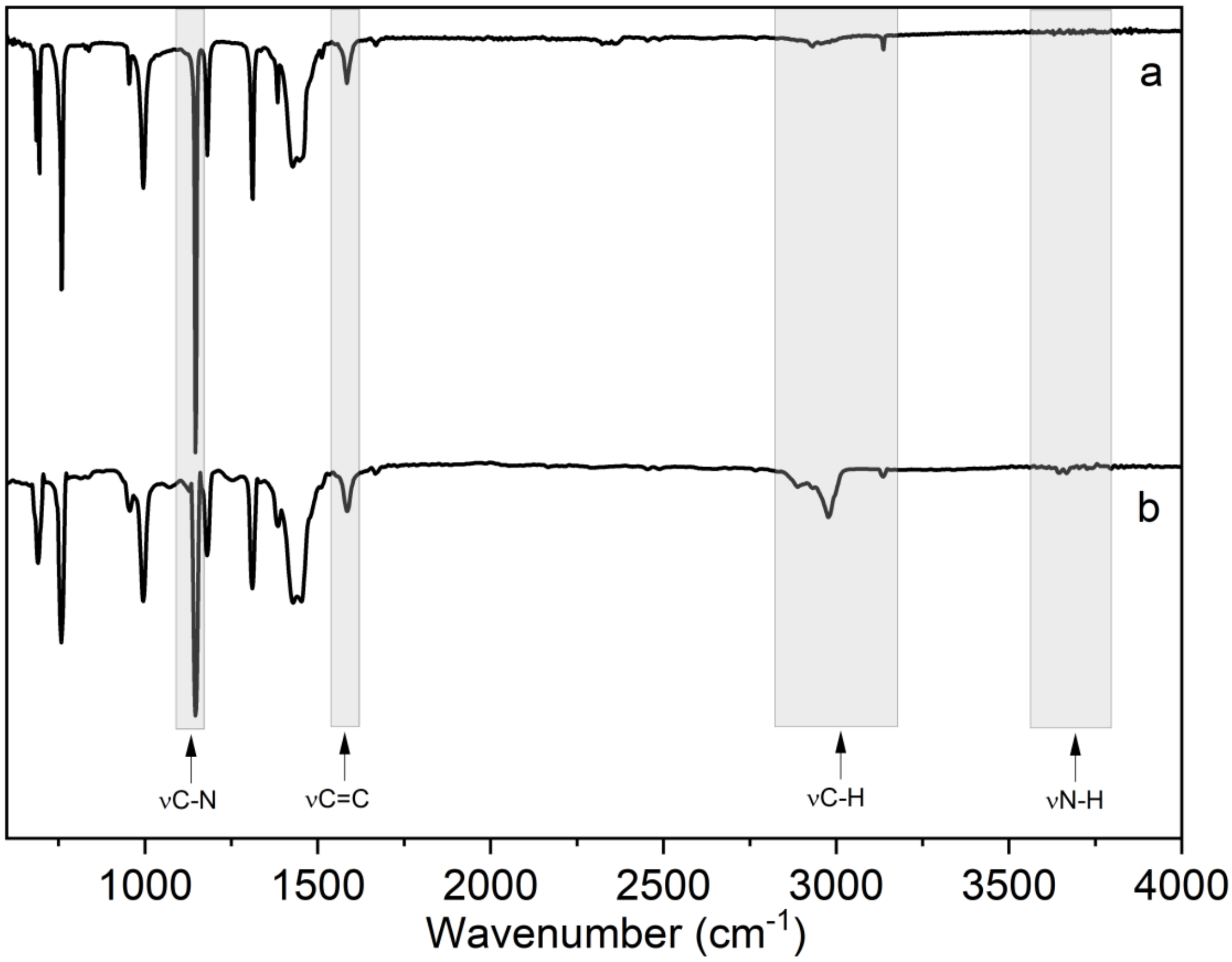

Figure S15. FTIR spectra of ZIF-8 (a) and $NaYF_4$:Yb,Tm@ZIF-8-NH_2 (b) in KBr pellet, showing the main peaks of the functional groups.

Table S12. Main frequencies observed in the FTIR spectra of ZIF-8 and $NaYF_4$:Yb,Tm@ZIF-8-NH_2.

Compound	Wavenumber (cm^{-1})	Assignment
**ZIF-8**	3136, 2920	νC-H
	1583	νC=C
	1145	νC-N
**$NaYF_4$:Yb,Tm@ZIF-8-NH_2**	3750, 3600	νN-H
	3134, 2931	νC-H (Im)[a]
	2975	νC-H (EDA)[b]
	1583	νC=C
	1145	νC-N

[a] Im = Imidazole. [b] EDA = Ethylenediamine.

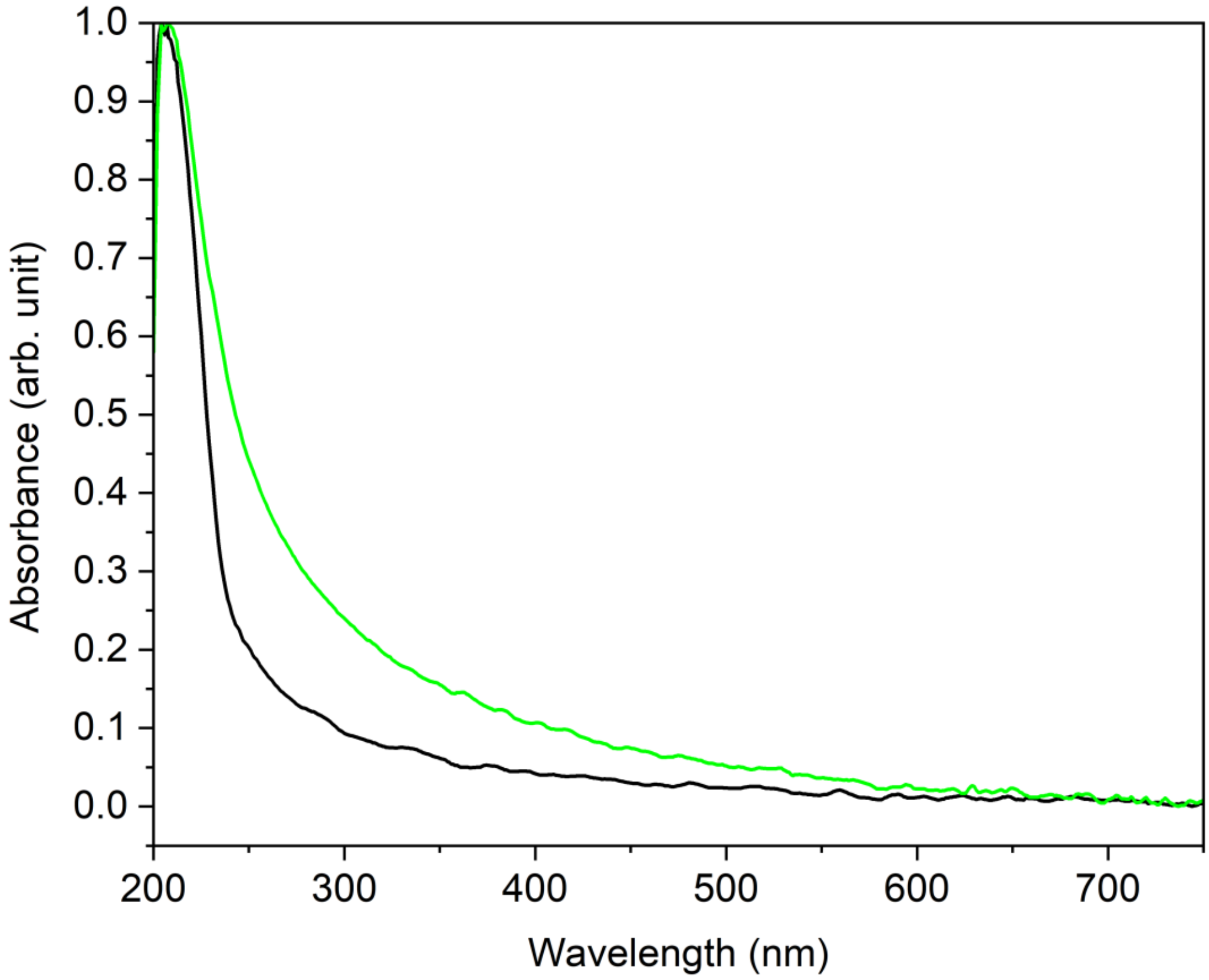

Figure S16. Absorption spectra of $NaYF_4$:Yb,Tm@ZIF-8 (black line) and $NaYF_4$:Yb,Tm@ZIF-8-NH_2 (green line) in methanol suspension (50 ppm).

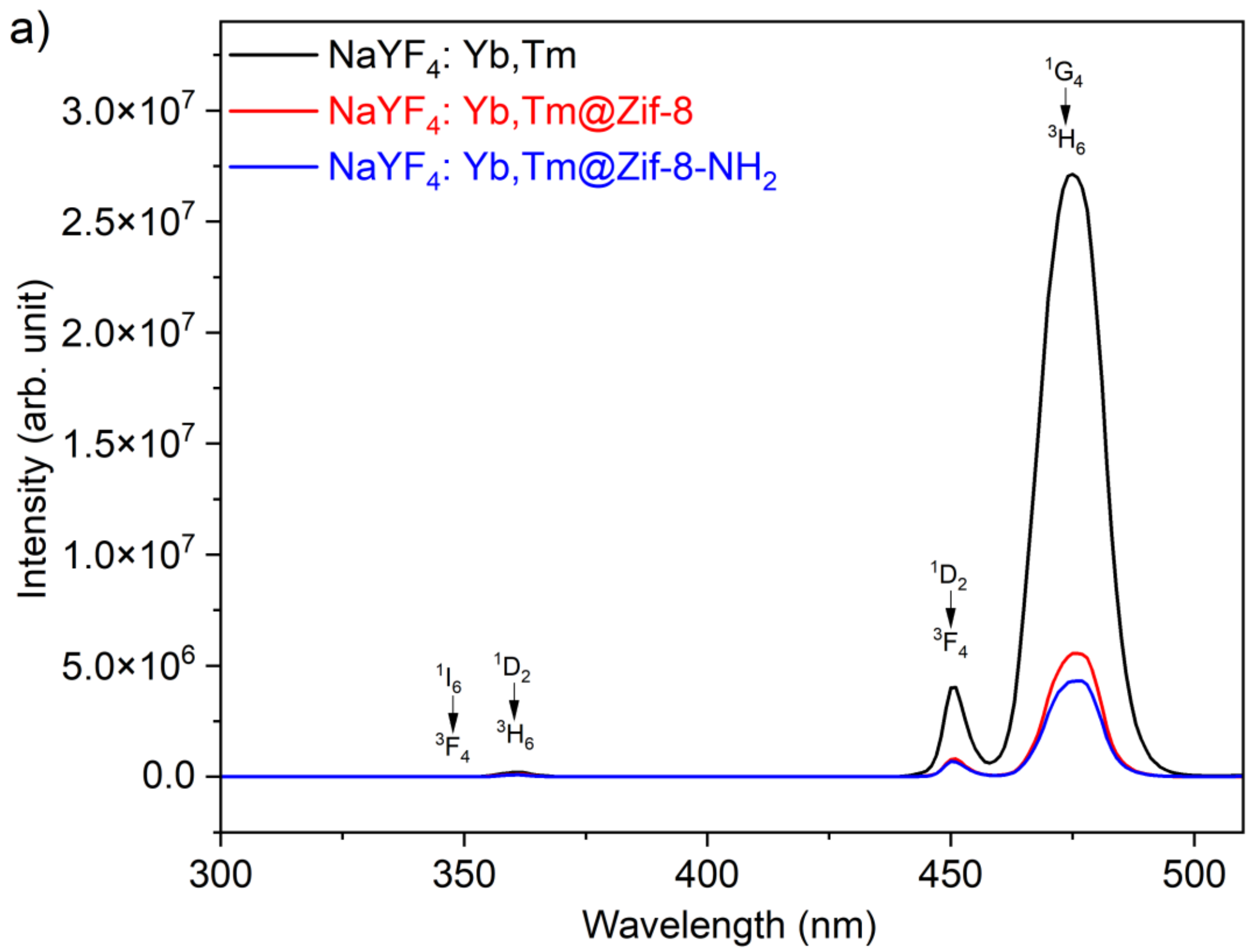
a)
NaYF4: Yb,Tm
NaYF4: Yb,Tm@Zif-8
NaYF4: Yb,Tm@Zif-8-NH2
Intensity (arb. unit)
Wavelength (nm)
1G4
3H6
1D2
3F4
1I6
3F4
1D2
3H6
3.0×10^7
2.5×10^7
2.0×10^7
1.5×10^7
1.0×10^7
5.0×10^6
0.0
300
350
400
450
500

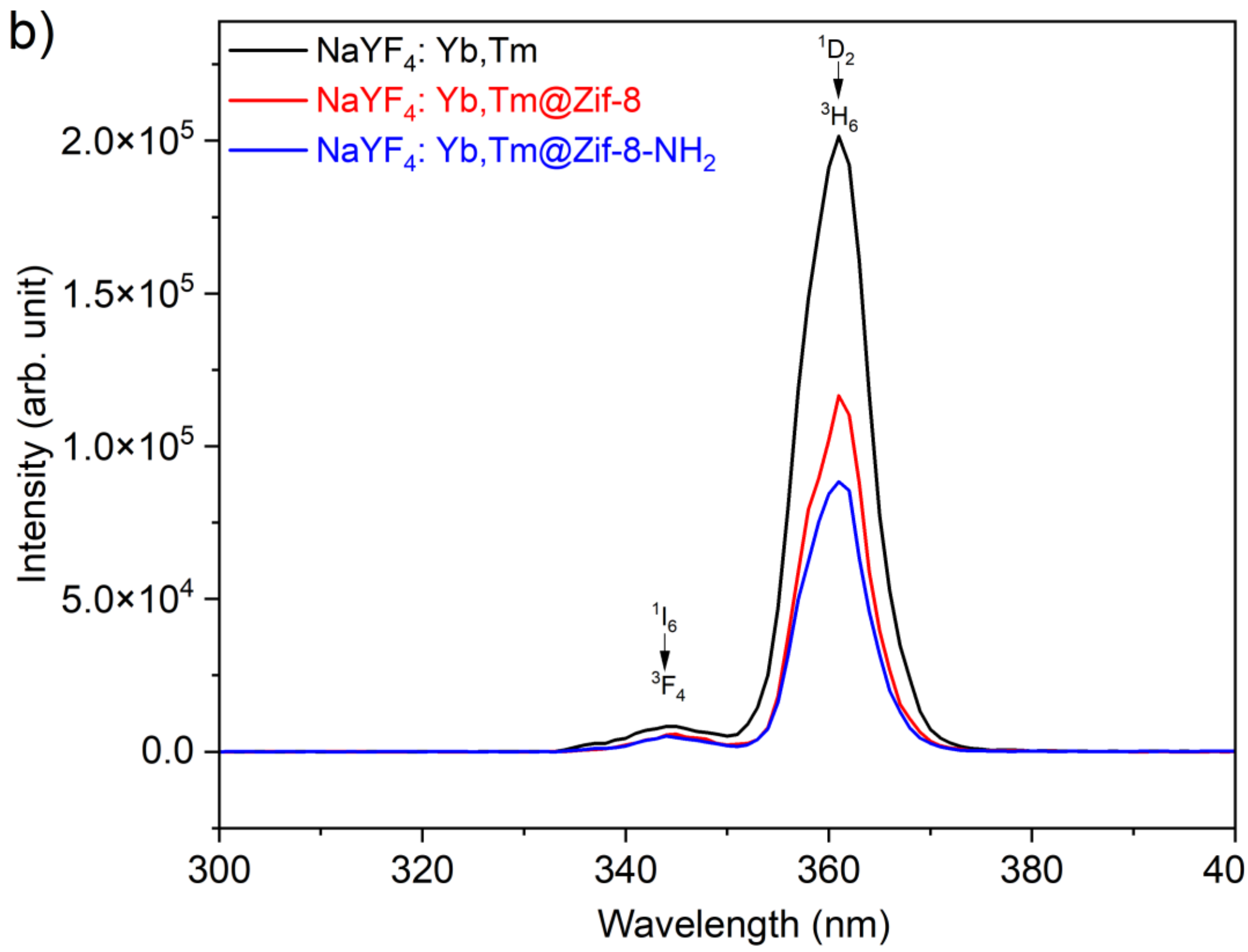
b)
NaYF4: Yb,Tm
NaYF4: Yb,Tm@Zif-8
NaYF4: Yb,Tm@Zif-8-NH2
Intensity (arb. unit)
Wavelength (nm)
1D2
3H6
1I6
3F4
2.0×10^5
1.5×10^5
1.0×10^5
5.0×10^4
0.0
300
320
340
360
380
40

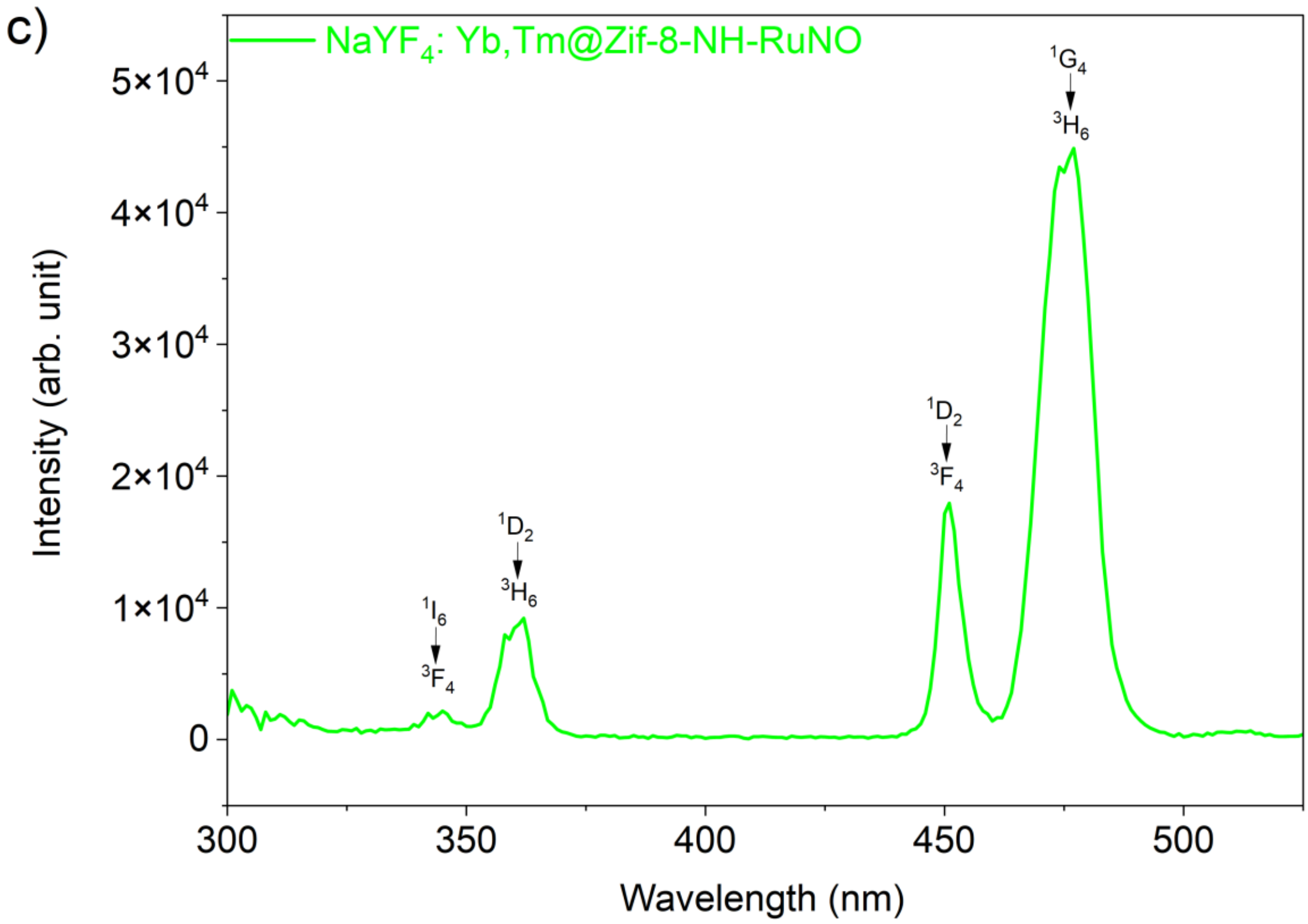

Figure S17. Upconversion emission spectra of $NaYF_4$:Yb,Tm, $NaYF_4$:Yb,Tm@ZIF-8, $NaYF_4$:Yb,Tm@ZIF-8-NH_2 nanoparticles upon excitation at 980 nm with a 0.5 W diode laser (a), expanded view of 300 – 400 nm region (b) and $NaYF_4$:Yb,Tm@ZIF-8-NH-RuNO (c).

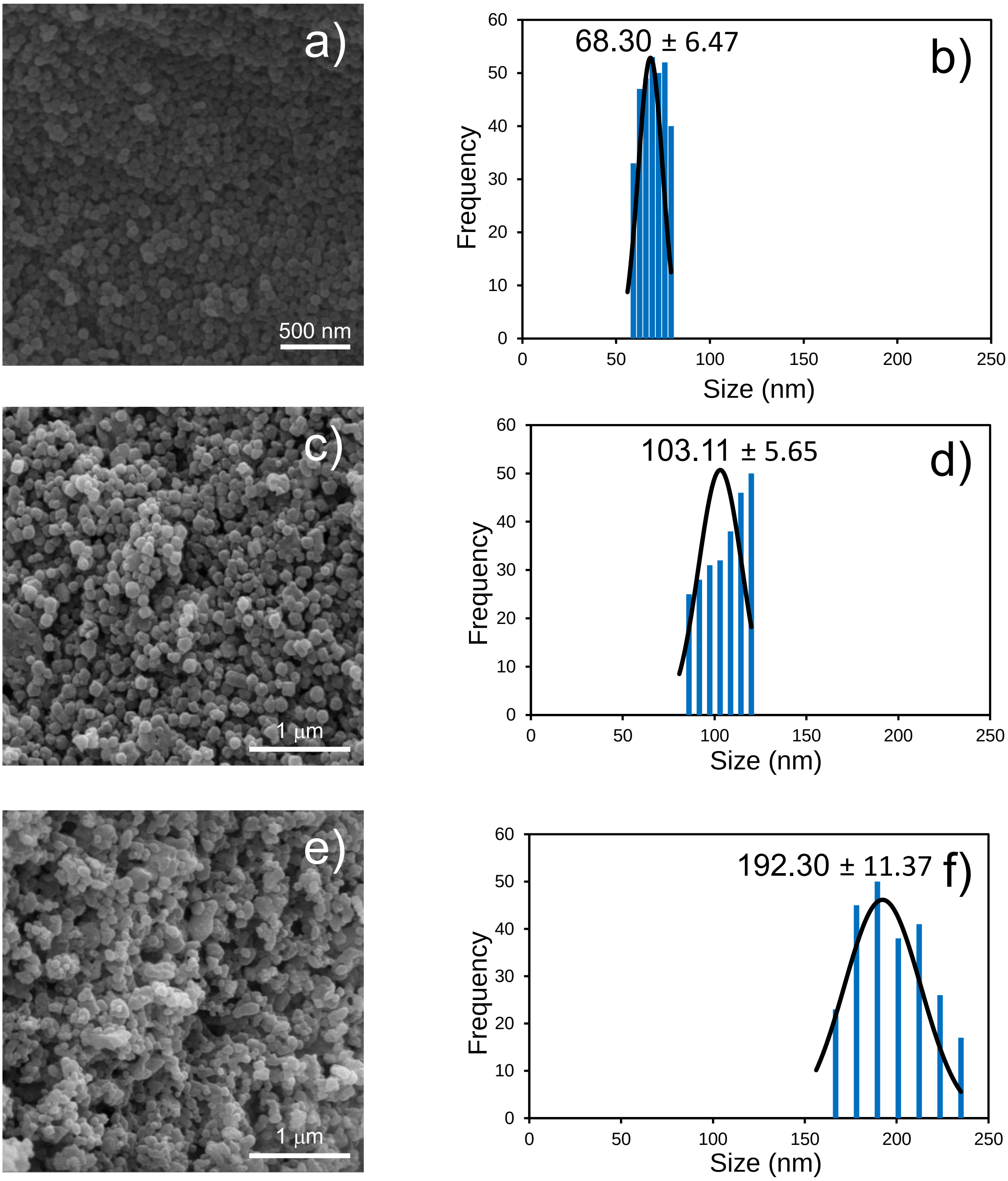

Figure S18. Scanning electron microscope (SEM) images of ZIF-8 (a), $NaYF_4$:Yb,Tm@ZIF-8 (c), and $NaYF_4$:Yb,Tm@ZIF-8-NH_2 (e), and their respective mean size distributions (b), (d) and (f).

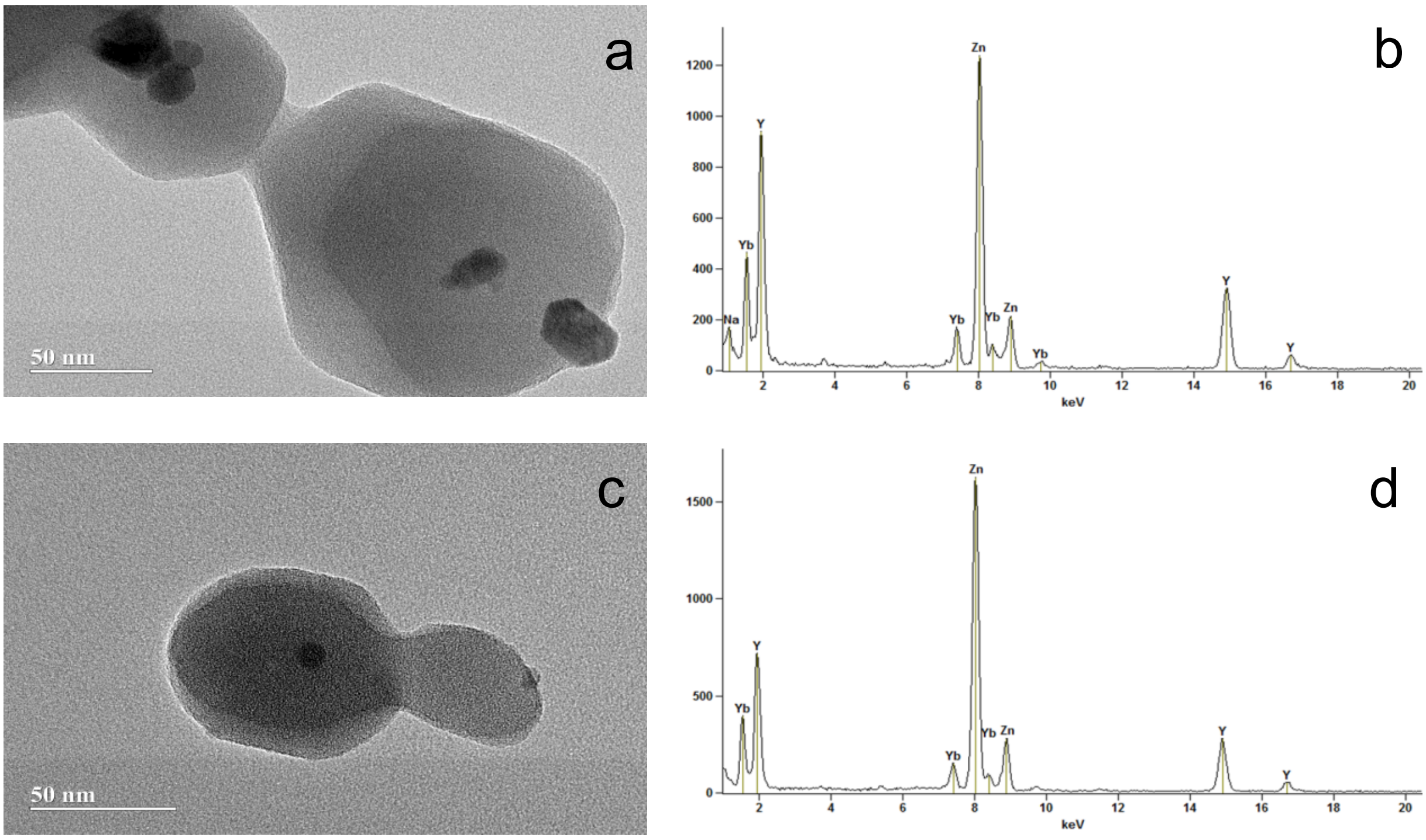

Figure S19. Transmission electron microscope (TEM) image of $NaYF_4$:Yb,Tm@ZIF-8-NH_2 (a and c) and energy dispersive X-ray spectroscopy (EDX) of image a (b) and c (d). The white circles denote the region where the EDX analysis was performed.

Wavenumber (cm^{-1})	Assignment
3408	νO-H (water of hydration)
3124, 2920	νC-H (bipyridine)
2966	νC-H (ethylenediamine)
1909	νN-O
1618	νC=O (amide I, overlapped with aromatic ring vibrations of dc-bpy/framework)
1546	δ(N–H) + ν(C–N) (amide II, overlapped with pyridyl/imidazolate ring vibrations)
1153	νC-N (imidazole)
1136	νS-O
862	νP-F

Table S13. Main frequencies observed in the FTIR spectrum of $NaYF_4$:Yb,Tm@ZIF-8-NH-[Ru(dc-bpy)$_2$(SO_3)(NO)] in KBr pellet.

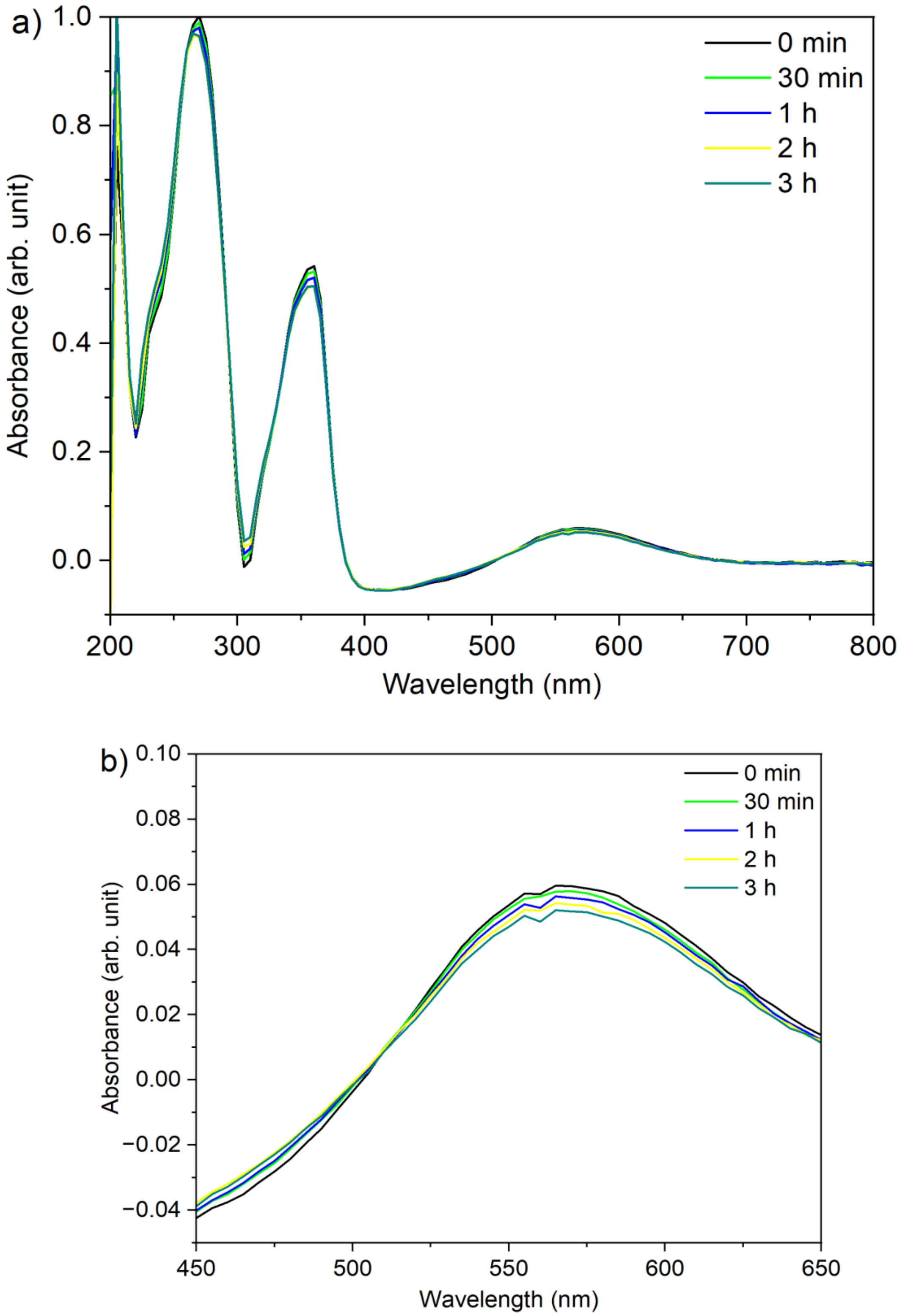

Figure S20. UV-Vis absorption spectrum of phosphate buffer solution (0.1 mol·L^{-1}, pH 7.4, 25 °C) with cPTIO (2-(4-carboxyphenyl)-4,4,5,5-tetramethylimidazoline-1-oxyl-3-oxide potassium salt) during irradiation with a diode laser at 980 nm (a), and expanded view of 450 – 650 nm region (b).

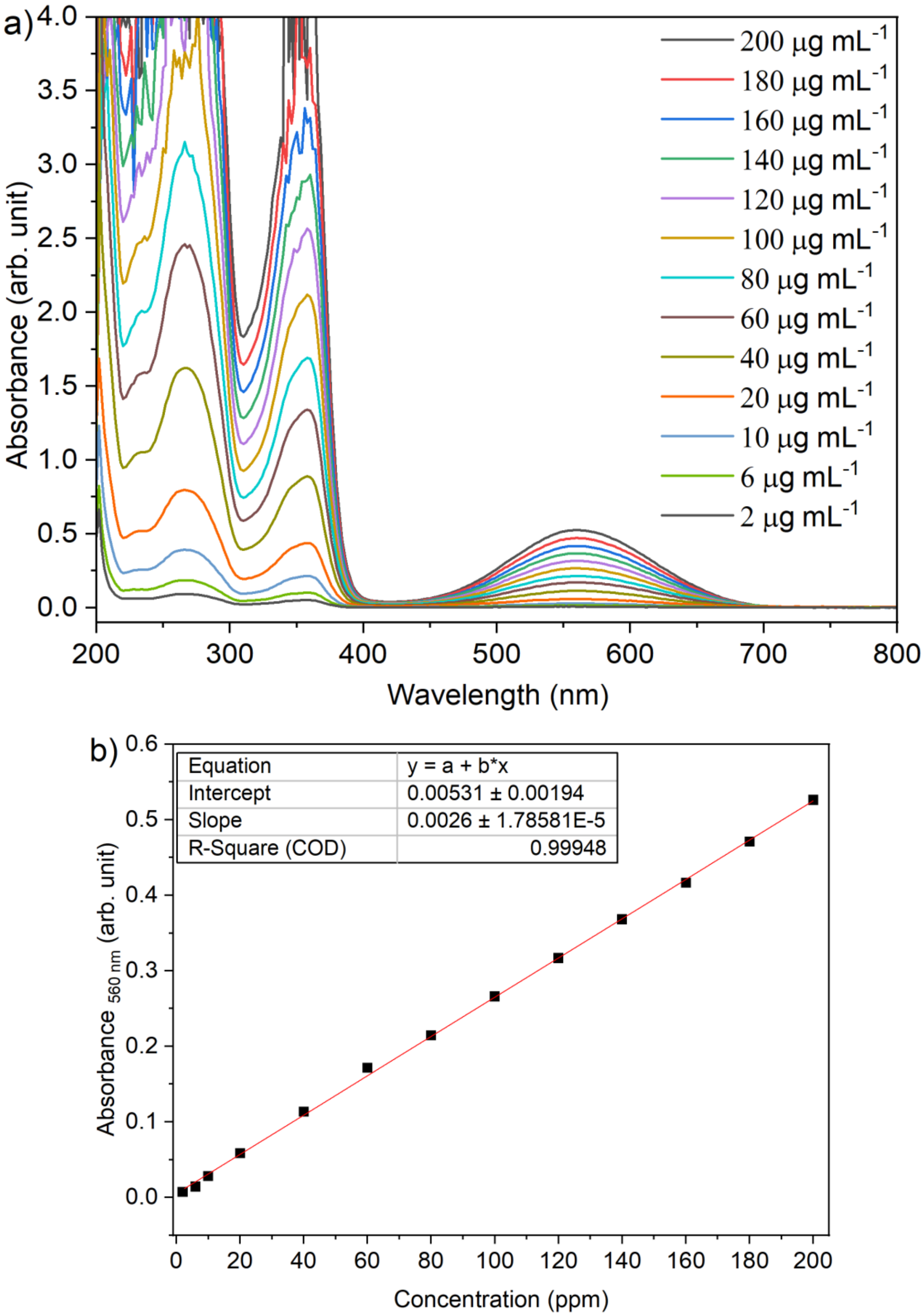

Figure S21. UV/Vis spectrum of phosphate buffer solution (0.1 M, pH 7.4 at 25 ºC) with cPTIO at concentrations ranging from 2 to 200 μg mL^{-1} (a), and calibration curve with a linear regression of absorbance at 560 nm and concentration (ppm), with adjusted-R^2 of 0.9995, intercept of 0.0053±0.002, and slop of 0.0026±0.00002 (b).

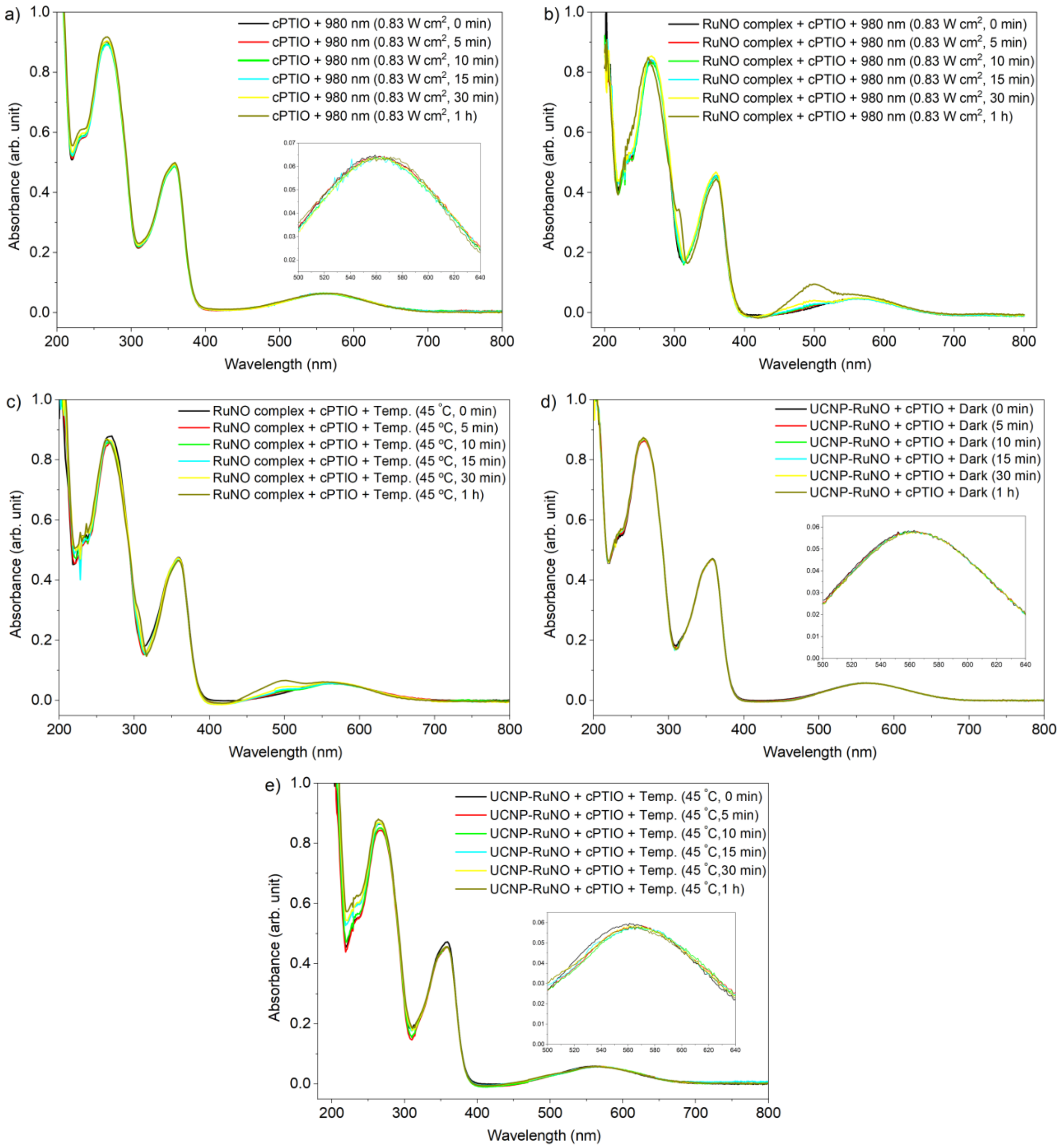

Figure S22. UV–Vis absorption spectra obtained in phosphate buffer solution (0.1 M, pH 7.4 at 25 °C) under different control conditions used to evaluate NO release and possible thermal or photochemical effects: (a) cPTIO under 980 nm irradiation (0. 83 W cm^{-2}); (b) RuNO complex in the presence of cPTIO under 980 nm irradiation (0. 83 W cm^{-2}); (c) RuNO complex in the presence of cPTIO at 45 °C; (d) UCNP–RuNO in the presence of cPTIO under dark conditions; and (e) UCNP–RuNO in the presence of cPTIO at 45 °C. Spectra were recorded at 0, 5, 10, 15, 30, and 60 min. Insets in panels (a), (d), and (e) highlight the 500–640 nm spectral region.

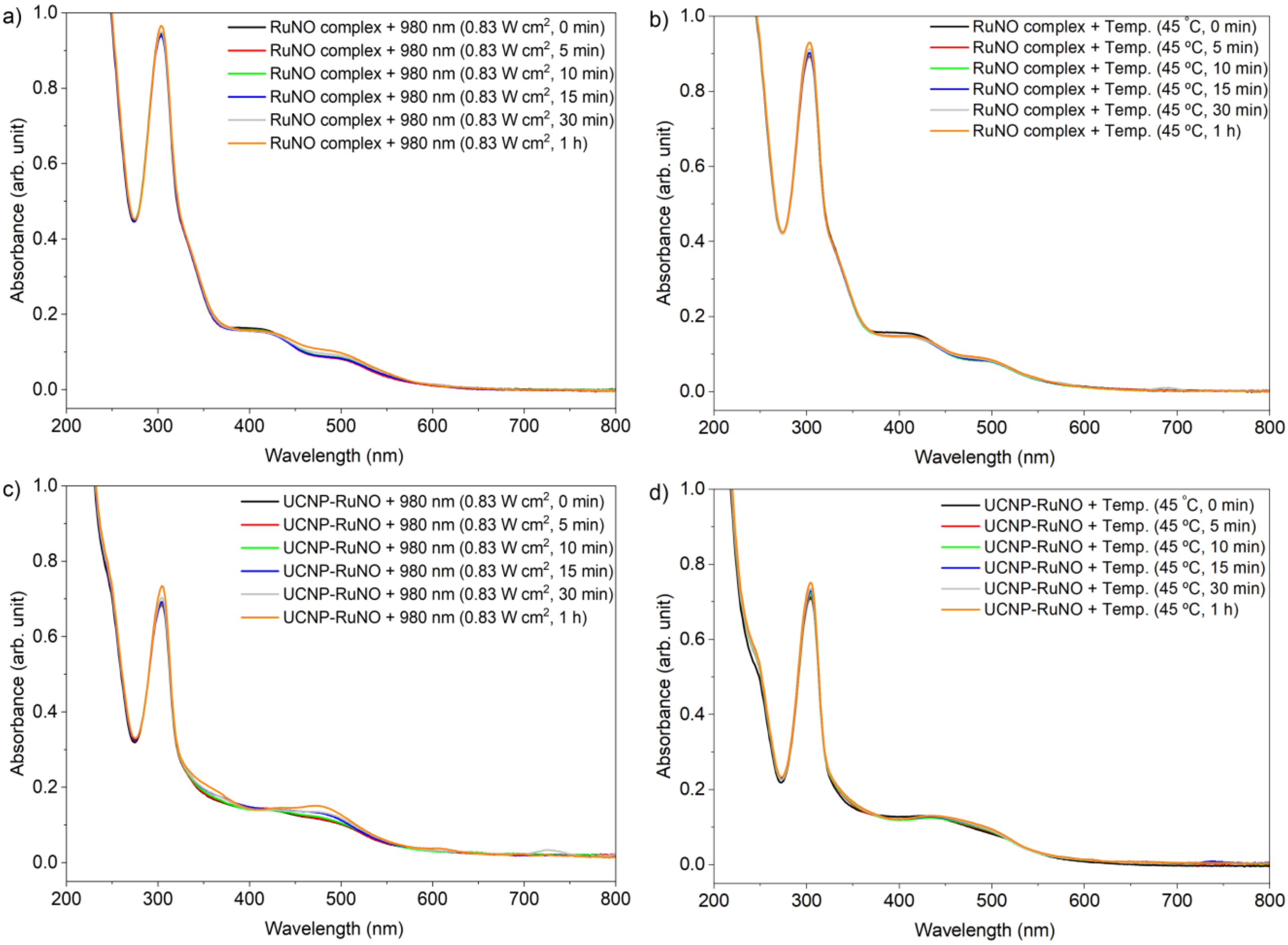

Figure S23. UV–Vis absorption spectra obtained in phosphate buffer solution (0.1 M, pH 7.4 at 25 °C) in the absence of cPTIO, used to evaluate the intrinsic spectral changes of the RuNO complex and UCNP–RuNO system under photochemical and thermal conditions: (a) RuNO complex under 980 nm irradiation (0.83 W cm^{-2}); (b) RuNO complex at 45 °C; (c) UCNP–RuNO under 980 nm irradiation (0. 83 W cm^{-2}); and (d) UCNP–RuNO at 45 °C. Spectra were recorded at 0, 5, 10, 15, 30, and 60 min.

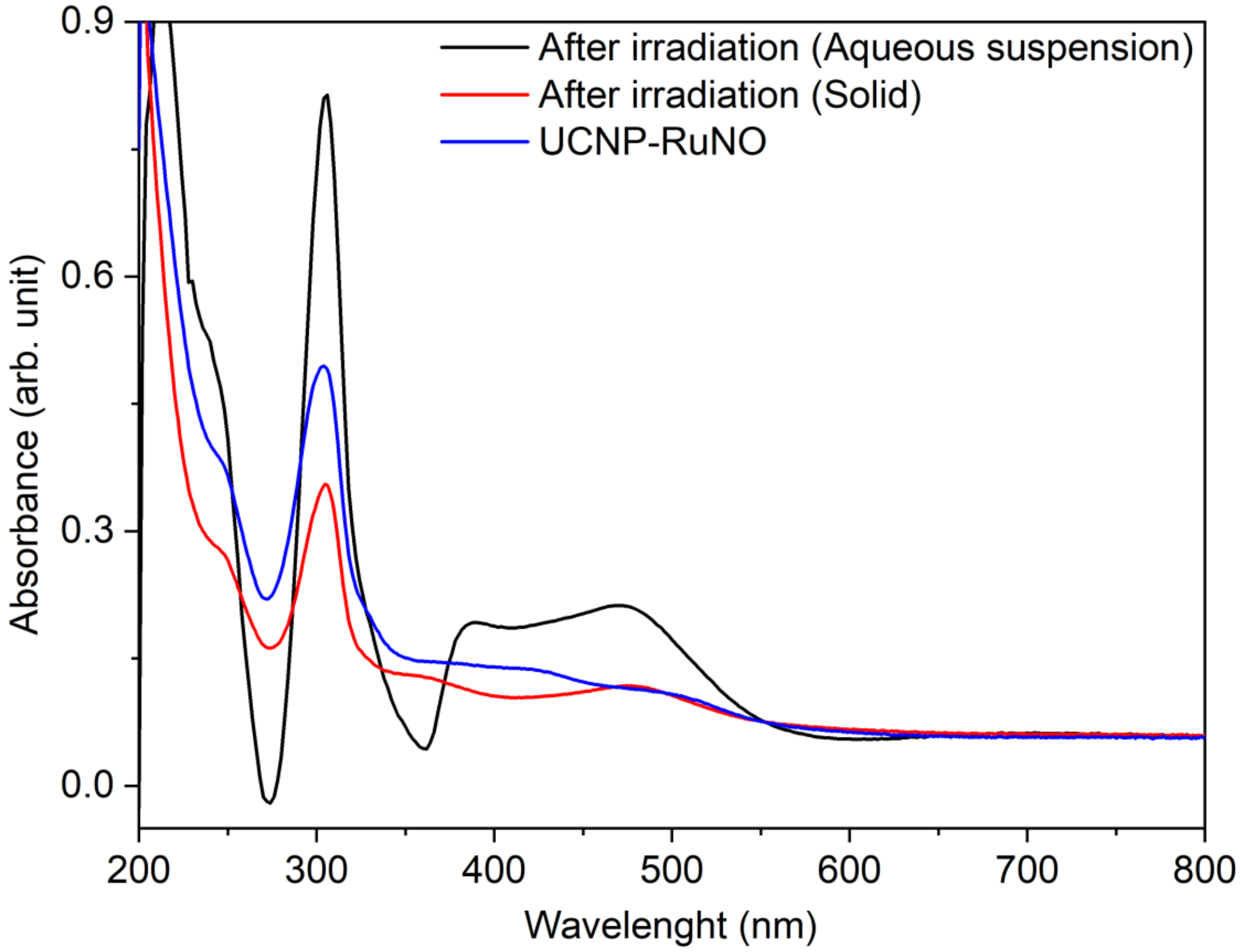

Figure S24. Non-normalized UV-Vis absorption spectra of UCNP-RuNO as prepared (blue line) and after irradiation in the solid state (red line) and in aqueous suspension (black line) with a NIR diode laser for 6 h.

REFERENCES

Codesido, N. O., Weyhermüller, T., Olabe, J. A., Slep, L. D. Nitrosyl-Centered Redox and Acid–Base Interconversions in [Ru(Me3[9]aneN3)(bpy)(NO)]3,2,1+. The pKa of HNO for its Nitroxyl Derivative in Aqueous Solution. Inorg. Chem., **2014**, 53, 2, 981–997. https://doi.org/10.1021/ic402448p

Durham, B., Walsh, J. L., Carter, C. L., Meyer, T. J. Synthetic Applications of Photosubstitution Reactions of Poly(pyridyl) Complexes of Ruthenium(II). Inorg. Chem., 19, 860–865, **1980**. https://doi.org/10.1021/ic50206a014.

Hao, C., Wu, X., Sun, M., Zhang, H., Yuan, A., Xu, L., Xu, C., Kuang, H. Chiral Core-Shell Upconversion Nanoparticle@MOF Nanoassemblies for Quantification and Bioimaging of Reactive Oxygen Species in Vivo. J. Am. Chem. Soc., 141, 19373–19378, **2019**. https://doi.org/10.1021/jacs.9b09360.

Hu, C., Huang, Y. C., Chang, A.-L., Nomura, M. Amine functionalized ZIF-8 as a visible-light-driven photocatalyst for Cr(VI) reduction. J. Colloid Interface Sci., 553, 372–381, **2019**. https://doi.org/10.1016/j.jcis.2019.06.040.

Park, K. S., Ni, Z., Côté, A. P., Choi, J. Y., Huang, R., Uribe-Romo, F. J., Chae, H. K., O'Keeffe, M., Yaghi, O. M. Exceptional chemical and thermal stability of zeolitic imidazolate frameworks. Proc. Natl. Acad. Sci. U.S.A., 103, 10310186–10191, **2006**. https://doi.org/10.1073/pnas.0602439103.

Silva, J. Y. R., Proenza, Y. G., Luz, L. L. da, Sousa Araújo, S. de, Filho, M. A. G., Junior, S. A., Soares, T. A., Longo, R. L. A thermo-responsive adsorbent-heater-thermometer nanomaterial for controlled drug release: (ZIF-8,EuxTby)@AuNP core-shell. Mater. Sci. Eng., C, 102, 578–588, **2019**. https://doi.org/10.1016/j.msec.2019.04.078.

Sprintschnik, G., Sprintschnik, H. W., Kirsch, P. P., Whitten, D. G. Preparation and Photochemical Reactivity of Surfactant Ruthenium(II) Complexes in Monolayer Assemblies and at Water-Solid Interfaces. J. Am. Chem. Soc., 99, 4947–4954, **1977**. https://doi.org/10.1021/ja00457a010.

Wu, Y., Wang, Y., Sun, Y., Li, Z., Li, X., Zhou, Z., Tang, D. Dissociation of Bipyridine and Coordination with Nitrosyl: Cyclometalated Ruthenium Nitrosyl Complex. Inorg. Chem., 61, 24, **2022**. https://doi.org/10.1021/acs.inorgchem.1c03770.
